# Bayesian Probabilistic Projection of International Migration

**DOI:** 10.1007/s13524-015-0415-0

**Published:** 2015-09-10

**Authors:** Jonathan J. Azose, Adrian E. Raftery

**Affiliations:** Department of Statistics, University of Washington, Box 354322, 98195-4322 Seattle, WA USA

**Keywords:** Autoregressive model, Bayesian hierarchical model, Markov chain Monte Carlo, World population prospects

## Abstract

We propose a method for obtaining joint probabilistic projections of migration for all countries, broken down by age and sex. Joint trajectories for all countries are constrained to satisfy the requirement of zero global net migration. We evaluate our model using out-of-sample validation and compare point projections to the projected migration rates from a persistence model similar to the method used in the United Nations’ *World Population Prospects*, and also to a state-of-the-art gravity model.

**Electronic supplementary material** The on line version of this article (doi:10.1007/s13524-015-0415-0) contains supplementary material, which is available to authorized users.

## Introduction

**Fig. 1:**
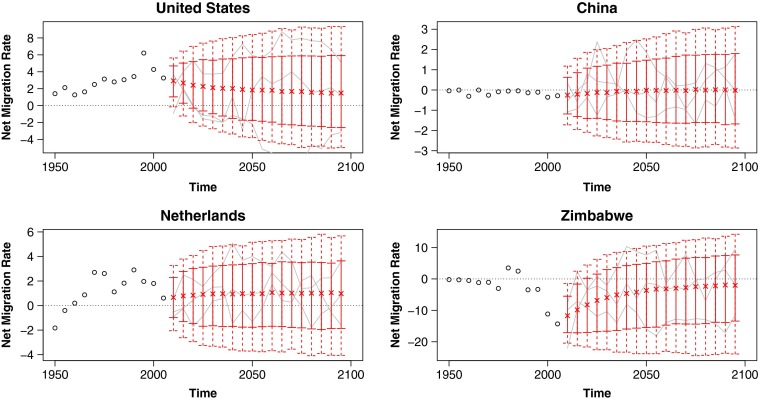
Probabilistic projections of net international migration rates: Predictive medians (indicated by “x”), 80 % (solid vertical lines), and 95 % (dashed vertical lines) prediction intervals for four countries, with example trajectories included in gray, and past observations shown as black circles. Rates are annualized and per thousand individuals in the specified country

In this article we propose a method for probabilistic projection of net international migration counts and rates. Our technique is a simple one that nonetheless overcomes some of the usual difficulties of migration projection. First, we produce both point and interval estimates, providing a natural quantification of uncertainty. Second, simulated trajectories from our model satisfy the common sense requirement that worldwide net migration sum to zero for each sex and age group. Third, our projected trajectories approximately replicate the observed frequency of countries switching between positive and negative net migration. Lastly, we sidestep the difficulty in projecting a complete large matrix of pairwise flows by instead working directly with net migration. Sample projections from our model for several countries are given in Fig. 1, and projected migration rates and counts for all countries are included as supplementary material in Online Resources 1 and 2.

In the remainder of the introduction, we provide background and describe global trends in migration. In the next section we describe our data and methods for producing probabilistic projections. This is followed by a summary of our main results, including an evaluation of our model’s performance and what our projections predict about future global migration trends. Finally, we conclude with evaluative discussion.

### Motivation and Background

There is a clear demand for migration projections. Organizations such as the United Nations, the UK Office for National Statistics, and the U.S. Social Security Administration have identified a necessity for migration forecasts (United Nations Population Division 2011; U.S. Social Security Administration 2013; Wright 2010).

Our work is motivated by the needs of the UN Population Division in producing probabilistic population projections for all countries. The UN has recently adopted a Bayesian approach to projecting the populations of all countries as the basis for its official medium projection, and issued probabilistic population projections for all countries for the first time in July 2014 (Raftery et al. 2014 United Nations Population Division 2012). The underlying method can account for uncertainty about fertility and life expectancy through Bayesian hierarchical models (Alkema et al. 2011; Raftery et al. 2012). However, the approach does not yet take account of uncertainty about international migration. Instead, the UN probabilistic population projections are conditional on deterministic migration projections that essentially amount to assuming that current migration levels will continue into the medium term. To make the method fully probabilistic would require probabilistic projections of net international migration for all countries.

Lutz and Goldstein (2004), in answering the question of how to deal with uncertainty in population forecasting, pointed to the need for simple approaches to probabilistic forecasting of migration. Our article attempts to meet this need. Despite the demand, some experts have been pessimistic about the possibility of predicting migration at all. For example, ter Heide (1963) argued that the task of finding a usable model for migration is “virtually impossible.” Bijak and Wiśniowski (2010) (2010:793–794) updated this opinion, drawing the similarly disheartening conclusions that “migration is barely predictable” and “forecasts with too long horizons are useless.”

Nevertheless, there have been efforts to forecast international migration. These attempts have mostly been limited in geographic and/or chronological scope. Bijak and Wiśniowski (2010) produced migration projections for seven European countries to 2025 using Bayesian hierarchical models. Using another geographically focused method, Fertig and Schmidt (2000) projected migration flows from a set of 17 mostly European countries to Germany over the 1998–2017 period. One drawback of these two approaches in the context of population projections for *all* countries is that both require the use of data on migration flows between pairs of countries. Estimates of reasonable quality of these flows are now available for most pairs of European countries (Abel 2010), making such techniques feasible for Europe and probably also for other developed regions. Estimates for global pairwise migration flows are also available (Abel 2013), but the quality of these estimates varies with the reliability of record keeping in the countries involved.

Hyndman and Booth (2008) provided another forecasting method: a stochastic model for indirect migration forecasting by forecasting fertility and mortality, with migration taken to be the appropriate quantity to satisfy the balancing equation. Their method provides estimates for individual countries for which reliable age- and sex-specific estimates of fertility, mortality, and migration are available. However, their method is not suitable for many of the world’s countries, where such detailed breakdowns are either unavailable or unreliable. The 2012 revision of the United Nations *World Population Prospects* (2013) took a simpler approach by including point projections that generally project migration counts to persist at or near current levels for the next couple of decades and drop deterministically to zero in the long horizon. Cohen (2012) provided a method for point projections of migration counts for all countries using a gravity model. See Bijak (2006) for a review of other methods.

### Theory of International Migration

There is a general consensus about the major causes of international migration. On the individual level, desire to migrate is caused largely by economic factors (Esipova et al. 2011; Massey et al. 1993). Refugee movements may be precipitated by political or social factors rather than economic ones (Richmond 1988). However, both economic and political factors are unlikely to be predictable in the long run with any useful degree of certainty. For the purposes of projection, Kim and Cohen (2010) argued for the use of more predictable demographic variables in place of less predictable economic ones. They proposed a model for prediction of migration flows that incorporates life expectancy, infant mortality rate, and potential support ratio as predictor variables. Kim and Cohen (2010) found these variables to be significant predictors of migration flows. Furthermore, because demographic variables tend to change much more slowly than economic or political ones, it is often possible to project the values of demographic variables decades into the future with less uncertainty. Our model projects net migration on the basis of only past migration figures and an initial projection of populations for all countries, for which forecasts can be made with enough precision to be useful.

One additional demographic variable of interest in modeling migration is age structure, which is important to migration modeling in two different ways. First, projected age structures for all countries can potentially be used as predictor variables in projections of future migration. Because labor migration is common, the age structure of the sending and/or receiving countries can be used in making projections (Fertig & Schmidt 2000; Hatton & Williamson 2002, 2005). Kim and Cohen (2010), in a study of pairwise migration flows, found that a young age structure in the country of origin is associated with high migration flows, while a young age structure in the country of destination is associated with low flows.

Second, it may be of interest to project not only net migration counts but also *age-specific* net migration counts. Rogers and Castro (1981) provided a parametric multiexponential model migration schedule that can be used in converting from projected net migration counts to age-specific counts. Their model incorporates a principal migration peak among young adults, who often migrate for reasons of economics, marriage, or education, as well as a secondary childhood peak for the children of those young adult migrants. The model includes an additional option for waves of retirement and post-retirement migration, which are common patterns of regional migration but are less common internationally. Use of these model migration schedules can be particularly problematic when working with net migration rather than inflows and outflows (Rogers 1990), but they may still provide a first-order approximation of age structures when no better data are available. Raymer and Rogers (2007) noted the complication that the age structure of a migrating population is dependent on direction of migration. For example, we would expect a labor migration and a subsequent return migration to have different age structures. This can be taken into account to some extent in a model like ours if data on the age structure of recent net migration are available.

For projection purposes, Bayesian modeling is well suited to modeling international migration. The difficulty in making accurate point projections emphasizes the need for an approach that produces estimates of uncertainty. Because our data set includes only 12 time points per country, non-Bayesian inference could be difficult; the Bayesian approach alleviates this by allowing us to borrow strength across countries and to incorporate prior knowledge. Studies with limited geographical scope confirm this intuition. In a comparison of several methods for forecasting migration to Germany, Brücker and Siliverstovs (2006) found performance of a hierarchical Bayes estimator to be superior to that of simpler estimators based on ordinary least squares regression, fixed effects, or random effects. Well-calibrated results have come out of Bayesian forecasting efforts for fertility and mortality (Alkema et al. 2011; Raftery et al. 2012, 2013). In addition to forecasting, estimation of demographic variables also lends itself to Bayesian methodology (Abel 2010; Congdon 2010; Wheldon et al. 2013).

## Methods

### Data

We use data from the 2010 revision of the United Nations Population Division’s biennial *World Population Prospects* (WPP) report (United Nations Population Division 2011). WPP reports contain estimates of countries’ past age- and sex-specific fertility, mortality, and net international migration counts and rates, as well as projections of future migration.

Our work is motivated by a desire to incorporate probabilistic migration projections into probabilistic population projections. Thus, the quantity we are interested in forecasting is *y*_*c*,*t*_, the net number of migrants in country *c* in time period *t*. Because net migration is sufficient to determine population change due to migration, we need not consider inflows and outflows separately. We condition on known population projections, $\tilde n_{c,t}$, taken from the WPP 2010 revision. So long as projected populations are known, we can freely convert between net migration counts, *y*_*c*,*t*_, and net migration rates, *r*_*c*,*t*_. In the WPP data, rates are reported in units of migrants per thousand individuals in the specified country.^1^

### Probabilistic Projection Method

Our technique is to fit a Bayesian hierarchical first-order autoregressive, or AR(1), model to net migration *rate* data for all countries. Recall that our motivation is to obtain probabilistic migration projections for incorporation into population projections for all countries—an application that requires projected net migration *counts* rather than *rates*. Nevertheless, it is advantageous to model on the rate scale and convert the output to counts rather than modeling counts directly. The primary disadvantage to modeling net migration counts is that variability in count data grows roughly in proportion to population size. This suggests dividing counts by population sizes as a way of stabilizing the variance, resulting in a model on migration rates.

We model the migration rate, *r*_*c*,*t*_, in country *c* and time period *t* as

$$(r_{c,t}-{\upmu}_{c})={\upphi}_{c}(r_{c,t-1}-{\upmu}_{c})+\upvarepsilon_{c,t}, $$

where ε_*c*,*t*_ is a normally distributed random deviation with a mean of zero and a variance of ${{\upsigma }^{2}_{c}}$. We put normal priors on each country’s theoretical long-term average migration rate μ_*c*_, and a uniform prior on the autoregressive parameter ϕ_*c*_. Under this model, simulation of trajectories requires us to estimate or specify values of μ_*c*_,ϕ_*c*_, and ${{\upsigma }^{2}_{c}}$ for all countries; thus, the complete parameter vector is given by $\boldsymbol {\uptheta }=({\upmu }_{1}, \ldots , {\upmu }_{C}, {\upphi }_{1}, \ldots , {\upphi }_{C}, {{\upsigma }^{2}_{1}}, \ldots , {{\upsigma }^{2}_{C}})$, where *C* is the number of countries.

The full specification of the model, including prior distributions, is as follows:^2^

$$\textrm{Level 1} \left\{ \begin{array}{l} (r_{c,t}-{\upmu}_{c})={\upphi}_{c}(r_{c,t-1}-{\upmu}_{c})+\upvarepsilon_{c,t}\\ \upvarepsilon_{c,t} \overset{\text{ind}}{\sim} N(0,{{\upsigma}^{2}_{c}}) \end{array} \right. $$

$$\textrm{Level 2} \left\{ \begin{array}{l} {\upphi}_{c} \overset{\text{iid}}{\sim} U(0,1)\\ {\upmu}_{c} \overset{\text{iid}}{\sim} N(\uplambda, {\uptau}^{2})\\ {{\upsigma}^{2}_{c}} \overset{\text{iid}}{\sim} IG(a,b)\\ \end{array} \right. $$

$$\textrm{Level 3} \left\{ \begin{array}{l} a \sim U(1,10)\\ b|a \sim U(0,100(a-1))\\ \uplambda \sim U(-100,100)\\ \uptau \sim U(0,100), \end{array} \right. $$

where *X*∼*N*(μ,σ^2^) indicates that the random variable *X* has a normal distribution with a mean of *μ* and a variance of σ^2^ (and hence a standard deviation of σ), *U*(*c*,*d*) denotes a uniform distribution between the limits *c* and *d*, and *I* *G*(*a*,*b*) denotes an inverse gamma distribution with probability density function (as a function of *x*) proportional to *x* ^−*a*−1^ *e* ^−*b*/*x*^.

We obtain draws from the posterior distributions of all parameters using Markov chain Monte Carlo methods. In our implementation, we use the Just Another Gibbs Sampler (JAGS) software package for Markov chain Monte Carlo simulations (Plummer 2003).

Having obtained a sample (***𝜃***_1_,…,***𝜃***_*N*_) of draws from the joint distribution of the parameters, we use these draws to obtain a sample from the joint posterior predictive distribution. For each sampled value ***𝜃***_*k*_ from the joint posterior distribution of the parameters, we first simulate a set of joint trajectories $\tilde r_{c,t}^{(k)}$ for net migration rates at time points until 2100, where *k* indexes the trajectory. However, this procedure generally produces trajectories that are impossible in that they give nonzero global net migration counts. We therefore create corrected trajectories for net migration counts and rates using the following method:

1. On the basis of the parameter vector ***𝜃***_*k*_, project net migration rates for all countries a single time point into the future. Denoting the next time period in the future by *t* ^′^, this allows us to obtain a collection of (uncorrected) projected values $\tilde r_{c,t^{\prime }}^{(k)}$ for all countries *c*.
2. Convert net migration rate projections $\tilde r_{c,t^{\prime }}^{(k)}$ to net migration count projections $\tilde y_{c,t^{\prime }}^{(k)}$. To convert from rates to counts, we multiply the rate $r_{c,t^{\prime }}^{(k)}$ by the projected average population. Projected average populations are taken from the deterministic population projections in WPP 2010 (United Nations Population Division 2011).
3. Further break down migration counts by age *a* and sex *s* to obtain estimates of net male and female migration counts for all countries and age groups, $\tilde y_{c,t^{\prime }\!\!,a,s}^{(k)}$. This is done by applying projected migration schedules to all countries. For the projections in this article, we take each country’s projected age- and sex-specific migration schedule to be the same as the distribution of migration by age and sex in the most recent time point for which detailed data are available for that country.
4. For each simulated trajectory, within each age and sex category, apply a correction to ensure zero worldwide net migration. The correction we apply redistributes any overflow migrants to all countries, in proportion to their projected populations. Specifically, we take the corrected migration count projection $\tilde y_{c,t^{\prime }\!\!,a,s}^{*(k)}$ to be
  $$\tilde y_{c,t^{\prime}\!\!,a,s}^{*(k)}=\tilde y_{c,t^{\prime}\!\!,a,s}^{(k)}-\frac{\tilde n_{c,t^{\prime}}}{{\sum}_{j=1}^{C} \tilde n_{j,t^{\prime}}} {\sum}_{j=1}^{C} \tilde y_{j,t^{\prime}\!\!,a,s}^{(k)}.$$
5. Convert the corrected age- and sex-specific net migration counts $\tilde y_{c,t^{\prime }\!\!,a,s}^{*(k)}$ back to corrected net migration rates $\tilde r_{c,t^{\prime }}^{*(k)}$ by aggregating and converting counts to rates. In practice, the corrections from the previous step are typically small on the net rate scale. In more than 95 % of cases, the resulting change in countries’ projected net migration rates $\tilde r_{c,t^{\prime }}^{*(k)}$ is less than 0.2 net annual migrants per thousand.
6. Continue projecting trajectories one time step at a time into the future by repeating steps 1–5.

Although the uncorrected net migration rates $\tilde r_{c,t^{\prime }}$ come from the desired marginal posterior predictive distributions, the correction in step 4 changes those distributions by projecting them onto a lower-dimensional space. Sensitivity analysis suggests that the correction introduces only minor changes between the marginal distributions with and without the correction.

Also worth noting is that the projected net migration rates from our method are not very sensitive to changes in the population projections $\tilde n_{c,t^{\prime }}$, justifying the use of fixed WPP 2010 population projections that include migration. It would be possible instead to project all components of population change simultaneously, including migration.

Probabilistic projections of net migration rates and counts for all countries for the time periods from 2010 to 2100 are included as Online Resources 1 and 2.

## Results

### Evaluation

We evaluate projections in the form of net migration rates. In the modeling stage, the choice to use rates rather than counts was mathematically motivated. A model on net migration counts would have required variance proportional to population size, a complication that is not necessary on the rate scale. The choice to evaluate on rates rather than counts is motivated by the application we have in mind. The goal is to produce migration projections for all countries in response to the needs of the UN Population Division. Evaluation on the count scale would effectively give heavy weight to our model’s performance on a small number of high-migration countries. To better assess performance on all countries, we work instead on the rate scale.

We do not know of any other model that produces *probabilistic* projections of net migration for all countries. However, we can take our model’s median projections to be point projections and compare them with models that produce point projections only. First, as a baseline for comparison, we evaluate them against simple *persistence models,* which project either net migration rates or net migration counts to continue at the most recently observed levels indefinitely into the future. For up to 35 years into the future, the model that projects persistence of net migration counts is similar to the expert knowledge-based projections in the WPP (United Nations Population Division 2011).

Second, we compare against point projections produced separately for all countries using Cohen’s (2012) gravity model-based method. The gravity model produces projected migration counts, which we convert to rates for evaluation. For each country *c*, the gravity model makes projections as follows: let *L*(*t*) be the population of country *c* at time *t*, and let *M*(*t*) be the population of the rest of the world at time *t*. Then expected in-migration to country *c* is given by *a*×*L*(*t*)^α^ *M*(*t*)^β^, where *a* is a country-specific proportionality constant. The exponents *α* and β are constant across countries, with values estimated by Kim and Cohen (2010). Similarly, expected out-migration from country *c* has the form *b*×*L*(*t*)^γ^ *M*(*t*)^δ^, where *b* is to be estimated, and γ and δ come from Kim and Cohen (2010). The constants of proportionality *a* and *b* for each country are chosen to minimize the sum of squared deviations between estimates of net migration produced by the gravity model and historical values of net migration from the WPP 2010 revision (United Nations Population Division 2011). Having estimated *a* and *b* for a particular country, we calculate net migration projections by *a*×*L*(*t*)^α^ *M*(*t*)^β^−*b*×*L*(*t*)^γ^ *M*(*t*)^δ^, where *L*(*t*) and *M*(*t*) are now projected populations. Implementation details are given in Appendix 1A.

Our historical data consist of a series of migration rates *r*_*c*,*t*_ for 197 countries at 12 time points in five-year time intervals, spanning the period from 1950 to 2010. We perform an out-of-sample evaluation by holding out the data from the *m* most recent time points for all countries and producing posterior predictive distributions on the basis of the remaining (12−*m*) time points. As point forecasts, we used the median of the posterior predictive distribution. We report out-of-sample mean absolute error as a measure of the quality of point forecasts, and interval coverage as a measure of quality of our interval predictions.

Table 1 contains these evaluation metrics for our Bayesian hierarchical model and the mean absolute errors for the persistence and gravity models. Our point projections outperformed the gravity model and both persistence models at all forecast lead times, and our interval projections achieved reasonably good calibration. Appendix 2B contains additional tables with evaluation metrics broken down by region. Our Bayesian hierarchical model outperformed the gravity model in all regions and the persistence models in most regions.

**Table 1:** Predictive performance of different methods: Mean absolute errors (MAE) and prediction interval coverage for our Bayesian hierarchical model, the gravity model, and the persistence models

Validation Time Period	Model	MAE	80 % Cov. (%)	95 % Cov. (%)
5 Years	Bayesian	3.24	91.4	96.4
	Gravity	4.70	—	—
	Persistence (of rates)	3.57	—	—
	Persistence (of counts)	3.58	—	—
15 Years	Bayesian	4.76	84.9	93.4
	Gravity	6.57	—	—
	Persistence (of rates)	6.74	—	—
	Persistence (of counts)	6.30	—	—
30 Years	Bayesian	5.12	77.2	89.3
	Gravity	12.32	—	—
	Persistence (of rates)	7.17	—	—
	Persistence (of counts)	5.82	—	—

### Migration Trends

The primary goal of our model is to produce point and interval projections. However, it is also desirable for our model to replicate current trends in the migration data.

One prominent feature of the historical migration data to consider is the frequency with which countries switch between being net senders and net receivers of migrants. Such switches have been relatively common over the past 50 years. In fact, in the 2005–2010 period, 46 % of countries had different migration parity than they had in 1955–1960 (i.e., they switched either from net senders to net receivers or vice versa). In contrast, the current United Nations methodology (United Nations Population Division 2013) projects *no* crossovers between now and 2100. Our model projects crossover behavior that is more in line with historical trends. Further analysis of projected parity changes is given in the case study on Denmark later in the article.

A second question is what our projections say about the *magnitude* of migration. Because we have only directly modeled net migration counts *y*_*c*,*t*_ and the associated rates *r*_*c*,*t*_, looking at the associated magnitudes |*y*_*c*,*t*_|, or equivalently |*r*_*c*,*t*_|, can serve as a model validity check. For example, a model could produce reasonable marginal migration projections for all countries despite being consistently biased toward projecting too much migration. We think it is worth confirming that our model does not have such a fault.

Furthermore, the analysis in the Evaluation section was concerned only with *marginal* projections for each country. However, because our projections actually take the form of joint trajectories for all countries simultaneously, we should confirm that the joint projections look reasonable. We do so by condensing high-dimensional joint projections of absolute migration into a single dimension using two different averages of net absolute migration.

One meaningful average of absolute net migration rates is

$$u(t)=\frac{{\sum}_{c\;=\;1}^{C} |r_{c,t}|}{C},$$

the *unweighted* mean absolute net migration rate across all countries. Because net migration represents the contribution of migration to population change, *u*(*t*) can be interpreted as a heuristic measure of whether it is typical for countries to experience a lot of population change from the effects of migration.

Weighting absolute migration rates in proportion to population size, rather than uniformly, produces a measure of what the typical individual experiences, rather than the typical country. Such a weighted average is given by

$$w(t) = \sum\limits_{c\;=\;1}^{C} |r_{c,t}| \frac{n_{c,t}}{{\sum}_{j\;=\;1}^{C} n_{j,t}}. $$

**Fig. 2:**
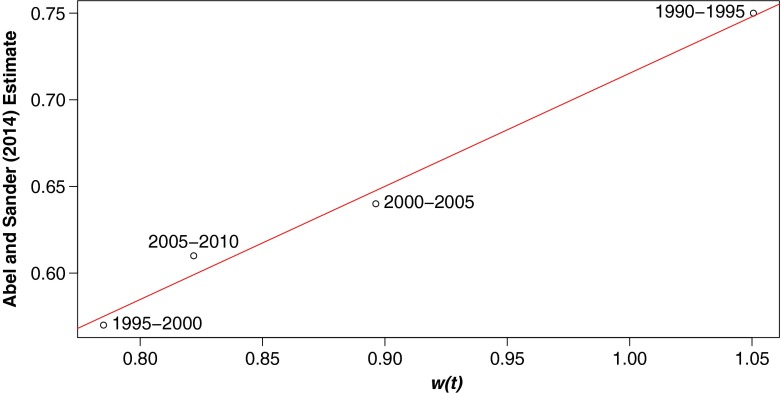
Estimated percentage of the world population migrating compared with a population-weighted average of absolute net migration rates, *w*(*t*), for five-year periods from 1990 to 2010. Estimates of percentage of world population migrating are taken from Abel and Sander (2014). For this figure, we convert rates from the usual “net annual migrants per thousand” to “net five-year migrants per hundred” to put them on a comparable scale with percentage of world population migrating. There is a strong and significant correlation between the two quantities (*R* ^2^=.989, *p*=.006)

If it were true that countries with net outflows had no inflows and vice versa, then $\frac {1}{2}w(t)$ would give the total proportion of the world population migrating. Of course, substantial cross-flows are common, so in reality, $\frac {1}{2}w(t)$ substantially underestimates the total proportion of the world population migrating. Nevertheless, comparison with flow estimates for 1990–2010 from Abel and Sander (2014) shows that *w*(*t*) is strongly correlated with the total proportion of the world population migrating. Figure 2 compares Abel and Sander’s estimates with *w*(*t*). The correlation between the two measures is strong and significant (*R* ^2^=.989, *p*=.006).

**Fig. 3:**
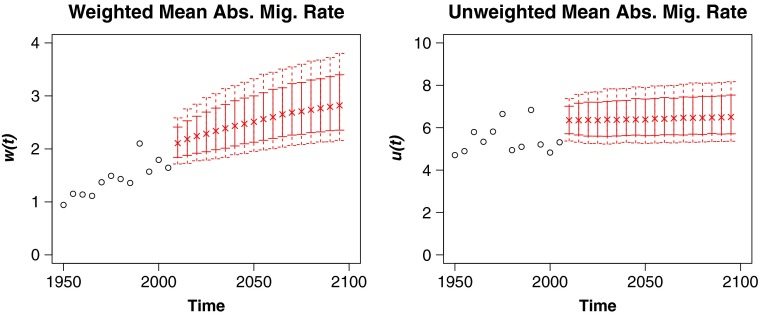
Observed historical data on population-weighted (left) and unweighted (right) averages of absolute annual migration rates per thousand for five-year periods from 1950 to 2010 (indicated by circles). Median estimates (indicated by “x”) and 80 % and 95 % prediction intervals (indicated with vertical lines) from our model for periods out to 2100

Figure 3 shows the historical values of *u*(*t*) and *w*(*t*) as well as our projections into the future. Our forecast shows no clear growth or shrinkage in *u*(*t*), which is consistent with its historical trend. Meanwhile, we predict that *w*(*t*) will continue to grow, leveling off in the long horizon. Despite the apparent contradiction, there is no inconsistency in the fact that *w*(*t*) has grown quite substantially over time while *u*(*t*) has not. This discrepancy is largely explained by the facts that (1) the largest countries have experienced mild increases in their absolute migration rates over time and (2) net migration rates and counts in the Gulf States grew enormously over this period. This first observation can be viewed as evidence of a form of globalization in international migration, in which net migration rates for large countries, once very low, are becoming more similar to those for other countries.

### Case Studies

We now examine projected migration rates for a selection of four countries: Denmark, Nicaragua, India, and Rwanda. These four countries were selected both to provide geographic diversity and a variety of observed net migration trends since the 1950s. Denmark has experienced a shift from being a net sender of migrants to a net receiver, a pattern common in European countries. Nicaragua has had relatively stable and consistently negative net migration since the 1950s. India has had migration rates close to zero, which is common among the largest countries. Finally, Rwanda provides an example of a country that has experienced a large spike in absolute migration rate. This is not intended to be an exhaustive catalog of observed trends in net migration rates, although many countries have followed patterns similar to one of these four example countries.

Following these four case studies, we also present projections for the least-developed countries versus all other countries.

#### Denmark

Denmark experienced net emigration through the 1950s but has consistently received net immigration since the 1960s. This pattern of changing from a net sender to a net receiver within the last 60 years is common to many European countries, including Norway, Finland, the UK, and Spain. These countries’ net out-migration in the middle of the twentieth century serves as a reminder that the global migration to northern and western Europe, which now seems so firmly established, is a relatively recent phenomenon.

**Fig. 4:**
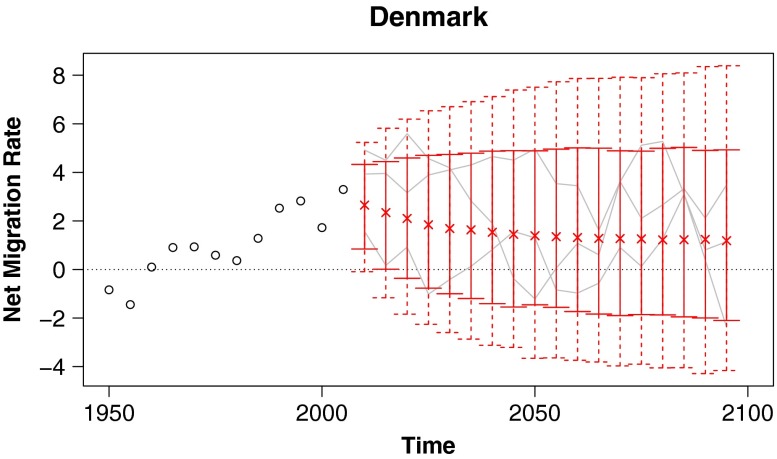
Probabilistic projections of net international migration rates: Predictive medians and 80 % and 95 % prediction intervals for Denmark, with example trajectories included in gray

Our median predictions for Denmark have the country continuing to be a net receiver of migrants for as far out into the future as we care to project (Fig. 4). However, we also see that the probability of Denmark switching over to a net sender increases over time. Based on the history of the twentieth century, it seems realistic to include the possibility of changeovers in Denmark and other European countries in probabilistic migration projections. Correspondingly, projections that do not take account of this possibility seem unrealistic.

The European countries are not alone in having oscillated between being net senders and net receivers of migrants. As mentioned in the discussion of migration trends, 46 % of countries had different migration parity in the 1955–1960 period than they had in 2005–2010. Thus, they switched either from net senders to net receivers, or vice versa, during the past 55 years. Our Bayesian hierarchical model projects that 46 % of countries will have different migration parity in 55 years (i.e., in 2055–2060) than they do now.^3^ This projection is in line with the number of historical parity changes. In contrast, the gravity model (Cohen 2012) projects that only 29 % of countries will change parity by 2055–2060. Both persistence models and the WPP migration projections (United Nations Population Division 2013) project no parity changes.

#### Nicaragua

**Fig. 5:**
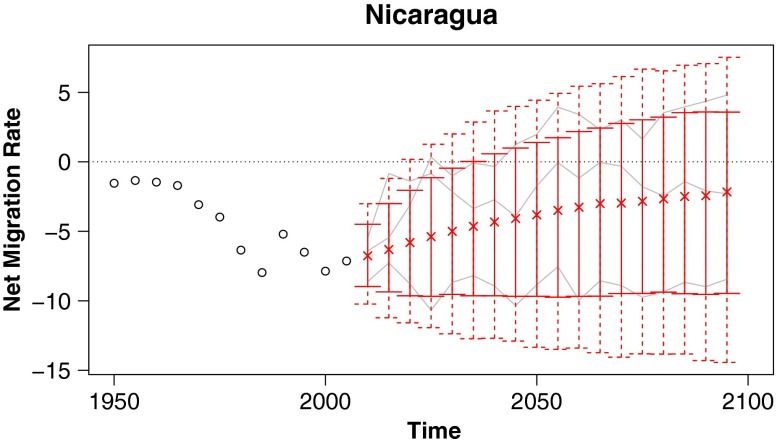
Probabilistic projections of net international migration rates: Predictive medians and 80 % and 95 % prediction intervals for Nicaragua, with example trajectories included in gray

Migration rates in Nicaragua have increased steadily in magnitude over the last six decades. Nevertheless, although our model projects a small probability of continued growth in the magnitude of the net migration rate, it gives higher probability to scenarios in which migration rates move back toward zero (Fig. 5). In general, our model favors trajectories in which net migration rates move toward zero rather than continuing current trends of growth in magnitude where such trends exist.

Statistically, this tendency for migration rates on average to reverse course and tend back toward zero reflects past trends through from the hierarchical nature of the model. Specifically, all of the μ_*c*_ values, which we can think of as the long-horizon median migration rates for each country, are assumed to come from a common *N*(λ,τ^2^) distribution. As a result, the hierarchical “borrowing of strength” has a tendency to pull all the μ_*c*_ values toward a common center, λ, which has a posterior distribution with a mode close to zero. Although our model’s median projections tend to predict reversal in growth trends, the predictive probability distributions give substantial probability to continuation and also to growth of rates.

#### India

**Fig. 6:**
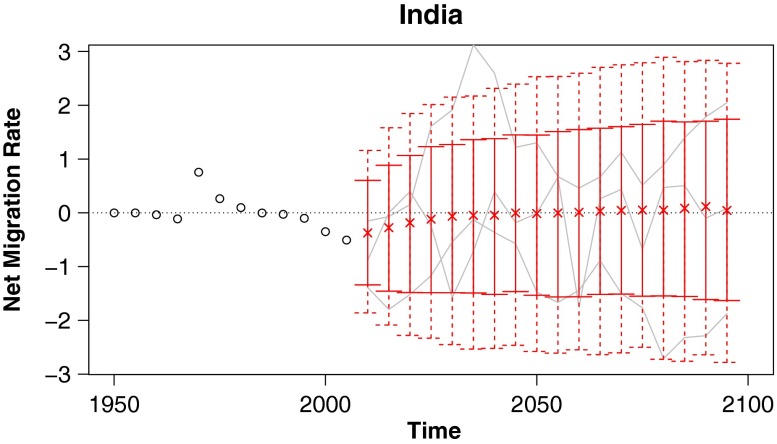
Probabilistic projections of net international migration rates: Predictive medians and 80 % and 95 % prediction intervals for India, with example trajectories included in gray

Historically, India has had relatively low net migration rates, on the order of less than 1 per thousand. The 95 % prediction intervals from our model are quite a bit wider than the range of India’s historical data, expanding out to roughly ±3 per thousand (Fig. 6).

Statistically, the width of a country’s prediction intervals from our model is primarily controlled by the error variance ${{\upsigma }^{2}_{c}}$. (The autoregressive parameters, ϕ_*c*_, also influence the width of prediction intervals, but to a lesser extent.) The excess width of India’s prediction intervals above its range of observed migration history is statistically a result of the hierarchical “borrowing of strength.” Given that most other countries have larger ranges of migration rates, the posterior distribution of ${{\upsigma }^{2}_{c}}$ for India gets inflated somewhat to values more in line with the rest of the world. The same inflation of ${{\upsigma }^{2}_{c}}$ occurs in China, which has also experienced uncommonly low migration rates in the past.

Substantively, this seems realistic given the increasing globalization we have documented. As the largest countries become more like other countries in terms of migration patterns, it seems reasonable to expect that the variability of their migration rates in the future would also increase to become more like the levels of other countries.

#### Rwanda

In the early 1990s, Rwanda experienced high net out-migration, followed by high net in-migration in the late 1990s. These migration spikes were a result of emigration during the Rwandan genocide in 1994 and subsequent return migration. Outside of the 1990s, Rwanda had quite small and stable migration rates. This pattern of stability punctuated by large shocks poses a problem for probabilistic projections: Do we get better performance with wide prediction intervals that encompass the high migration rates during the shock, or narrow prediction intervals that reflect the decades of stability around it?

**Fig. 7:**
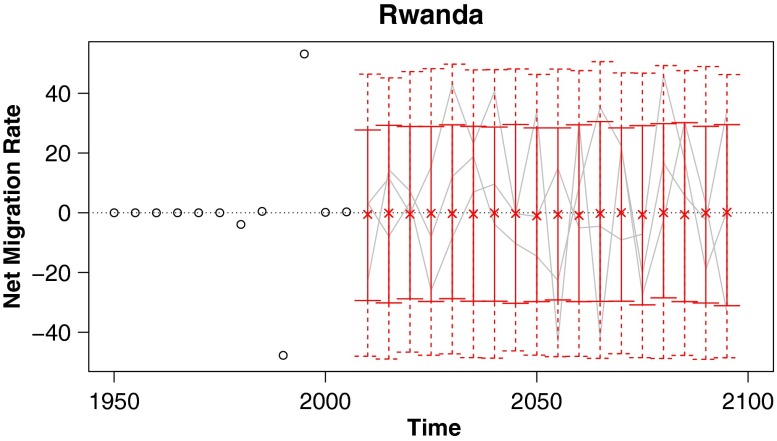
Probabilistic projections of net international migration rates: Predictive medians and 80 % and 95 % prediction intervals for Rwanda, with example trajectories included in gray

Our model opts for wide prediction intervals in cases like Rwanda (Fig. 7). A model that puts a heavy-tailed *t* distribution on the random error terms ε_*c*,*t*_ rather than a normal distribution would produce narrower prediction intervals. However, we found that the normal model achieved better calibration of the main prediction intervals of interest—namely those of probability 95 % and lower. The concluding discussion section contains a brief further discussion of a model with *t*-distributed errors.

#### The Least-Developed Countries

The United Nations publishes a list of the least-developed countries, with countries classified as least-developed based on assessments of their economic vulnerability, human capital, and gross national income (Committee for Development Policy and United Nations Department of Economic and Social Affairs 2008). A total of 46 countries in our data fall into the least-developed category. We now consider briefly the projections that our model makes for these least-developed countries in comparison to all other countries.

In the 2005–2010 period, only 26 % of the least-developed countries were net receivers of migration, as compared with 43 % of all other countries. The least-developed countries had an average net migration rate of −0.97 per thousand, compared with an average of 2.64 per thousand in all other countries. However, our model projects that this gap in migration between currently least-developed and all other countries will narrow over time. Key findings are summarized in Table 2. Over the coming decades, on average, we project mild growth in net migration rates among the least-developed countries and decline in net migration rate across all other countries.

**Table 2:** Mean projected change in migration rates (per thousand) among least-developed countries (LDC) versus all other countries (other), with 95 % prediction intervals in parentheses

	LDC	Other
By 2020	+0.02 (−3.09,+2.99)	−1.50 (−3.24,+0.33)
By 2040	+0.23 (−2.89,+3.38)	−2.12 (−4.23,+0.06)
By 2060	+0.35 (−2.78,+3.55)	−2.29 (−4.54,+0.14)

## Discussion

We have presented a method for projecting net international migration rates. Our method is novel in that it provides probabilistic projections of net migration for all countries. Furthermore, it satisfies the requirement that simulated trajectories have zero global net migration for each sex and age group.

Additionally, we observe a paradoxical trend in the evolution of global migration rates. Although there appears to be more migration than in the past as a proportion of the world population, countries, absolute migration rates, on average, have not been increasing. Our method successfully reproduces this pattern, which seems desirable for migration projection methods in general.

Our model includes the assumption that the random error terms ε_*c*,*t*_ are independent across countries and time. That assumption is mathematically convenient, but for many pairs of countries, we expect to see nonzero correlations. For example, it is reasonable to expect that if Mexico undergoes particularly high net emigration during a quinquennium, then the United States will experience higher than usual net immigration during the same period. Thus, we might expect to observe negative correlation between the random errors for Mexico and the United States. At the same time, it is not unreasonable to expect positive correlation between error terms in neighboring pairs of countries whose economic fortunes tend to move together. Such a pattern is observed, for example, among the Baltic states. We attempted to find an optimal nontrivial covariance structure by constructing a variance-covariance matrix as a linear combination of matrices whose off-diagonal elements are pairwise, time-invariant covariates. However, this method offered no significant improvement over the assumption of independent residuals.

Migration data characteristically have outliers. Wars and refugee movements, for example, produce migration rates that are on a much larger scale than are typical during times of stability. This suggests that a model with a long-tailed error distribution, such as a *t* distribution, might be more appropriate than a model with normal errors. Furthermore, a model with *t*-distributed errors with degrees of freedom allowed to vary across countries is a natural way of handling the fact that some regions have quite stable migration rates over time (e.g., Western Europe) while others have quite a lot of volatility (e.g., Central Africa). However, in practice, we found that models with normally distributed errors tended to outperform models with *t* errors in out-of-sample evaluation of the resulting prediction intervals. Models with *t* errors often produce 80 % and 95 % prediction intervals that are so tight that they do not come close to covering the range of observed historical migration rates.

Statistically, the root of the problem is that in models with *t* errors, large outliers often do not have a large effect on the inferred scale parameter. Although using *t* errors often results in models with a high likelihood of the observed data, high likelihood does not necessarily correspond to good calibration of prediction intervals or qualitatively realistic migration rates. For the migration forecasting problem, we believe that there is more value in forecast distributions with reasonable prediction intervals than in distributions that are likely to assign high probability density to future observations, if the choice has to be made. Thus, we used the normal model thoughout.

Note that by selecting the AR(1) model in advance, we are necessarily not accounting for variance due to model uncertainty. In the short term, this approach can be empirically justified by the fact that recent data are fit relatively well by the AR(1) model. If, in the long term, this ceases to be true, we expect our model to understate variances of posterior predictive distributions. Abel (2013) demonstrated a Bayesian approach of averaging population forecasts across several plausible time series models that could be suitable to our data. We did not take this approach here because of empirical findings that higher-order autoregressive models don’t offer significant increase in predictive power on this data set and because the AR(1) model offers qualitatively plausible long-term prediction intervals. However, it is quite possible that expanding our current method to take account of model uncertainty would improve the quality of the predictions.

An additional source of variance which is unaccounted for in this article is the uncertainty in projected populations. The results presented here are conditional on the deterministic population projections in WPP 2010. Our methodology could be straightforwardly modified to allow instead for probabilistic population projections as inputs, including population projections that are updated at each time step to incorporate the migration projections output by our model. The analysis presented here, however, is focused on producing reasonable migration projections taking known population projections as a given.

Our migration projections, which are conditional on projected population, suggest the possibility of allowing migration to influence projected population within each trajectory. We expect such an exercise would require additional constraints to ensure that migration trajectories cannot result in unreasonable population outcomes. Of particular concern are the possibilities of projecting total depopulation, as we might in Pacific Island countries with historically large out-migration rates, or projecting impossibly high population booms, as we might in the Middle East. One possible solution to this problem could be to rule out any trajectories that project sustained periods of in- or out-migration that are too lengthy or too large in magnitude.

The ultimate goal of this work is to produce probabilistic projections of net migration counts aggregated at the country level and over a long time scale for integration into probabilistic population projections. Several simple modifications can be made if the reasons for wanting projections are different. If age- and sex-specific net migration counts are of primary interest, a more-nuanced handling of migration schedules can replace our simplistic method of assuming that current migration schedules will persist into the future. In order to produce long-term projections, we did not include potentially relevant economic and political covariates because such covariates are hard to predict in the far future. If only short-term projections are desired, it would be possible to introduce these covariates into the model as well. Adding such covariates could improve short-term predictive ability at the possible expense of misestimating variability in migration predictions if we fail to correctly estimate variability in the covariates.

### Electronic supplementary material

Below is the link to the electronic supplementary material.

(PDF 420 KB)

(PDF 421 KB)

## Figures and Tables

**Fig. 8:**
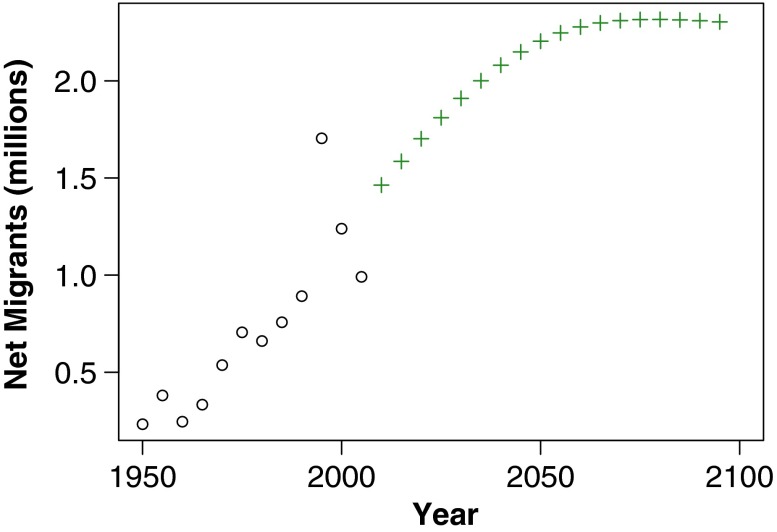
Gravity model based projections of net international migration counts for the United States

## Appendix A: Gravity Model Implementation

We implemented a version of Cohen’s (2012) gravity model, which projects net migration counts for five-year intervals starting at 2010 and ending at 2100. Projections are made for each country independently, with no redistribution step to ensure zero global net migration. For each country, projections are produced as follows. Let *L*(*t*) be the population of country *c* at time *t* (in millions) and *M*(*t*) be the population of the rest of the world at time *t* (in millions). Then expected in-migration to country *c* is given by *a*×*L*(*t*)^α^ *M*(*t*)^β^, where *a* is a country-specific proportionality constant, and the exponents α and β are constant across countries, with values estimated by Kim and Cohen (2010). Similary, expected out-migration from country *c* has the form *b*×*L*(*t*)^γ^ *M*(*t*)^δ^, where *b* is to be estimated, and γ and δ come from Kim and Cohen (2010).

The constants of proportionality *a* and *b* for each country are chosen to minimize the sum of squared deviations between estimates of net migration from the gravity model and WPP estimates of net migration (United Nations Population Division 2011) given in units of millions of net annual migrants. We use the values α=0.728, β=0.602, γ=0.373, and δ=0.948, reported by Cohen (2012). For each country, having estimated *a* and *b*, we calculate net migration projections by *a*×*L*(*t*)^α^ *M*(*t*)^β^−*b*×*L*(*t*)^γ^ *M*(*t*)^δ^, where *L*(*t*) and *M*(*t*) are now projected populations, also taken from WPP’s 2010 revision (United Nations Population Division 2011).

Our implementation appears to reproduce Cohen’ s (2012) results. Cohen reported the values of the proportionality constants, *a* and *b*, obtained for the United States, and provided a plot of the projections from his implementation of the gravity model. Using these, we are able to confirm that our results agree with those from Cohen implementation. Cohen reported *a*=3.43×10^−4^ and *b*=−8.28×10^−4^. We find very similar values of *a*=3.42×10^−4^ and *b*=−8.33×10^−4^. The slight discrepancies may come from having used only three decimal places for the values of α, β, γ, and δ in our implementation. Furthermore, Fig. 8 shows the projected net migration counts for the United States using our implementation of the gravity model. Our projections appear to be essentially the same as the gravity model projections plotted in Cohen (2012: Fig. 1).

## Appendix B: Regional Performance Tables

Here, we present tables of evaluation results split up by region. Because some countries in the Middle East have had much higher migration rates than other regions during the past several decades, we split out Western Asia^4^ from the rest of Asia.

When making predictions over a five-year time period, our model outperforms the gravity model in all regions and the persistence models in three out of six regions. Over longer horizons, our model outperforms both the gravity and persistence models for all regions except Oceania (Tables 3, 4 and 5).

**Table 3:** Five-year predictive performance of different methods: Mean absolute errors (MAE) and prediction interval coverage for our Bayesian hierarchical model, the gravity model, and the persistence models across different regions

Region	Model	MAE	80 % Cov. (%)	95 % Cov. (%)
Africa	Bayesian	1.61	94.5	100
	Gravity	3.22	—	—
	Persistence (of rates)	2.56	—	—
	Persistence (of counts)	2.16	—	—
Europe	Bayesian	1.73	85.0	90.0
	Gravity	3.39	—	—
	Persistence (of rates)	2.01	—	—
	Persistence (of counts)	2.02	—	—
Americas	Bayesian	1.38	94.9	100
	Gravity	2.58	—	—
	Persistence (of rates)	1.39	—	—
	Persistence (of counts)	1.23	—	—
Oceania	Bayesian	2.23	91.7	100
	Gravity	2.86	—	—
	Persistence (of rates)	1.82	—	—
	Persistence (of counts)	1.75	—	—
Western Asia	Bayesian	17.54	77.8	88.9
	Gravity	21.28	—	—
	Persistence (of rates)	17.27	—	—
	Persistence (of counts)	19.16	—	—
Rest of Asia	Bayesian	2.37	97.0	97.0
	Gravity	2.91	—	—
	Persistence (of rates)	2.87	—	—
	Persistence (of counts)	2.80	—	—
World	Bayesian	3.24	91.4	96.4
	Gravity	4.70	—	—
	Persistence (of rates)	3.57	—	—
	Persistence (of counts)	3.58	—	—

**Table 4:** Fifteen-year predictive performance of different methods: Mean absolute errors (MAE) and prediction interval coverage for our Bayesian hierarchical model, the gravity model, and the persistence models across different regions

Region	Model	MAE	80 % Cov. (%)	95 % Cov. (%)
Africa	Bayesian	4.60	84.8	95.2
	Gravity	7.14	—	—
	Persistence (of rates)	7.45	—	—
	Persistence (of counts)	6.38	—	—
Europe	Bayesian	3.44	78.3	87.5
	Gravity	5.87	—	—
	Persistence (of rates)	5.49	—	—
	Persistence (of counts)	5.62	—	—
Americas	Bayesian	2.44	89.7	93.2
	Gravity	3.78	—	—
	Persistence (of rates)	2.79	—	—
	Persistence (of counts)	2.57	—	—
Oceania	Bayesian	4.25	83.3	94.4
	Gravity	4.68	—	—
	Persistence (of rates)	3.53	—	—
	Persistence (of counts)	3.58	—	—
Western Asia	Bayesian	15.23	85.2	92.6
	Gravity	18.59	—	—
	Persistence (of rates)	19.46	—	—
	Persistence (of counts)	19.21	—	—
Rest of Asia	Bayesian	3.86	87.9	97.0
	Gravity	3.92	—	—
	Persistence (of rates)	5.99	—	—
	Persistence (of counts)	5.34	—	—
World	Bayesian	4.76	84.9	93.4
	Gravity	6.57	—	—
	Persistence (of rates)	6.74	—	—
	Persistence (of counts)	6.30	—	—

**Table 5:** Thirty-year predictive performance of different methods: Mean absolute errors (MAE) and prediction interval coverage for our Bayesian hierarchical model, the gravity model, and the persistence models across different regions

Region	Model	MAE	80 % Cov. (%)	95 % Cov. (%)
Africa	Bayesian	5.63	77.3	87.3
	Gravity	17.06	—	—
	Persistence (of rates)	10.40	—	—
	Persistence (of counts)	7.33	—	—
Europe	Bayesian	3.25	73.3	86.7
	Gravity	5.50	—	—
	Persistence (of rates)	3.27	—	—
	Persistence (of counts)	3.25	—	—
Americas	Bayesian	4.17	81.2	94.9
	Gravity	8.44	—	—
	Persistence (of rates)	5.29	—	—
	Persistence (of counts)	4.72	—	—
Oceania	Bayesian	5.16	79.2	94.4
	Gravity	7.53	—	—
	Persistence (of rates)	4.36	—	—
	Persistence (of counts)	4.23	—	—
Western Asia	Bayesian	11.08	76.9	88.0
	Gravity	27.17	—	—
	Persistence (of rates)	14.21	—	—
	Persistence (of counts)	11.58	—	—
Rest of Asia	Bayesian	4.42	76.3	87.9
	Gravity	10.94	—	—
	Persistence (of rates)	5.91	—	—
	Persistence (of counts)	5.17	—	—
World	Bayesian	5.12	77.2	89.3
	Gravity	12.32	—	—
	Persistence (of rates)	7.17	—	—
	Persistence (of counts)	5.82	—	—

## Footnotes

1 Strictly speaking, this is not a rate. A rate should divide counts of some event by the population exposed to risk of that event. Here, if a country is a net receiver, the real exposed population is that of the rest of the world rather than the population of country *c*, so our “rate” doesn’t have the correct exposed population in the denominator. Nevertheless, we follow convention and continue to call this a net migration rate, even though the terminology is controversial. This convention is fairly widely used, including in the WPP.

2 Other sensible choices of prior yield very similar results. For example, fixing λ = 0 and taking ${{\upsigma }^{2}_{c}} \sim IG(0.001, 0.001)$ both produce only small changes in predictions. We incorporate an extra level of hyperpriors in part to encourage more shrinkage of parameter values toward a global mean. Additionally, more informative priors would be possible if one wished to incorporate knowledge from other sources, such as region-specific knowledge of means or variances in migration.

3 This figure is robust to incorporating a threshold. If we require that absolute migration rate be at least 1 net annual migrant per thousand either before or after the change, the figure is still 46 %.
