# Supplementary material for: Bayesian Probabilistic Projection of International Migration
Source: Demography. 2015 Sep 10;52(5):1627–50. doi: 10.1007/s13524-015-0415-0 (PMC4605963; doi:10.1007/s13524-015-0415-0)
Supplement: Supplementary file 1 — (PDF 420 KB) [file 13524_2015_415_MOESM1_ESM.pdf]

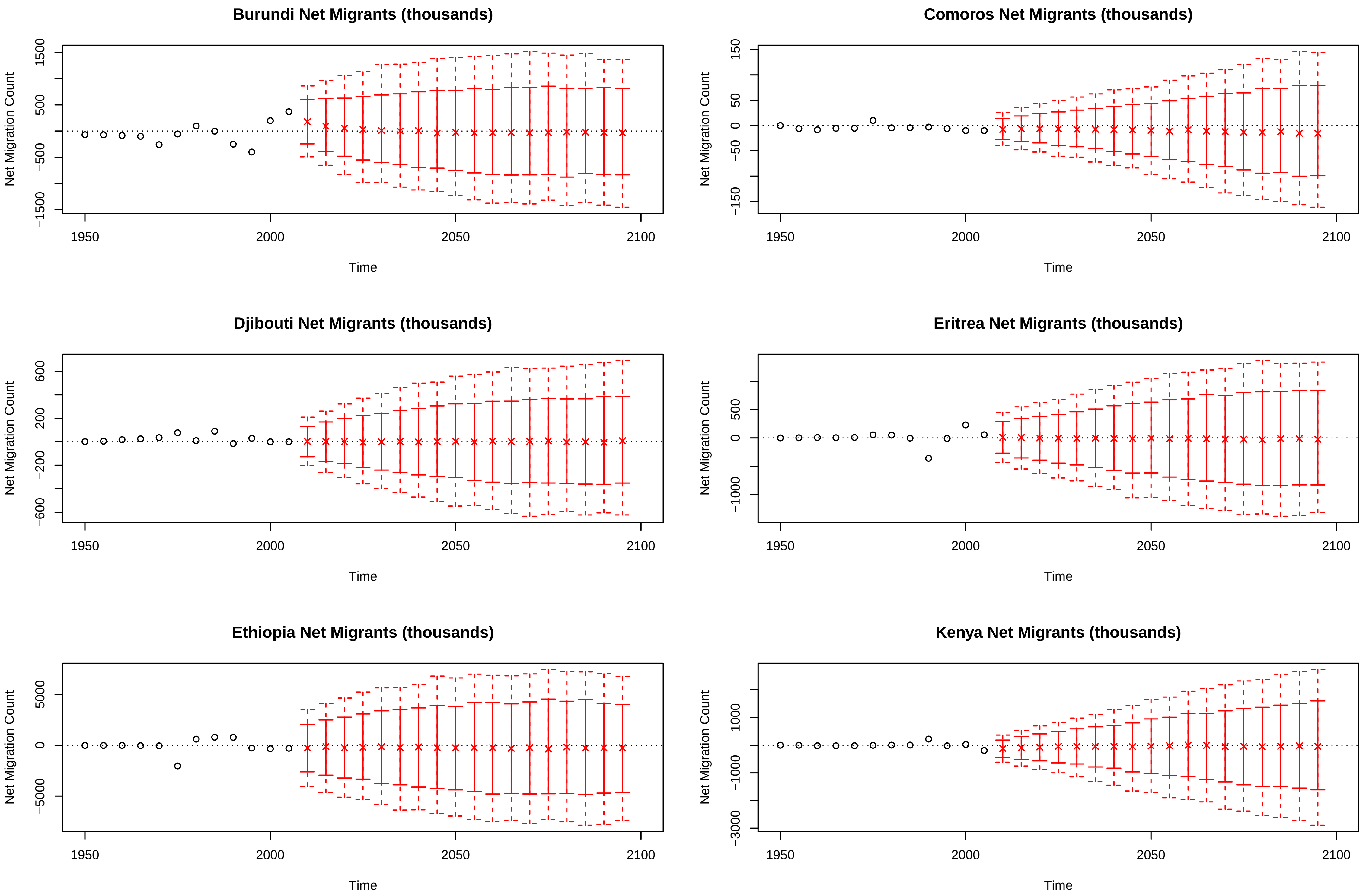
Burundi Net Migrants (thousands)
Comoros Net Migrants (thousands)
Djibouti Net Migrants (thousands)
Eritrea Net Migrants (thousands)
Ethiopia Net Migrants (thousands)
Kenya Net Migrants (thousands)
Net Migration Count
Time
1950
2000
2050
2100
−1500
−500
500
1500
−150
−50
0
50
150
−600
−200
200
600
−1000
0
500
−5000
0
5000
−3000
−1000
1000

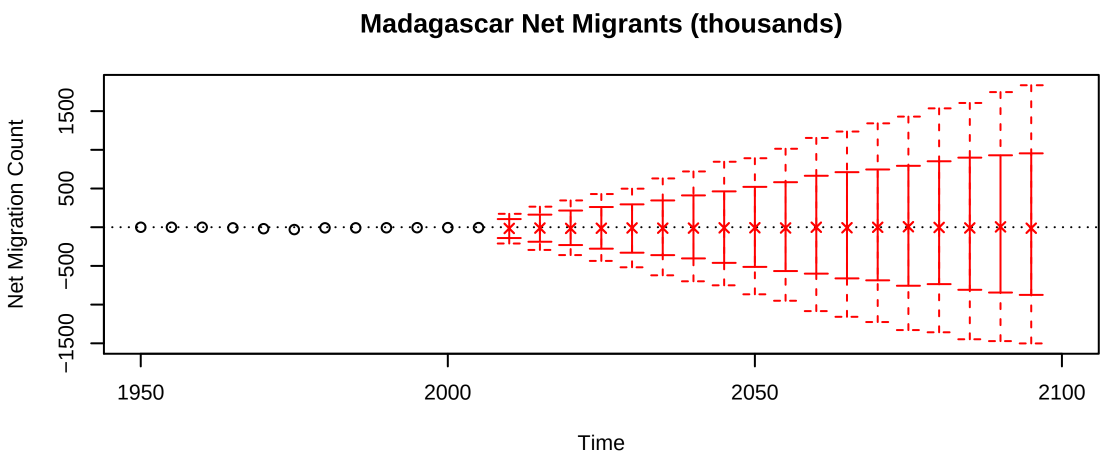
Madagascar Net Migrants (thousands)
Net Migration Count
−1500
−500
500
1500
1950
2000
2050
2100
Time

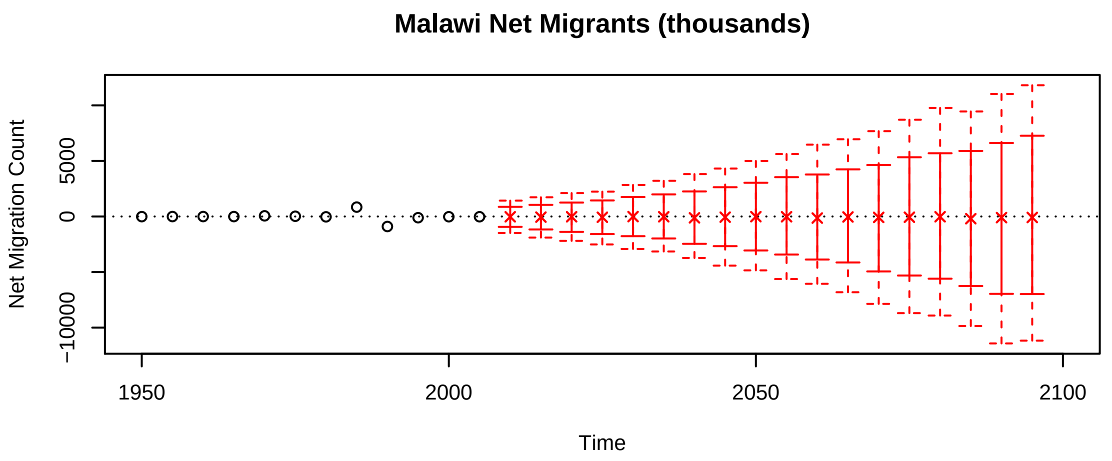
Malawi Net Migrants (thousands)
Net Migration Count
−10000
0
5000
1950
2000
2050
2100
Time

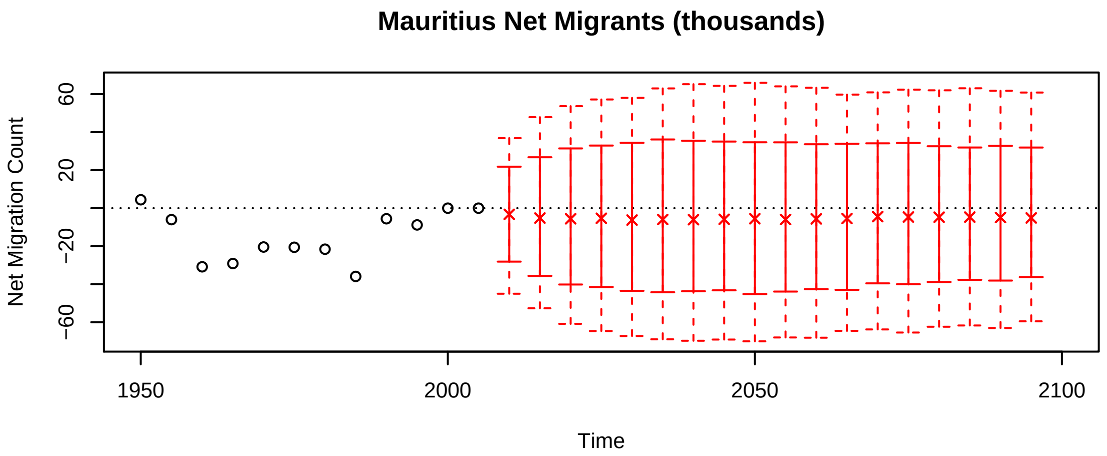
Mauritius Net Migrants (thousands)
Net Migration Count
−60
−20
20
60
1950
2000
2050
2100
Time

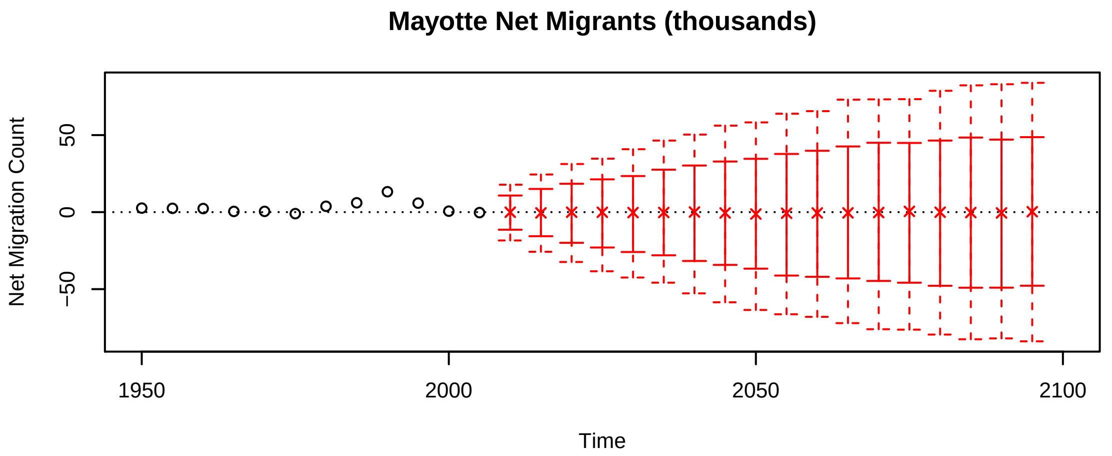
Mayotte Net Migrants (thousands)
Net Migration Count
−50
0
50
1950
2000
2050
2100
Time

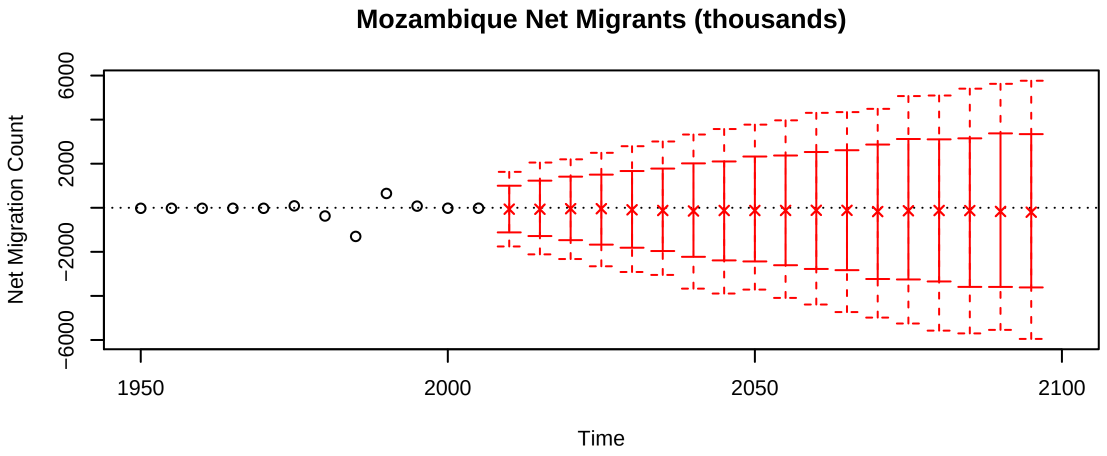
Mozambique Net Migrants (thousands)
Net Migration Count
−6000
−2000
2000
6000
1950
2000
2050
2100
Time

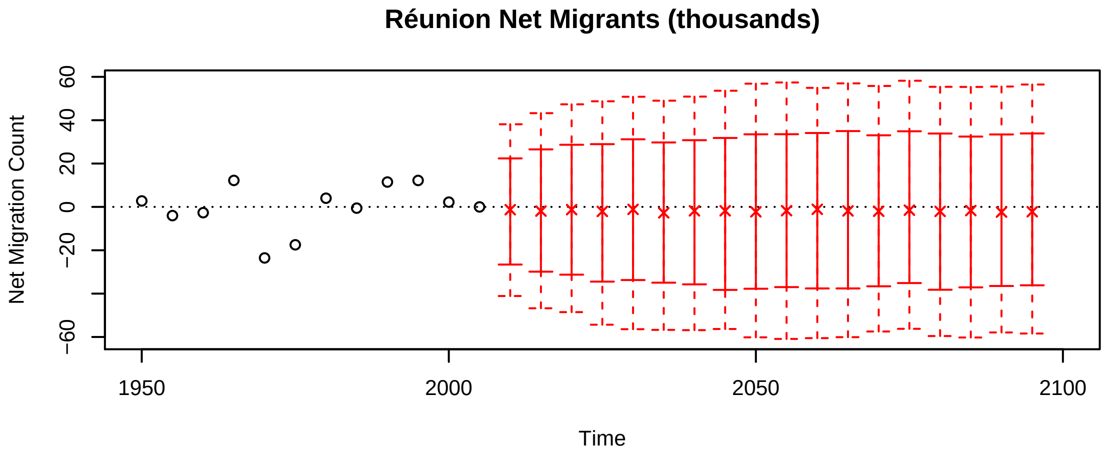
Réunion Net Migrants (thousands)
Net Migration Count
−60
−20
0
20
40
60
1950
2000
2050
2100
Time

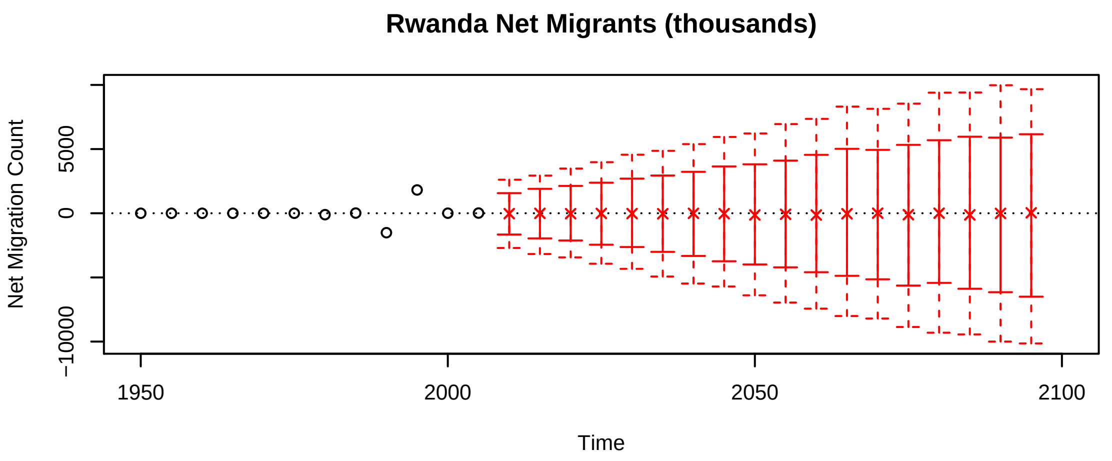
Rwanda Net Migrants (thousands)
Net Migration Count
−10000
0
5000
1950
2000
2050
2100
Time

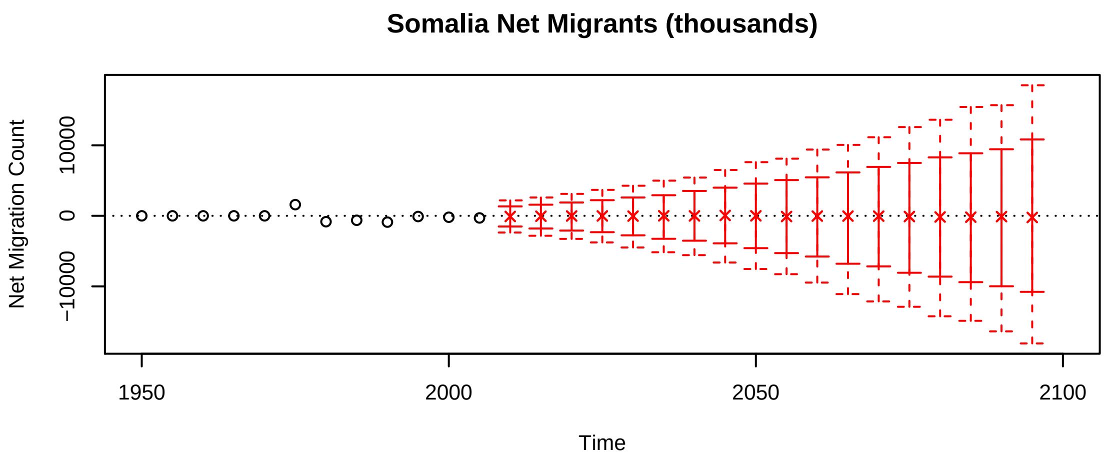
Somalia Net Migrants (thousands)
Net Migration Count
−10000
0
10000
1950
2000
2050
2100
Time

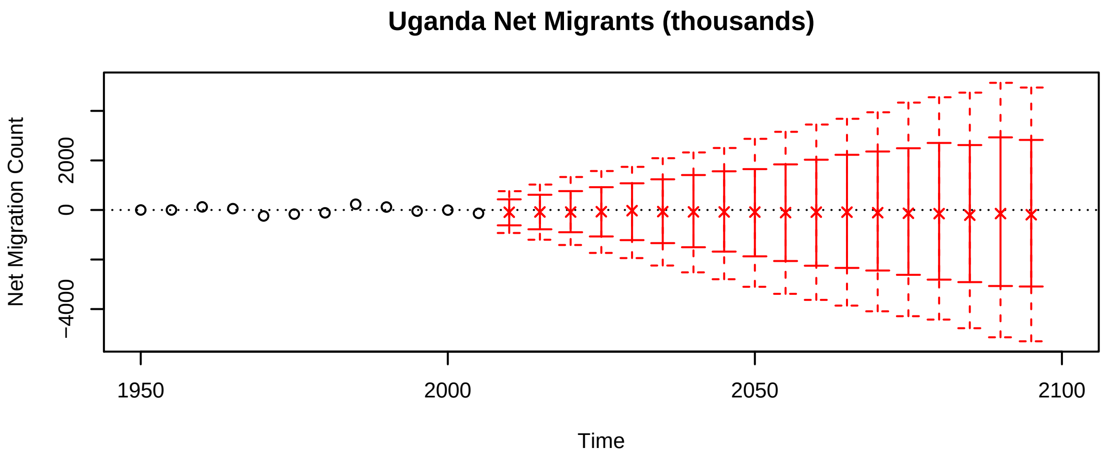
Uganda Net Migrants (thousands)
Net Migration Count
−4000
0
2000
1950
2000
2050
2100
Time

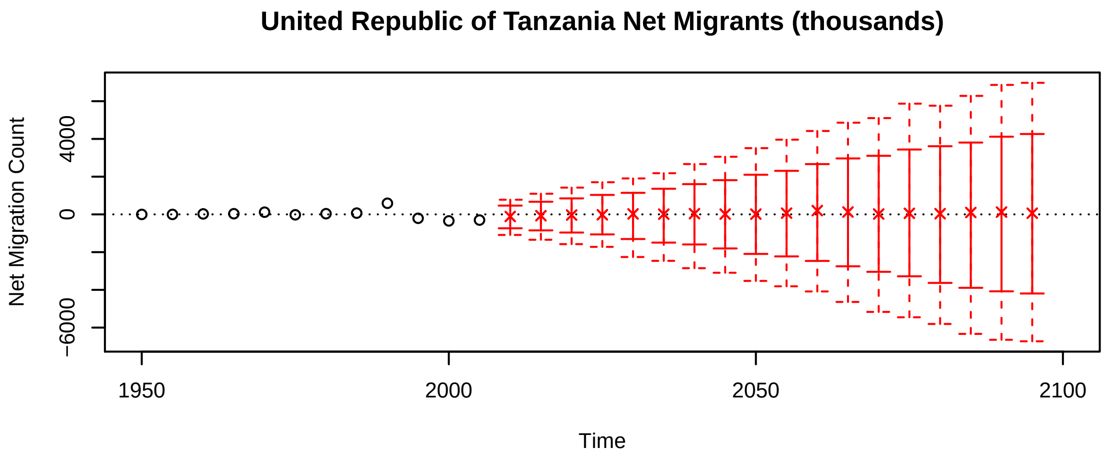
United Republic of Tanzania Net Migrants (thousands)
Net Migration Count
−6000
0
4000
1950
2000
2050
2100
Time

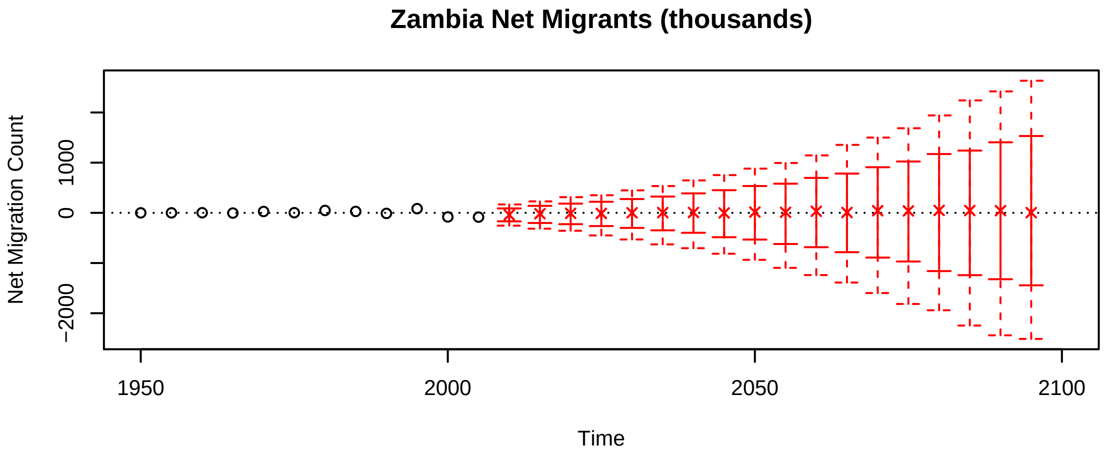
Zambia Net Migrants (thousands)
Net Migration Count
−2000
0
1000
1950
2000
2050
2100
Time

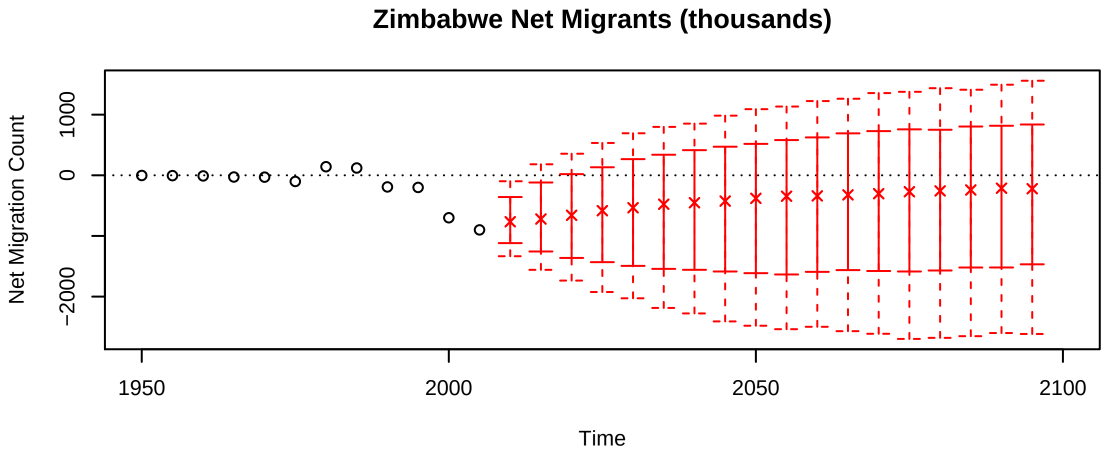
Zimbabwe Net Migrants (thousands)
Net Migration Count
−2000
0
1000
1950
2000
2050
2100
Time

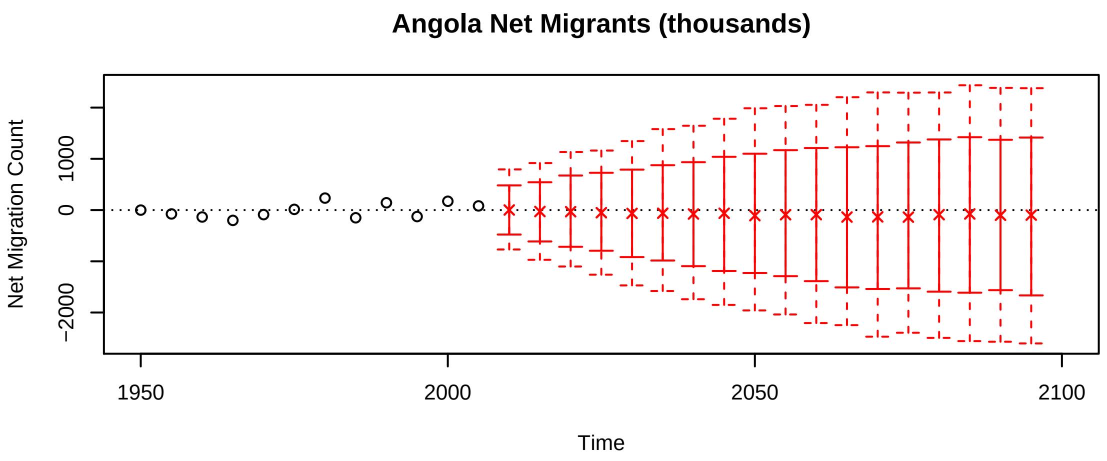
Angola Net Migrants (thousands)
Net Migration Count
−2000
0
1000
1950
2000
2050
2100
Time

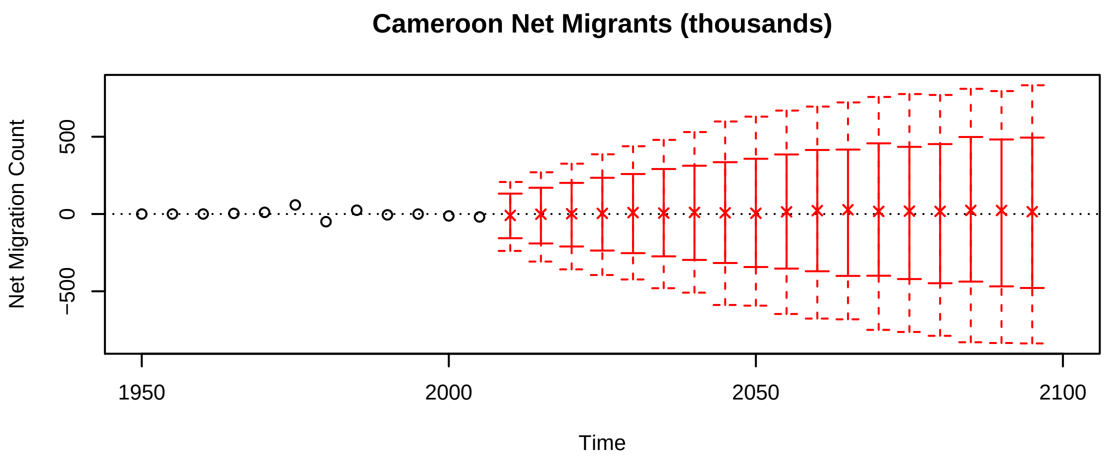
Cameroon Net Migrants (thousands)
Net Migration Count
−500
0
500
1950
2000
2050
2100
Time

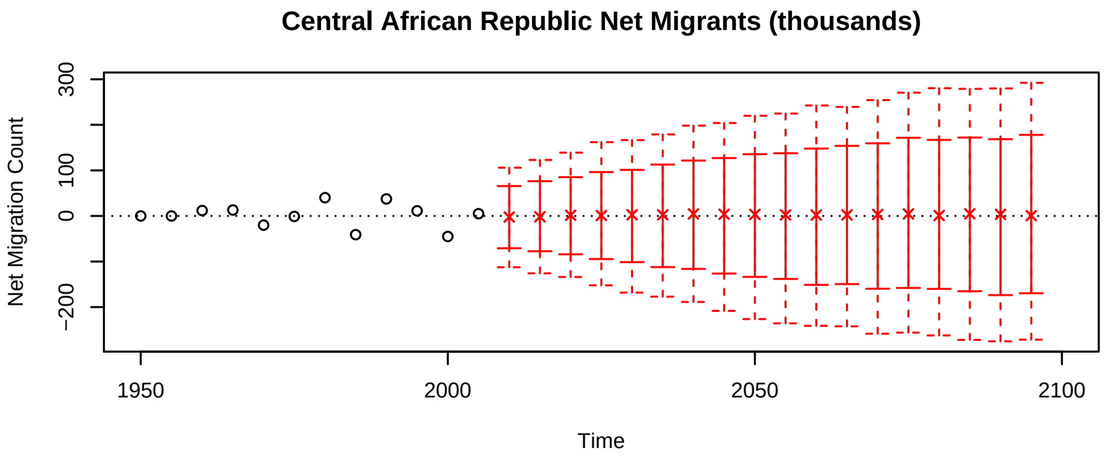
Central African Republic Net Migrants (thousands)
Net Migration Count
−200
0
100
300
1950
2000
2050
2100
Time

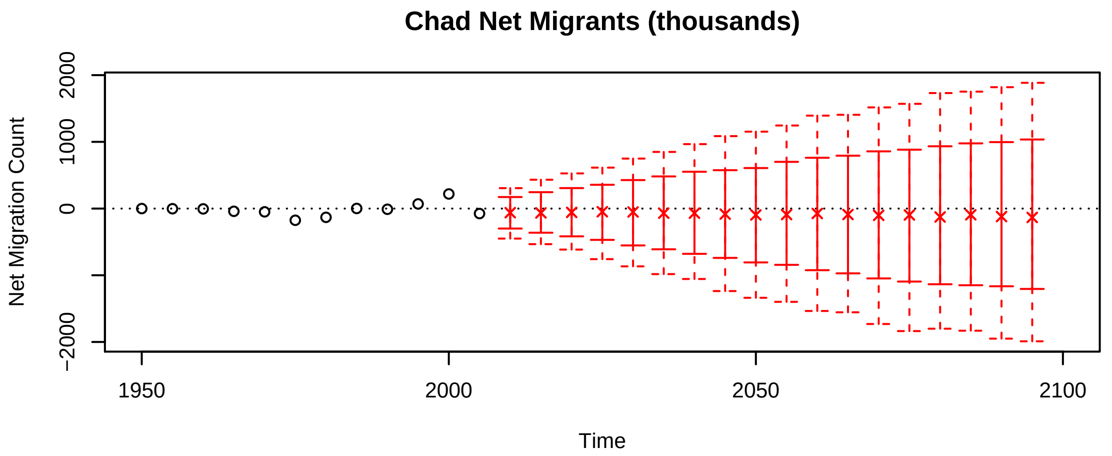
Chad Net Migrants (thousands)
Net Migration Count
−2000
0
1000
2000
1950
2000
2050
2100
Time

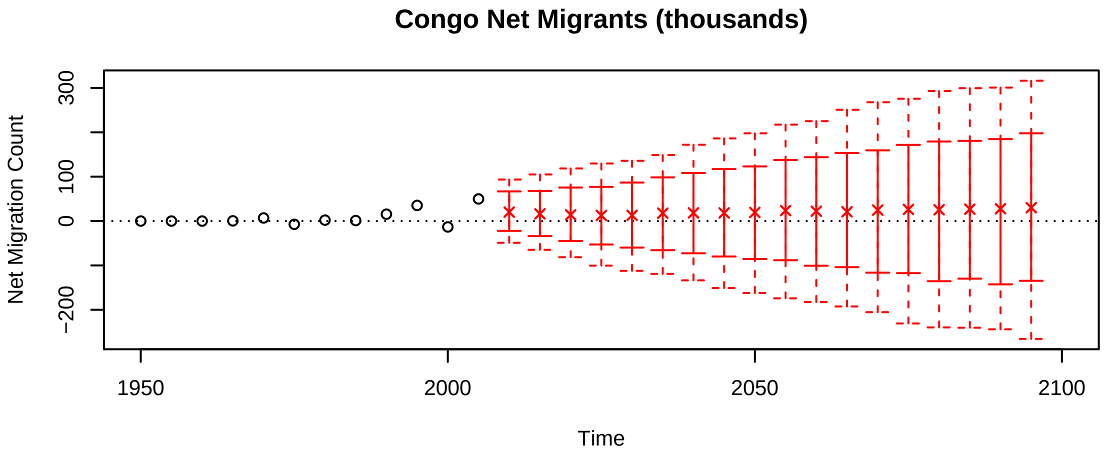
Congo Net Migrants (thousands)
Net Migration Count
−200
0
100
300
1950
2000
2050
2100
Time

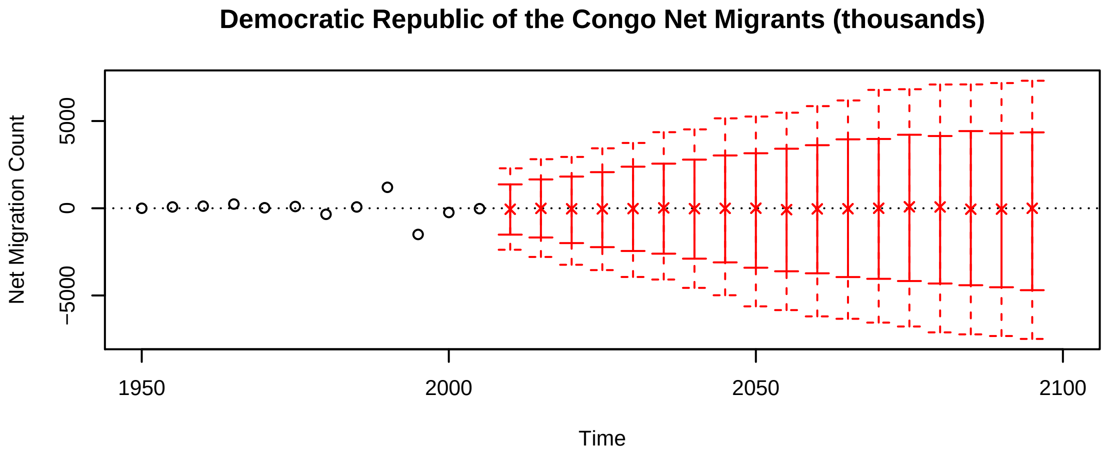
Democratic Republic of the Congo Net Migrants (thousands)
Net Migration Count
−5000
0
5000
1950
2000
2050
2100
Time

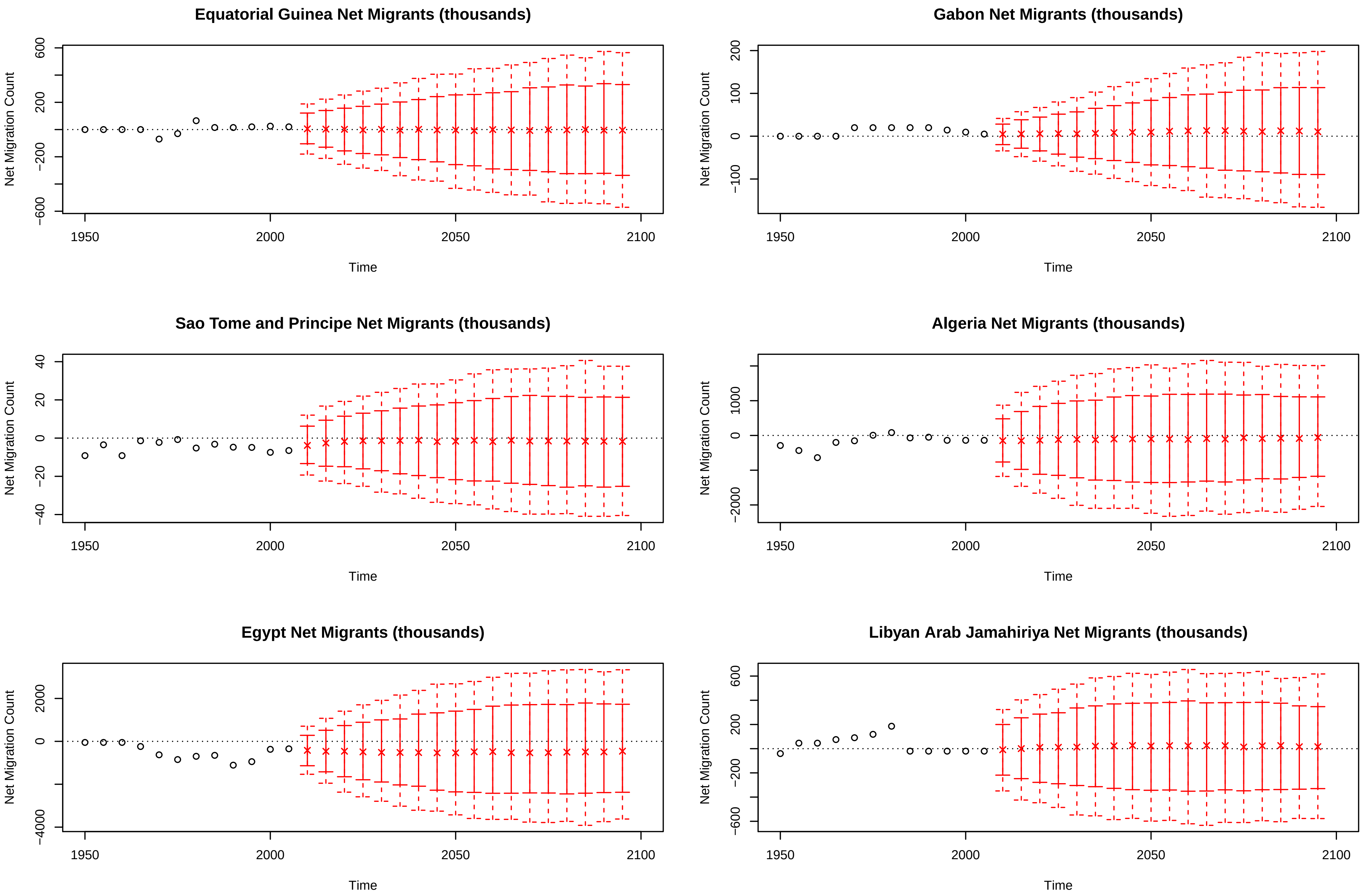
Equatorial Guinea Net Migrants (thousands)
Net Migration Count
−600
−200
200
600
1950
2000
2050
2100
Time
Gabon Net Migrants (thousands)
Net Migration Count
−100
0
100
200
1950
2000
2050
2100
Time
Sao Tome and Principe Net Migrants (thousands)
Net Migration Count
−40
−20
0
20
40
1950
2000
2050
2100
Time
Algeria Net Migrants (thousands)
Net Migration Count
−2000
0
1000
1950
2000
2050
2100
Time
Egypt Net Migrants (thousands)
Net Migration Count
−4000
0
2000
1950
2000
2050
2100
Time
Libyan Arab Jamahiriya Net Migrants (thousands)
Net Migration Count
−600
−200
200
600
1950
2000
2050
2100
Time

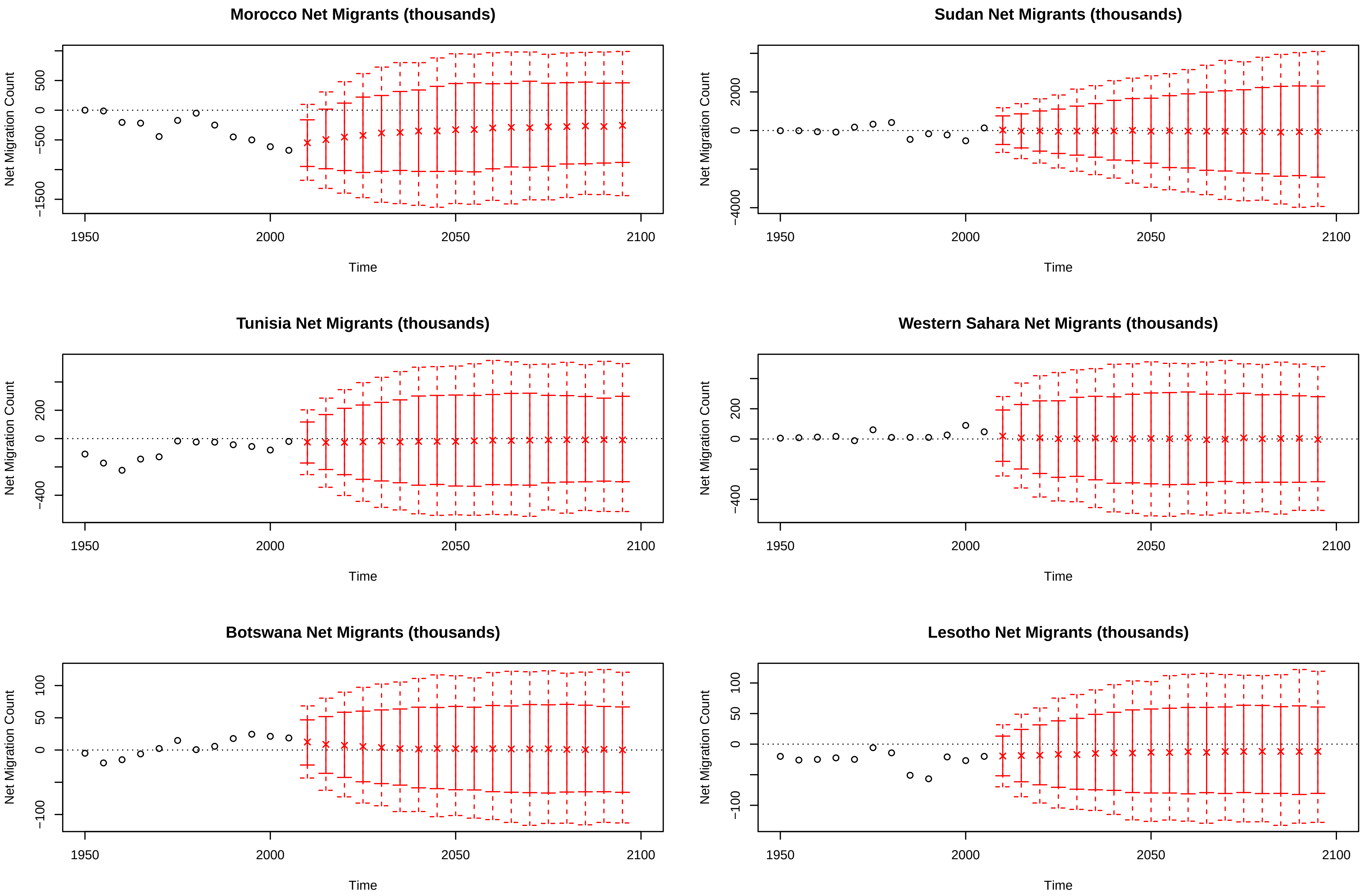

Morocco Net Migrants (thousands)
Sudan Net Migrants (thousands)
Tunisia Net Migrants (thousands)
Western Sahara Net Migrants (thousands)
Botswana Net Migrants (thousands)
Lesotho Net Migrants (thousands)
Net Migration Count
Time
1950
2000
2050
2100
500
0
−500
−1500
2000
−4000
200
−400
100
50
−100

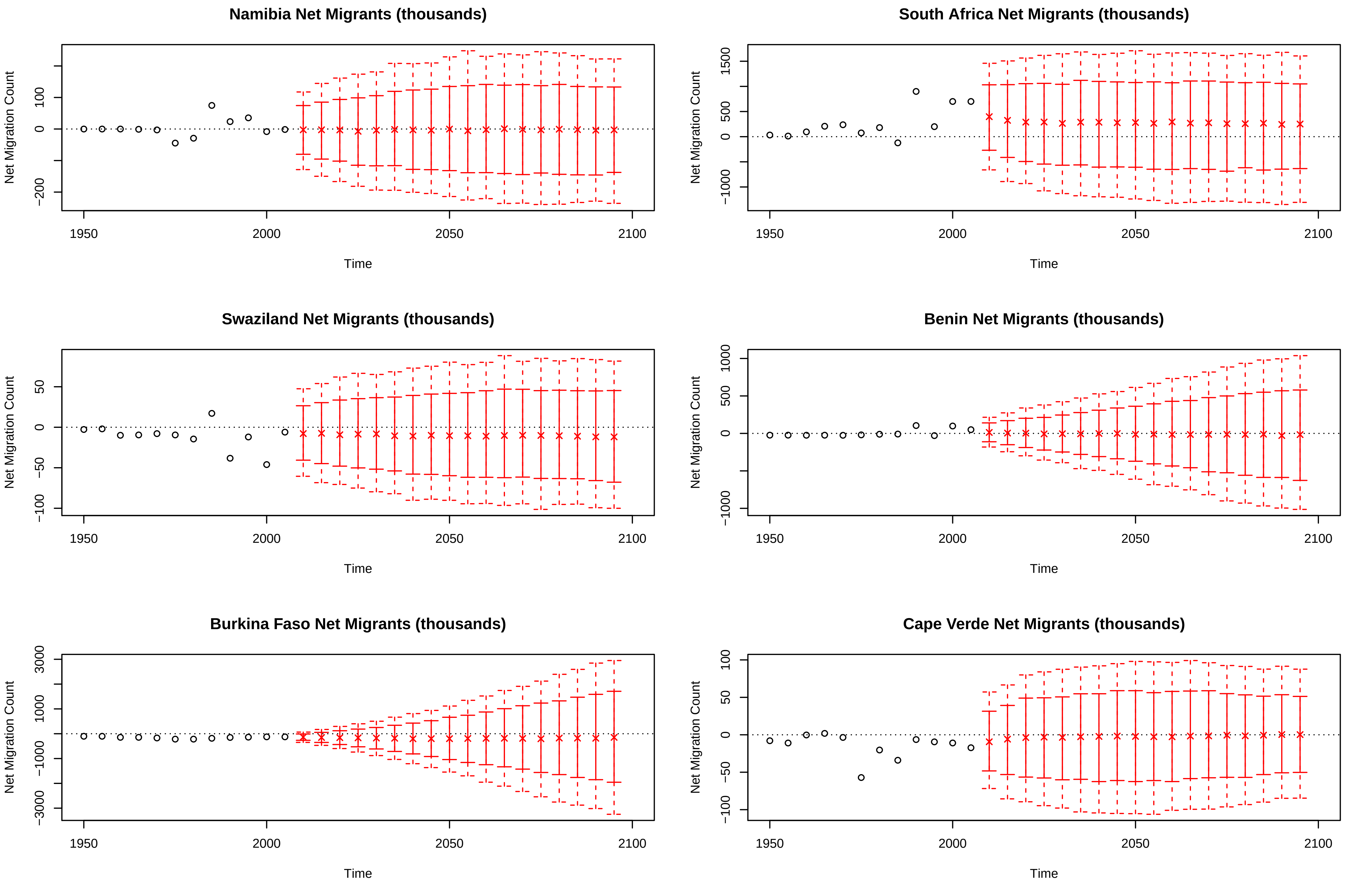
Namibia Net Migrants (thousands)
South Africa Net Migrants (thousands)
Swaziland Net Migrants (thousands)
Benin Net Migrants (thousands)
Burkina Faso Net Migrants (thousands)
Cape Verde Net Migrants (thousands)
Net Migration Count
Time
1950
2000
2050
2100
−200
0
100
−1000
0
500
1500
−100
−50
0
50
−1000
0
500
1000
−3000
−1000
1000
3000
−100
−50
0
50
100

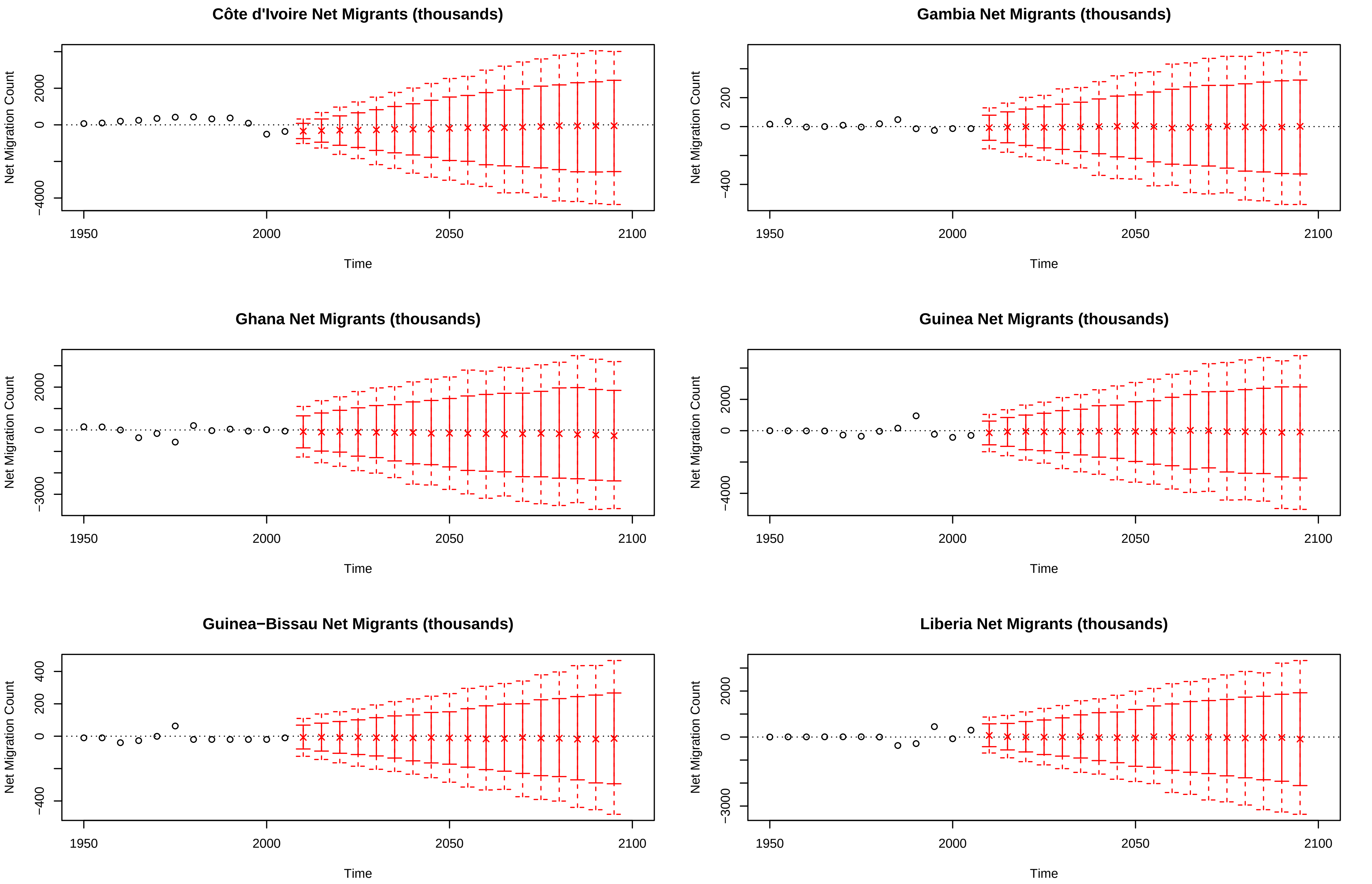

Côte d'Ivoire Net Migrants (thousands)
Net Migration Count
−4000
0
2000
1950
2000
2050
2100
Time
Gambia Net Migrants (thousands)
Net Migration Count
−400
0
200
1950
2000
2050
2100
Time
Ghana Net Migrants (thousands)
Net Migration Count
−3000
0
2000
1950
2000
2050
2100
Time
Guinea Net Migrants (thousands)
Net Migration Count
−4000
0
2000
1950
2000
2050
2100
Time
Guinea−Bissau Net Migrants (thousands)
Net Migration Count
−400
0
200
400
1950
2000
2050
2100
Time
Liberia Net Migrants (thousands)
Net Migration Count
−3000
0
2000
1950
2000
2050
2100
Time

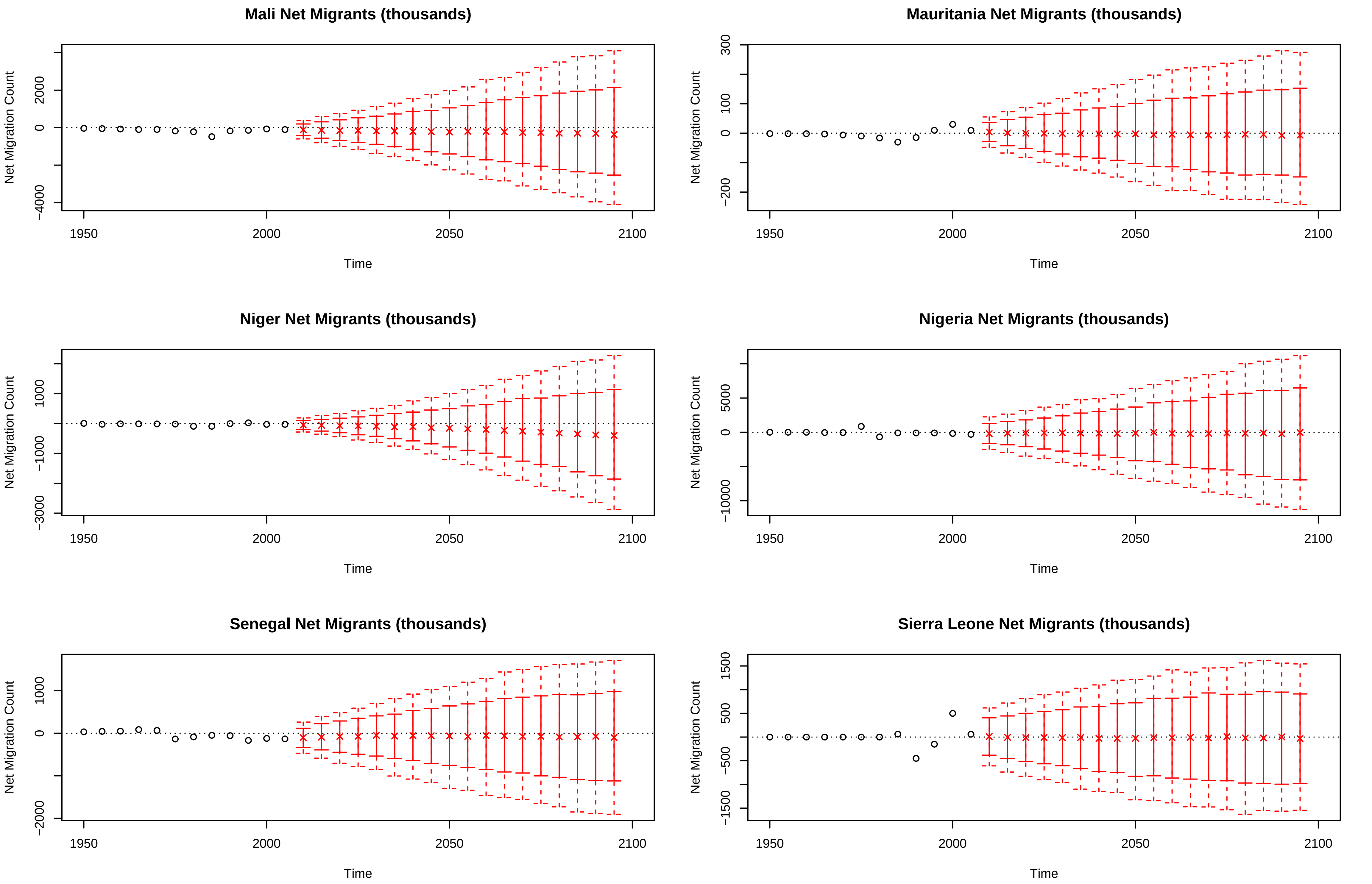
Mali Net Migrants (thousands)
Mauritania Net Migrants (thousands)
Niger Net Migrants (thousands)
Nigeria Net Migrants (thousands)
Senegal Net Migrants (thousands)
Sierra Leone Net Migrants (thousands)
Net Migration Count
Time
1950
2000
2050
2100
−4000
0
2000
−200
100
300
−3000
−1000
1000
−10000
5000
−2000
−1500
−500
500
1500

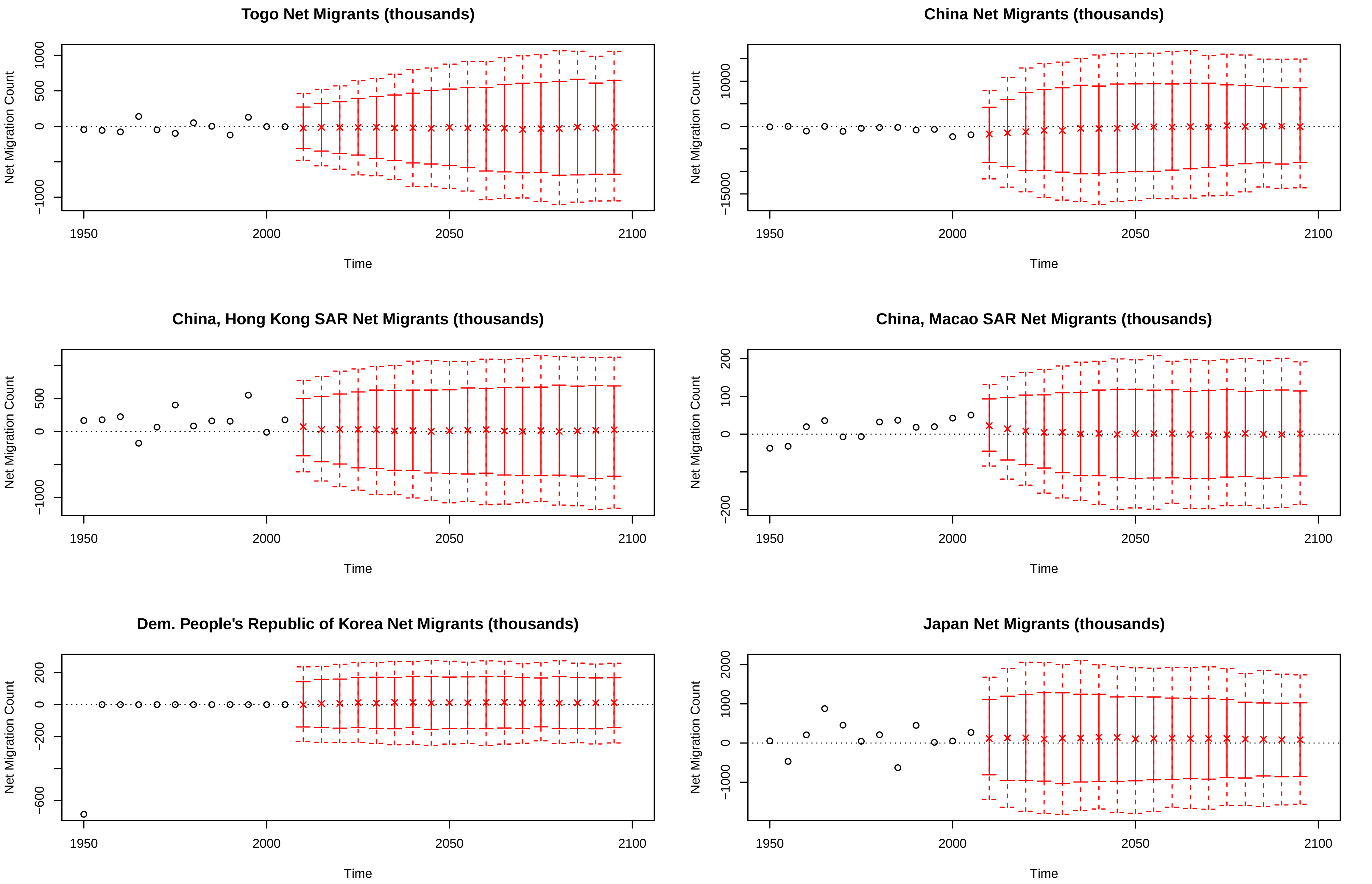
Togo Net Migrants (thousands)
China Net Migrants (thousands)
China, Hong Kong SAR Net Migrants (thousands)
China, Macao SAR Net Migrants (thousands)
Dem. People's Republic of Korea Net Migrants (thousands)
Japan Net Migrants (thousands)
Net Migration Count
Time
1950
2000
2050
2100

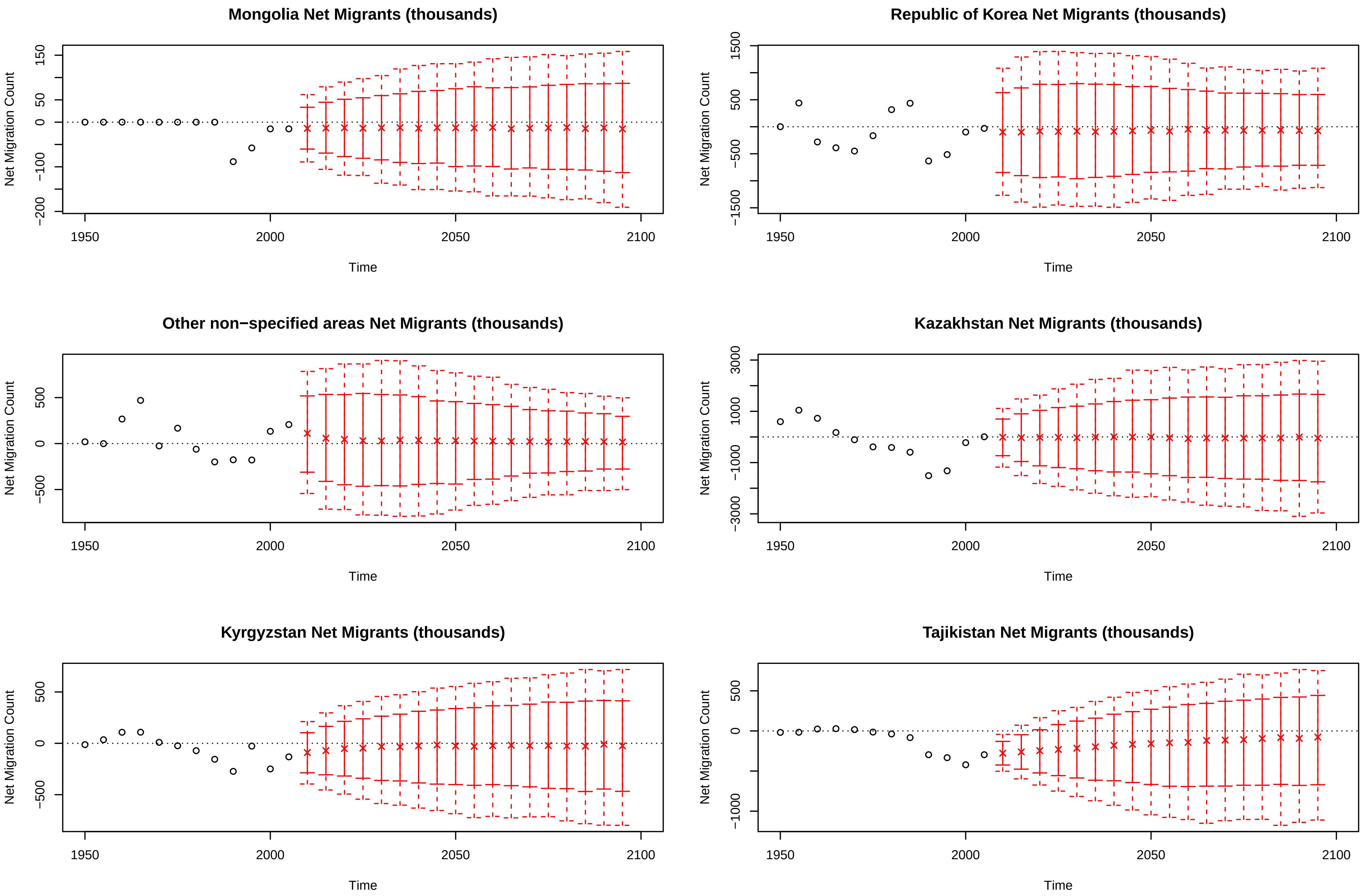
Mongolia Net Migrants (thousands)
Net Migration Count
150
50
0
−100
−200
Republic of Korea Net Migrants (thousands)
1500
500
−500
−1500
Other non−specified areas Net Migrants (thousands)
500
0
−500
Kazakhstan Net Migrants (thousands)
3000
1000
−1000
−3000
Kyrgyzstan Net Migrants (thousands)
500
0
−500
Tajikistan Net Migrants (thousands)
500
0
−1000
1950
2000
2050
2100
Time

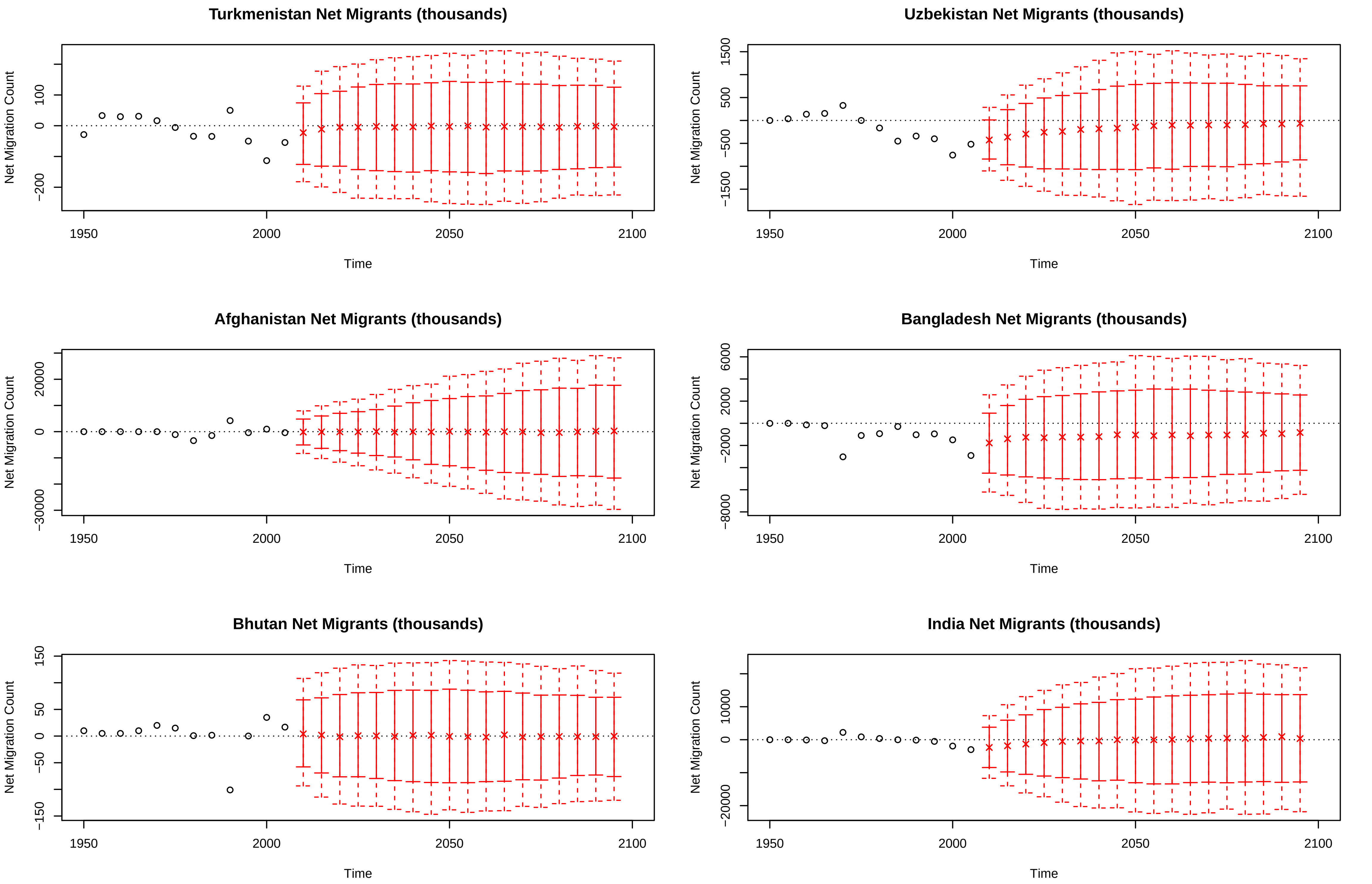
Turkmenistan Net Migrants (thousands)
Net Migration Count
-200
0
100
Time
1950
2000
2050
2100
Uzbekistan Net Migrants (thousands)
Net Migration Count
-1500
-500
500
1500
Time
1950
2000
2050
2100
Afghanistan Net Migrants (thousands)
Net Migration Count
-30000
0
20000
Time
1950
2000
2050
2100
Bangladesh Net Migrants (thousands)
Net Migration Count
-8000
-2000
2000
6000
Time
1950
2000
2050
2100
Bhutan Net Migrants (thousands)
Net Migration Count
-150
-50
0
50
150
Time
1950
2000
2050
2100
India Net Migrants (thousands)
Net Migration Count
-20000
0
10000
Time
1950
2000
2050
2100

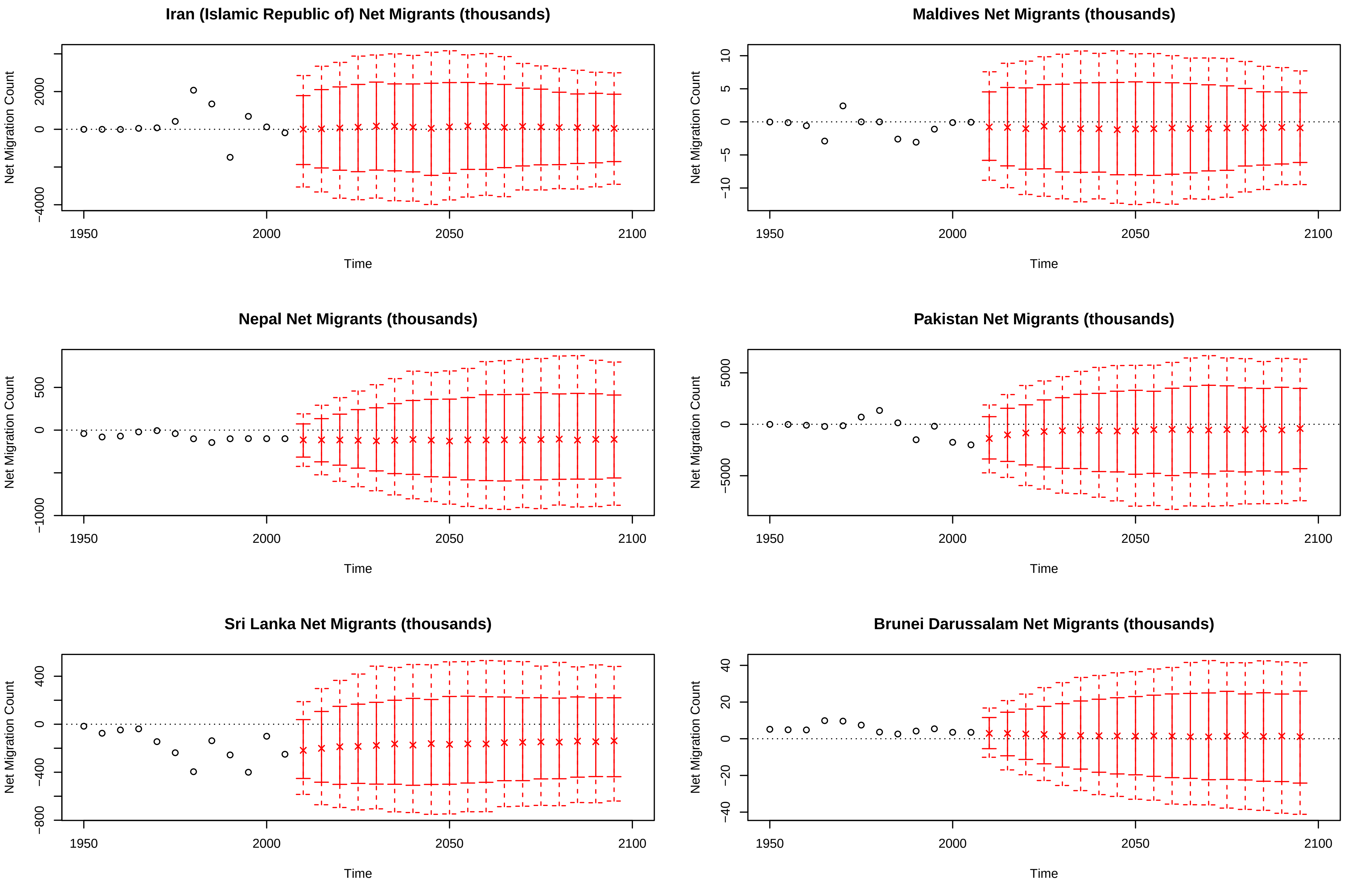

Iran (Islamic Republic of) Net Migrants (thousands)
Net Migration Count
−4000
0
2000
1950
2000
2050
2100
Time
Maldives Net Migrants (thousands)
Net Migration Count
−10
−5
0
5
10
1950
2000
2050
2100
Time
Nepal Net Migrants (thousands)
Net Migration Count
−1000
0
500
1950
2000
2050
2100
Time
Pakistan Net Migrants (thousands)
Net Migration Count
−5000
0
5000
1950
2000
2050
2100
Time
Sri Lanka Net Migrants (thousands)
Net Migration Count
−800
−400
0
400
1950
2000
2050
2100
Time
Brunei Darussalam Net Migrants (thousands)
Net Migration Count
−40
−20
0
20
40
1950
2000
2050
2100
Time

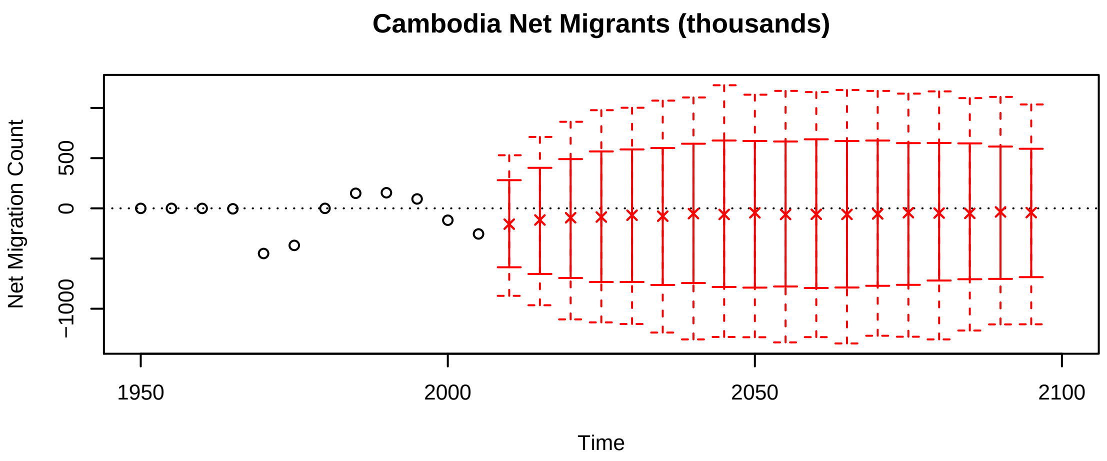
Cambodia Net Migrants (thousands)
Net Migration Count
500
0
−1000
1950
2000
2050
2100
Time

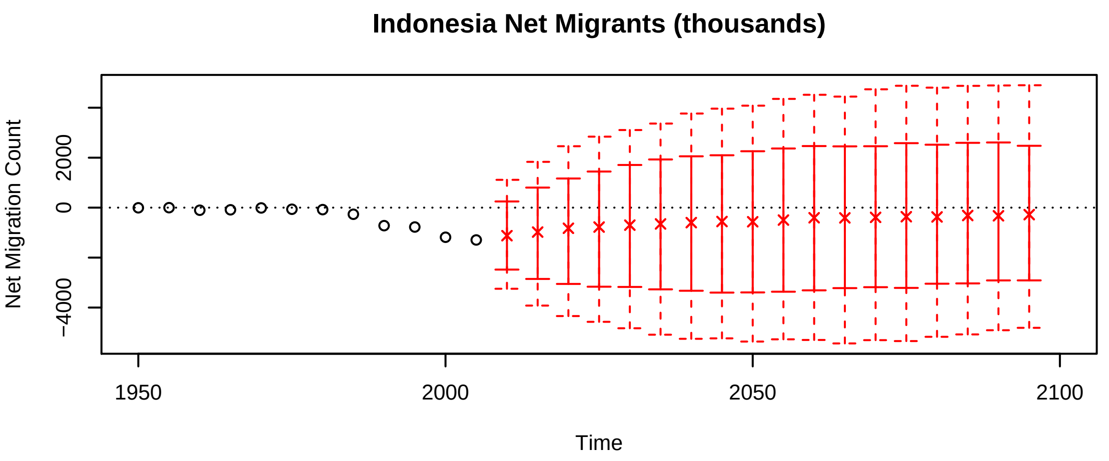
Indonesia Net Migrants (thousands)
Net Migration Count
2000
0
−4000
1950
2000
2050
2100
Time

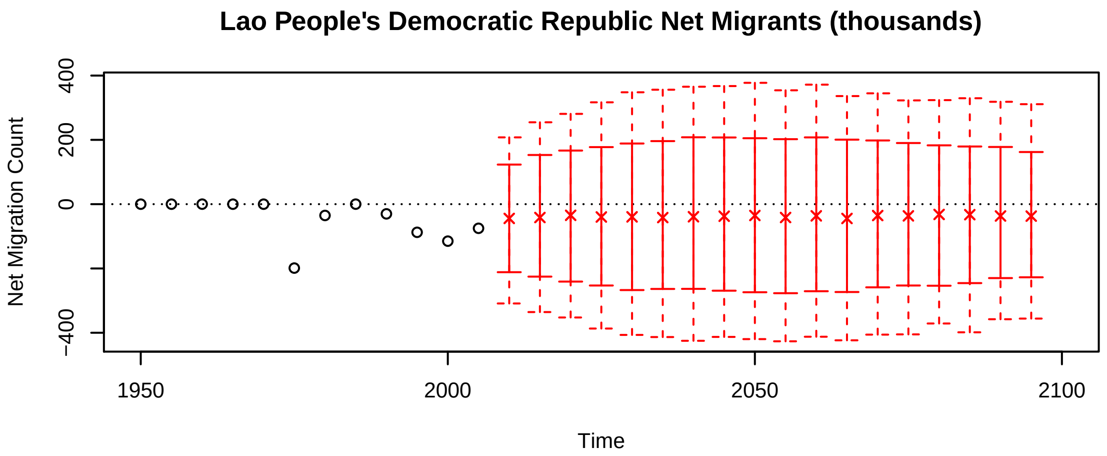
Lao People's Democratic Republic Net Migrants (thousands)
Net Migration Count
400
200
0
−400
1950
2000
2050
2100
Time

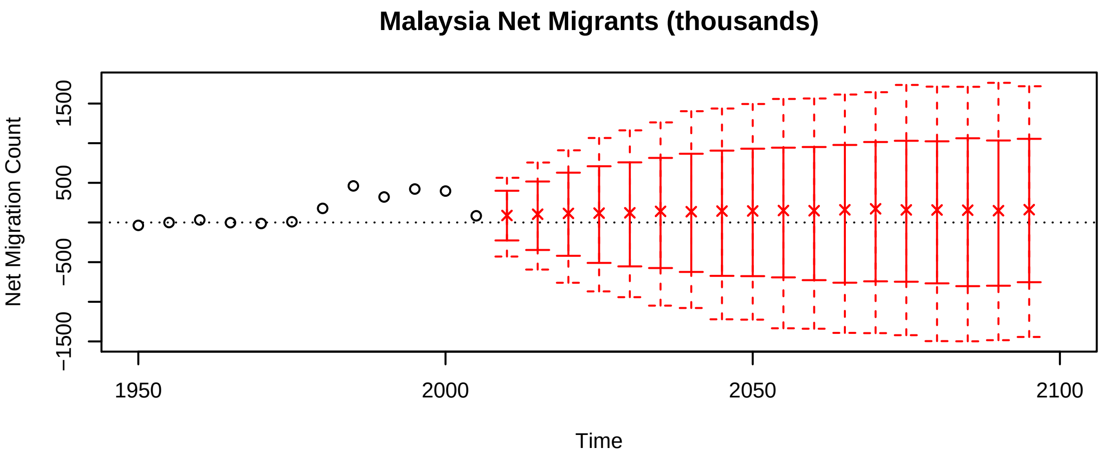
Malaysia Net Migrants (thousands)
Net Migration Count
1500
500
−500
−1500
1950
2000
2050
2100
Time

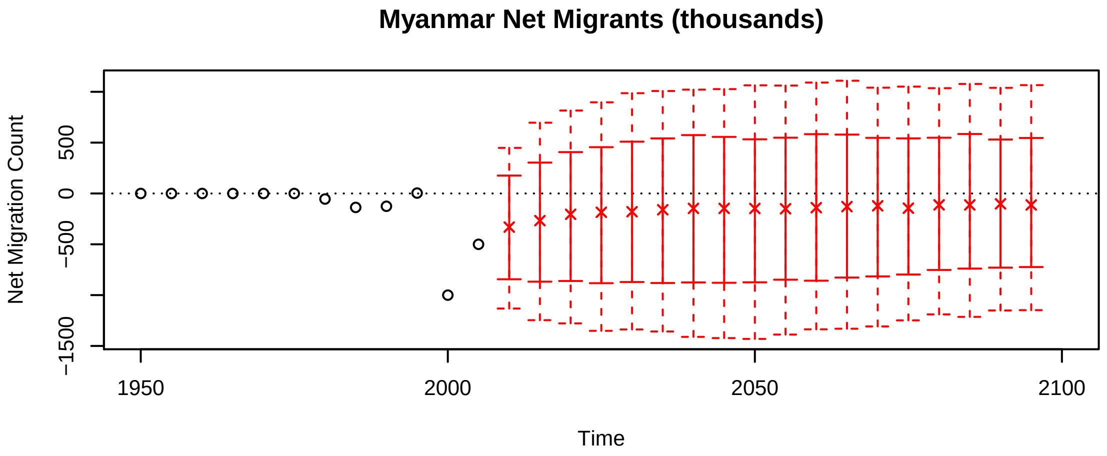
Myanmar Net Migrants (thousands)
Net Migration Count
500
0
−500
−1500
1950
2000
2050
2100
Time

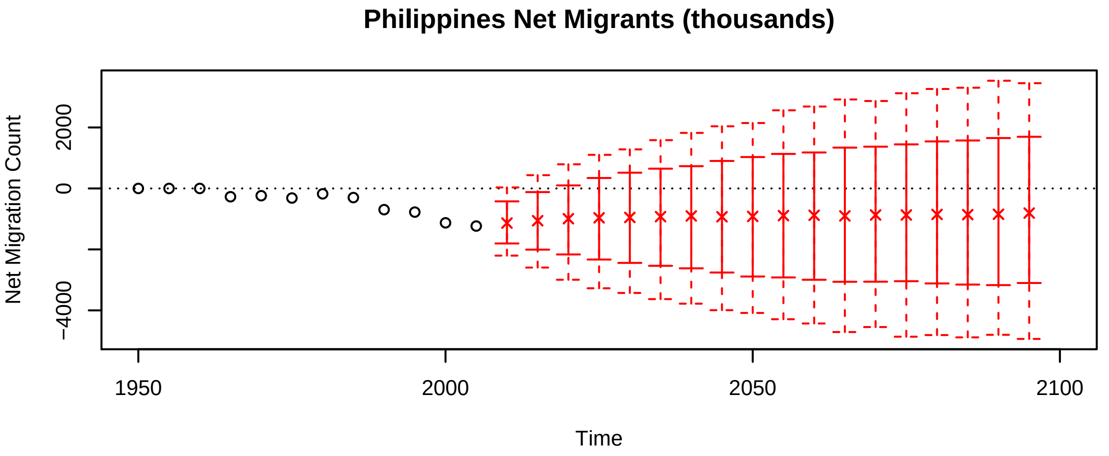
Philippines Net Migrants (thousands)
Net Migration Count
2000
0
−4000
1950
2000
2050
2100
Time

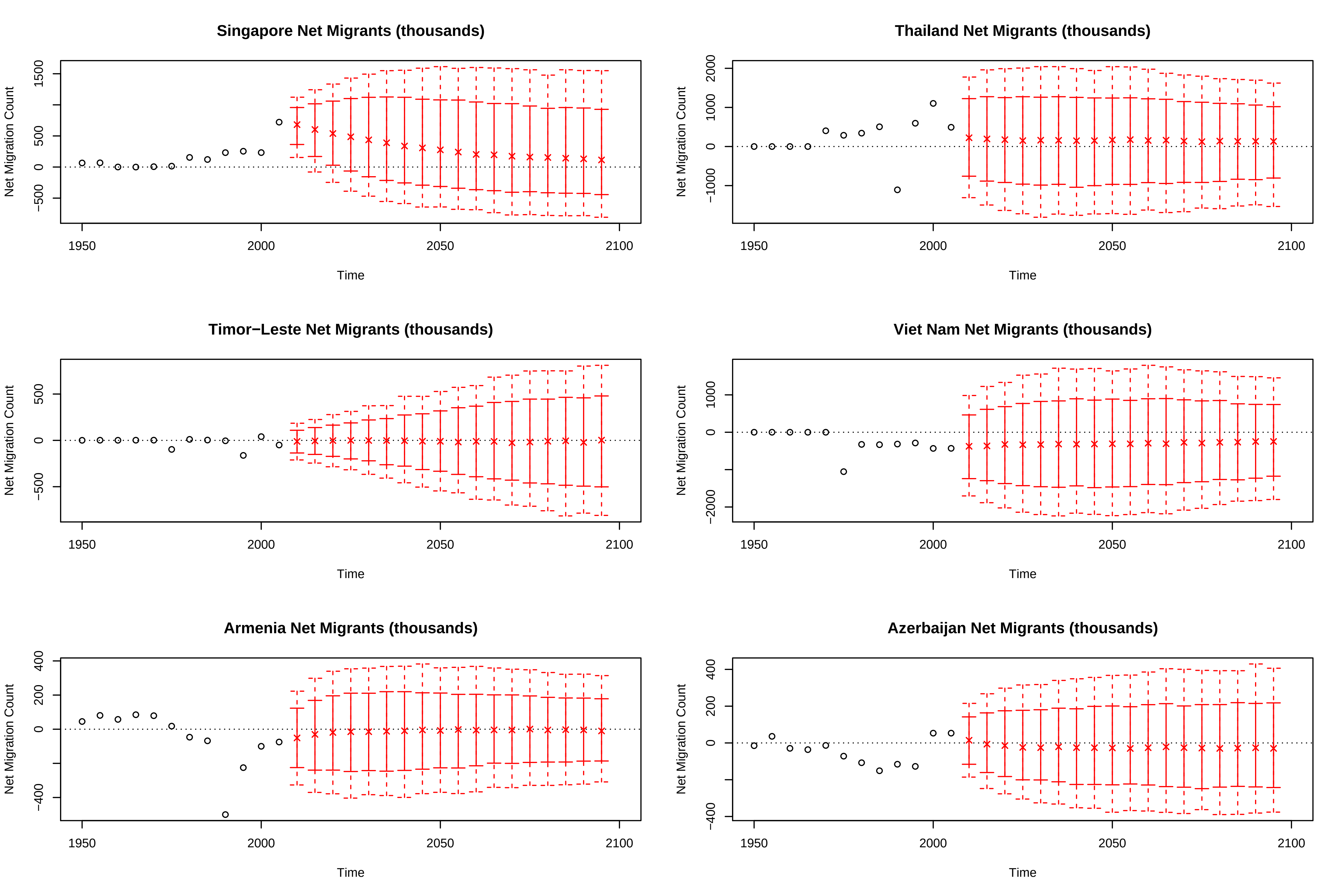
Singapore Net Migrants (thousands)
Thailand Net Migrants (thousands)
Timor−Leste Net Migrants (thousands)
Viet Nam Net Migrants (thousands)
Armenia Net Migrants (thousands)
Azerbaijan Net Migrants (thousands)
Net Migration Count
Time
1950
2000
2050
2100
−500
0
500
1500
−1000
1000
2000
−2000
−400
200
400

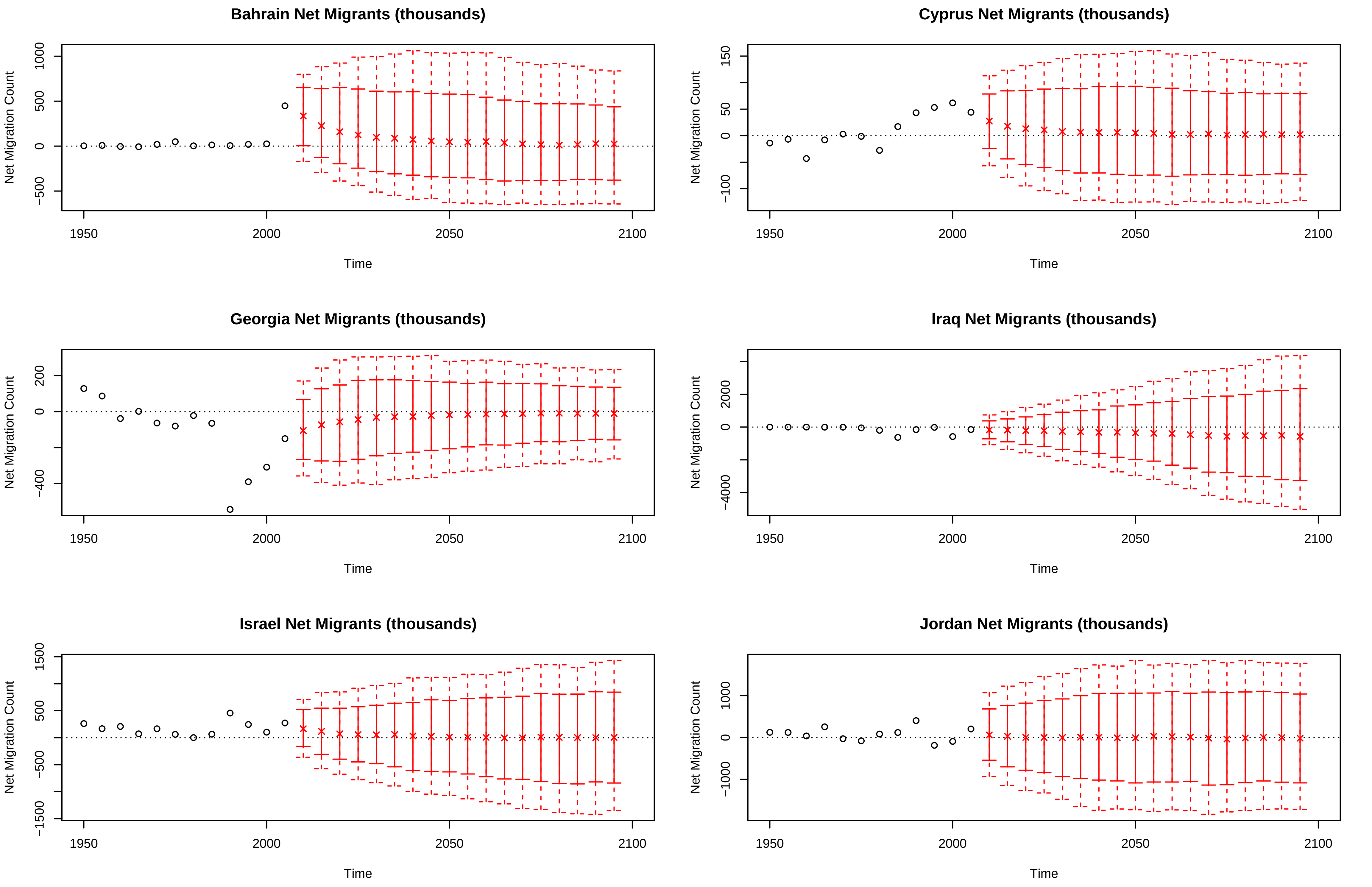
Bahrain Net Migrants (thousands)
Net Migration Count
−500
0
500
1000
Cyprus Net Migrants (thousands)
−100
−50
0
50
100
150
Georgia Net Migrants (thousands)
−400
−200
0
200
Iraq Net Migrants (thousands)
−4000
−2000
0
2000
Israel Net Migrants (thousands)
−1500
−1000
−500
0
500
1000
1500
Jordan Net Migrants (thousands)
−1000
0
1000
1950
2000
2050
2100
Time

Kuwait Net Migrants (thousands)

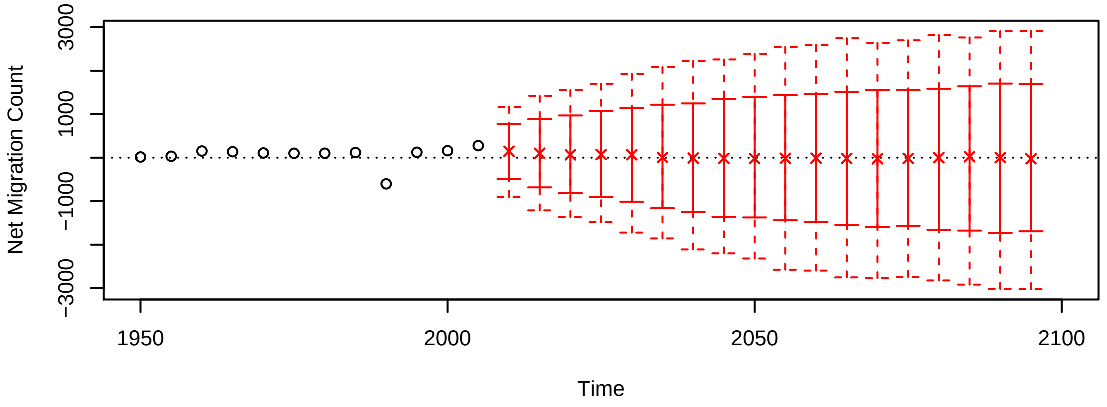

Lebanon Net Migrants (thousands)

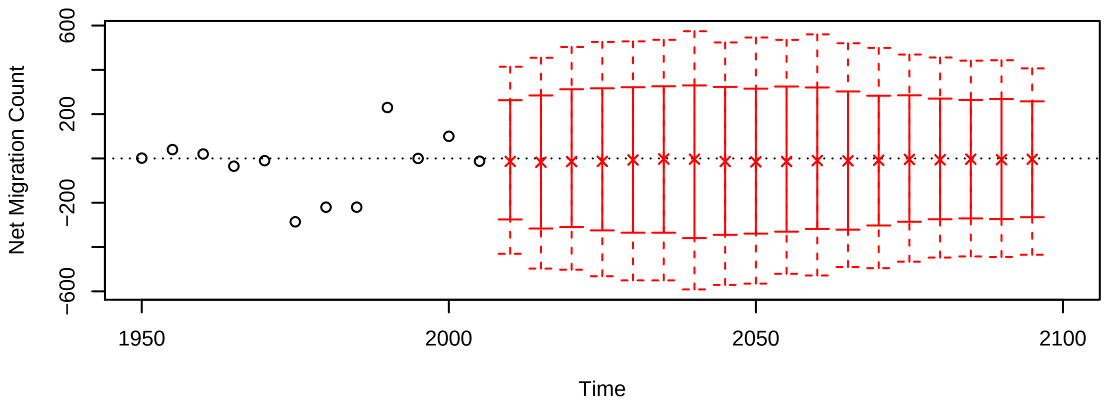

Occupied Palestinian Territory Net Migrants (thousands)

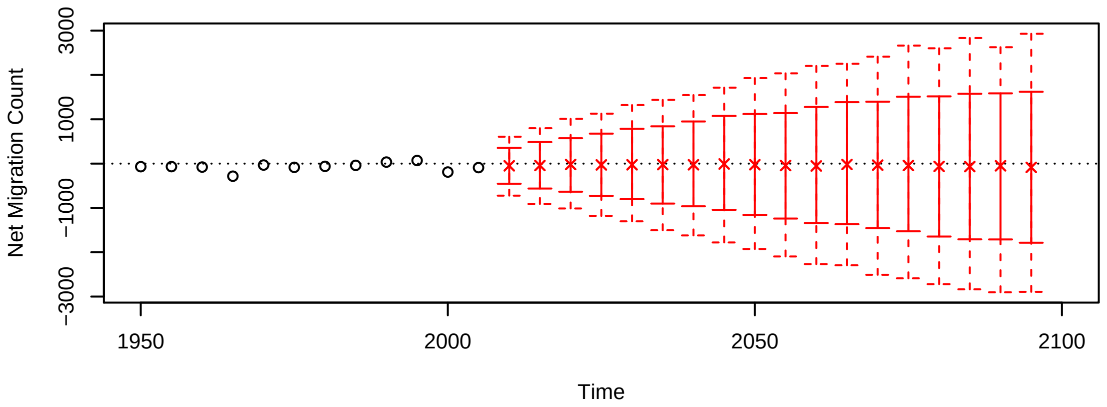

Oman Net Migrants (thousands)

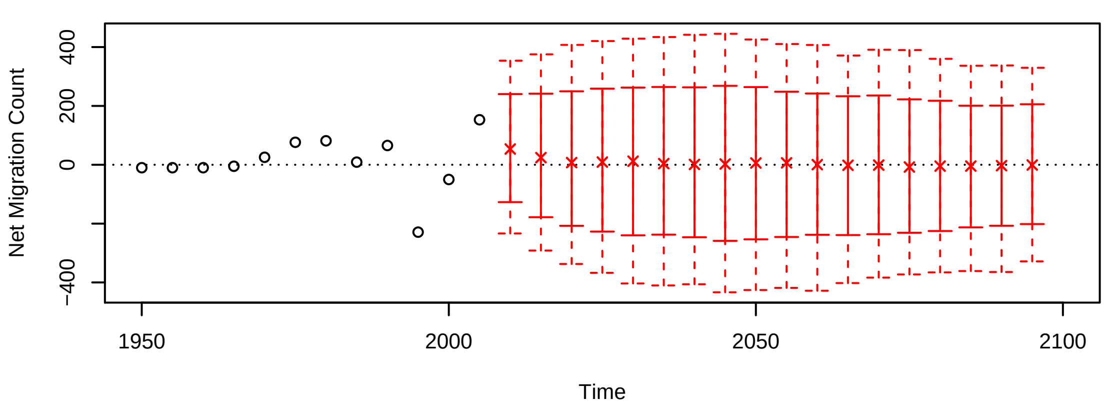

Qatar Net Migrants (thousands)

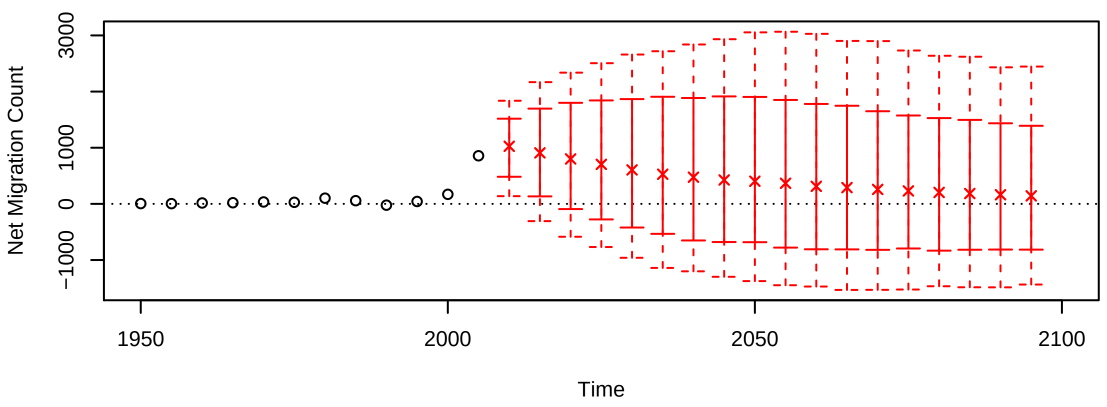

Saudi Arabia Net Migrants (thousands)

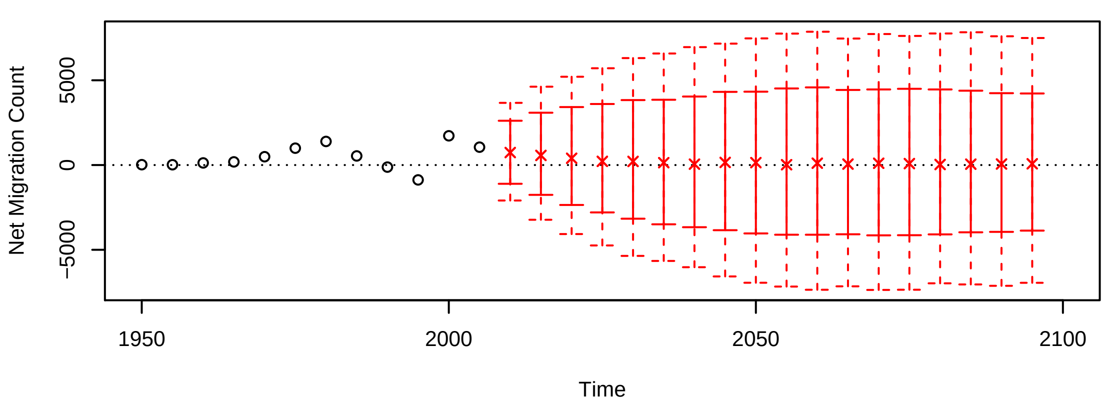

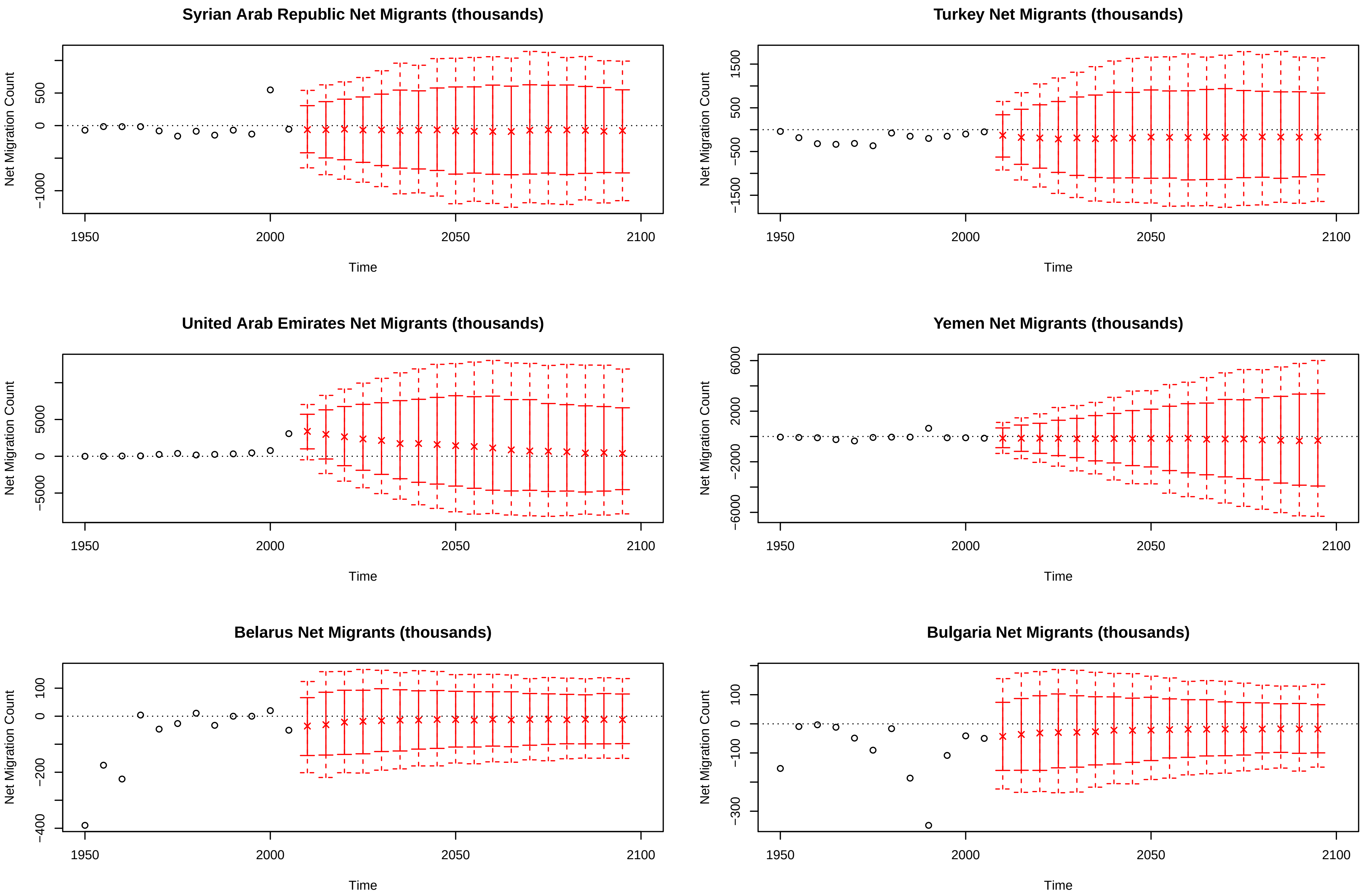

Syrian Arab Republic Net Migrants (thousands)
Turkey Net Migrants (thousands)
United Arab Emirates Net Migrants (thousands)
Yemen Net Migrants (thousands)
Belarus Net Migrants (thousands)
Bulgaria Net Migrants (thousands)
Net Migration Count
Time
1950
2000
2050
2100
−1000
0
500
−1500
−500
500
1500
−5000
0
5000
−6000
−2000
2000
6000
−400
−200
0
100
−300
−100
0
100

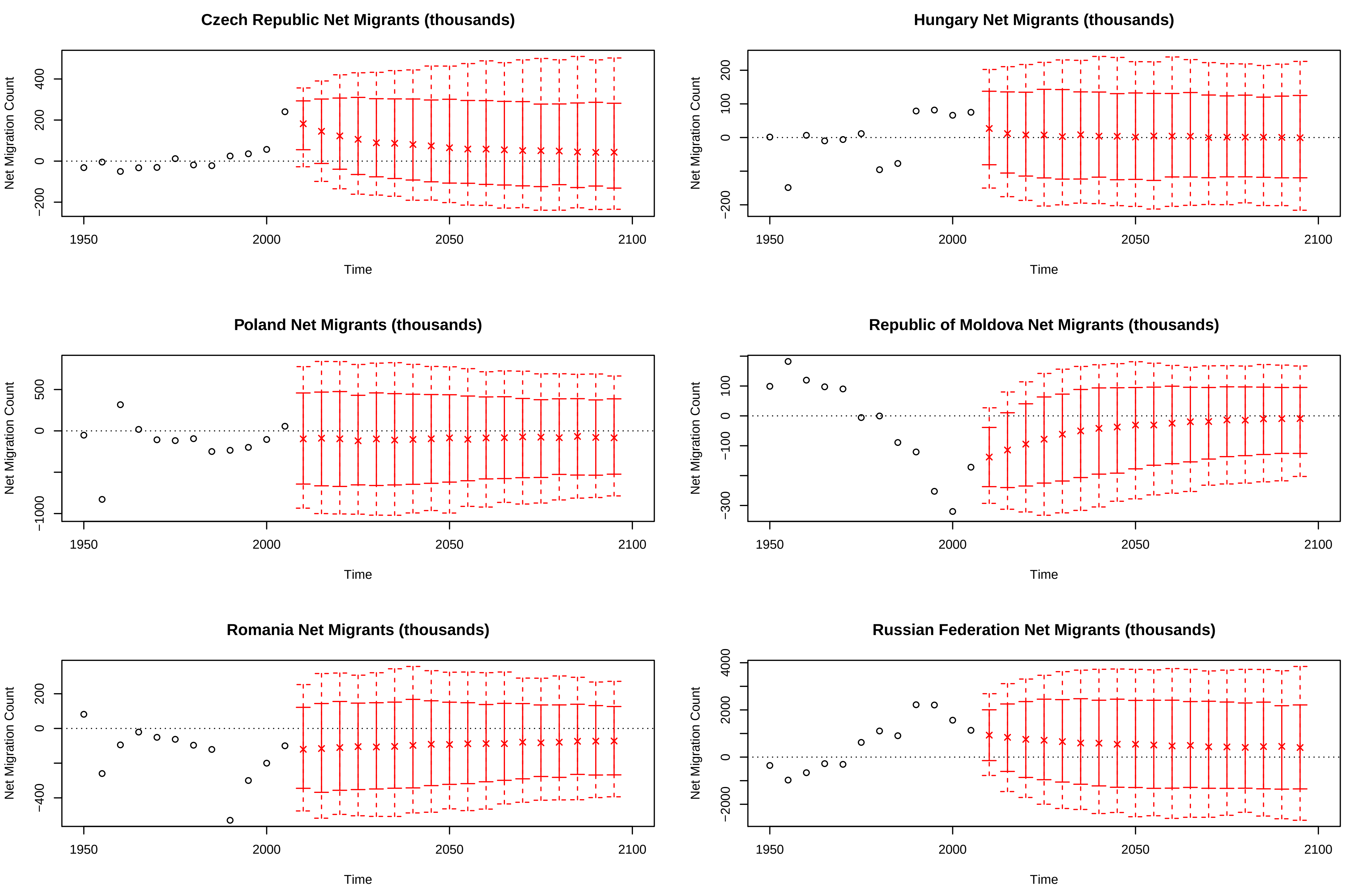
Czech Republic Net Migrants (thousands)
Net Migration Count
−200
0
200
400
1950
2000
2050
2100
Time
Hungary Net Migrants (thousands)
Net Migration Count
−200
0
100
200
1950
2000
2050
2100
Time
Poland Net Migrants (thousands)
Net Migration Count
−1000
0
500
1950
2000
2050
2100
Time
Republic of Moldova Net Migrants (thousands)
Net Migration Count
−300
−100
0
100
1950
2000
2050
2100
Time
Romania Net Migrants (thousands)
Net Migration Count
−400
0
200
1950
2000
2050
2100
Time
Russian Federation Net Migrants (thousands)
Net Migration Count
−2000
0
2000
4000
1950
2000
2050
2100
Time

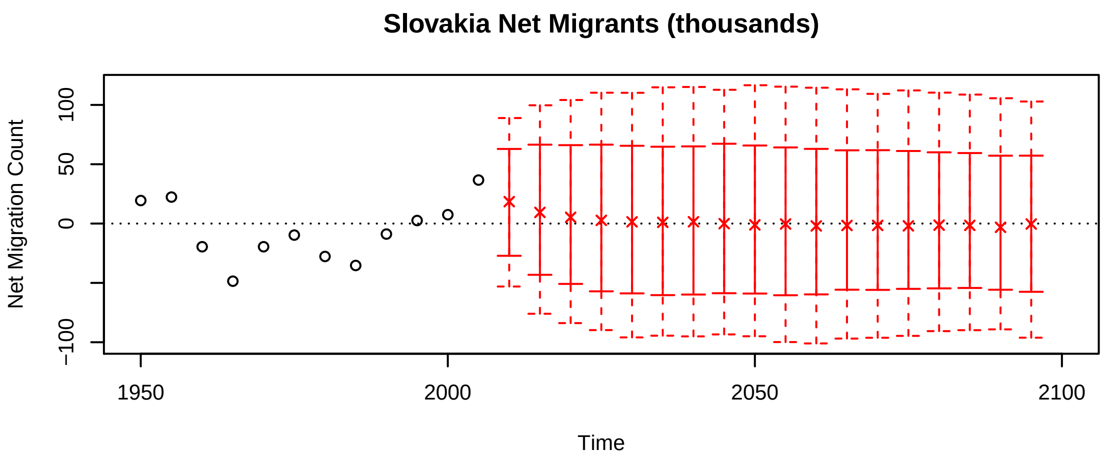
Slovakia Net Migrants (thousands)
Net Migration Count
−100
0
50
100
1950
2000
2050
2100
Time

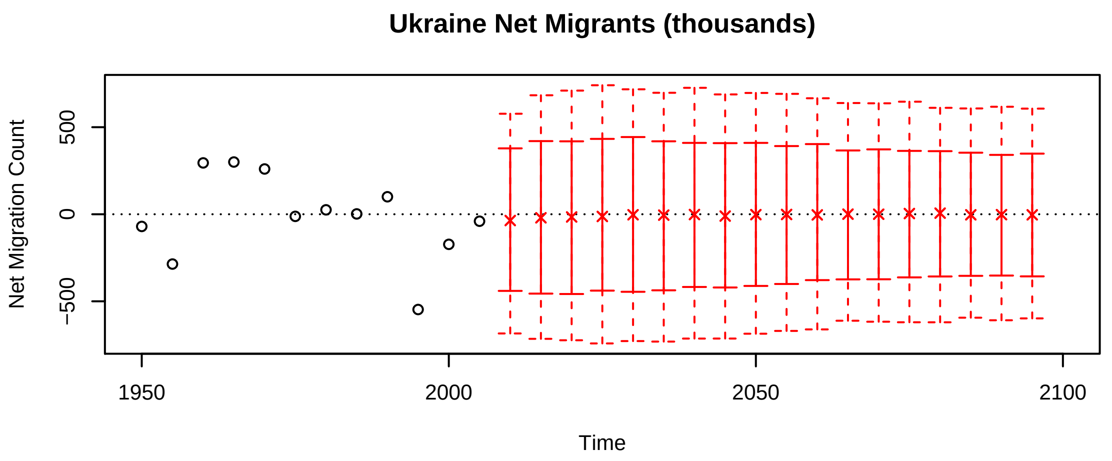
Ukraine Net Migrants (thousands)
Net Migration Count
−500
0
500
1950
2000
2050
2100
Time

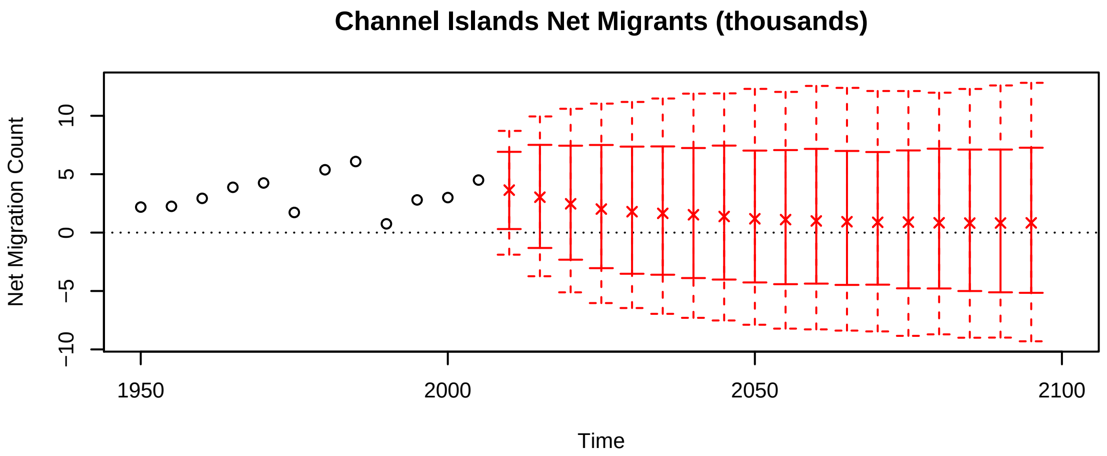
Channel Islands Net Migrants (thousands)
Net Migration Count
−10
−5
0
5
10
1950
2000
2050
2100
Time

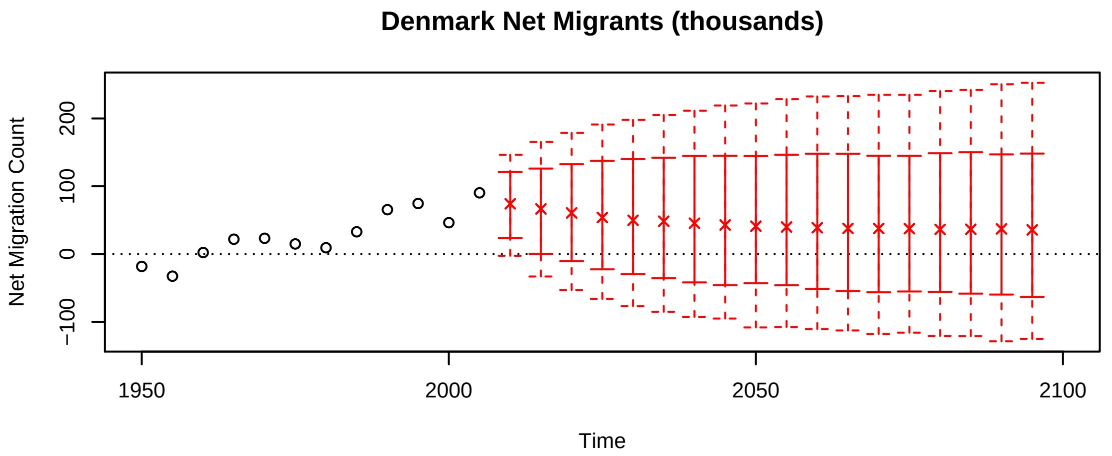
Denmark Net Migrants (thousands)
Net Migration Count
−100
0
100
200
1950
2000
2050
2100
Time

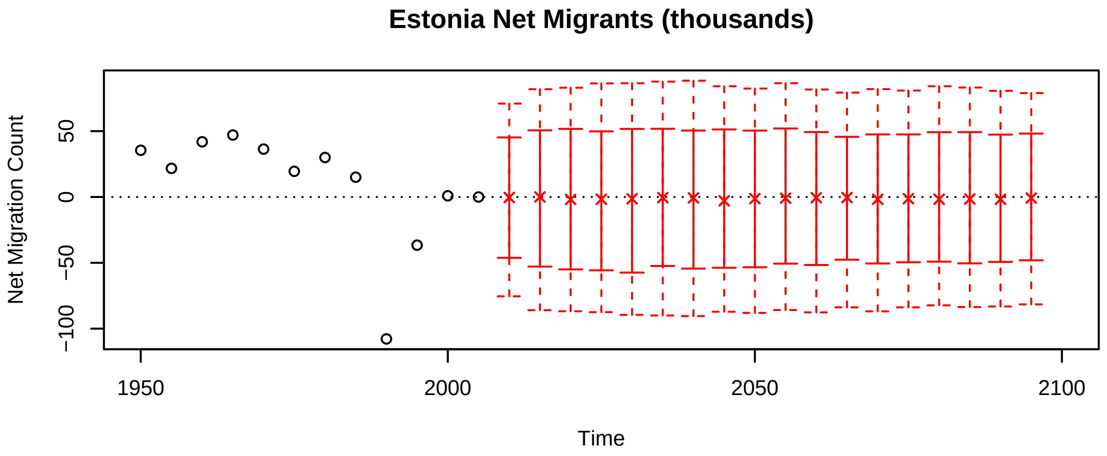
Estonia Net Migrants (thousands)
Net Migration Count
−100
−50
0
50
1950
2000
2050
2100
Time

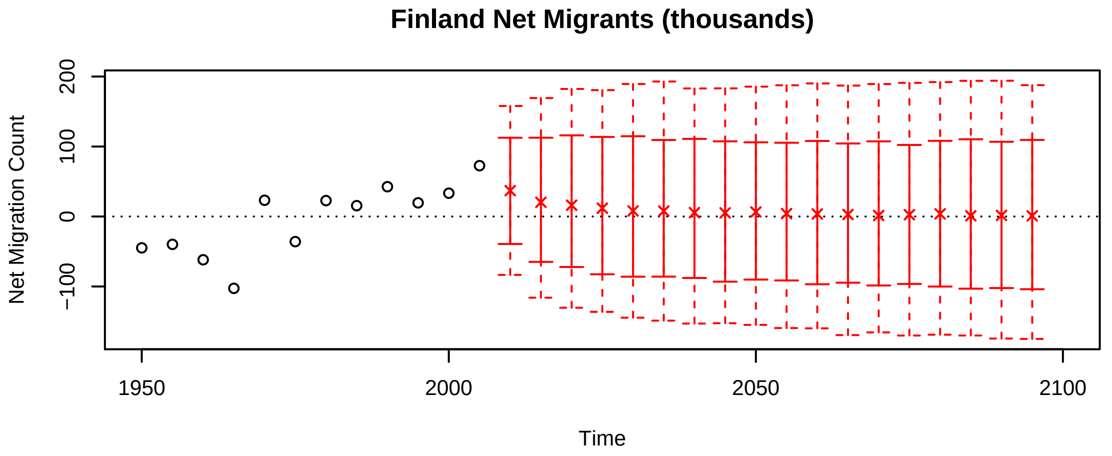
Finland Net Migrants (thousands)
Net Migration Count
−100
0
100
200
1950
2000
2050
2100
Time

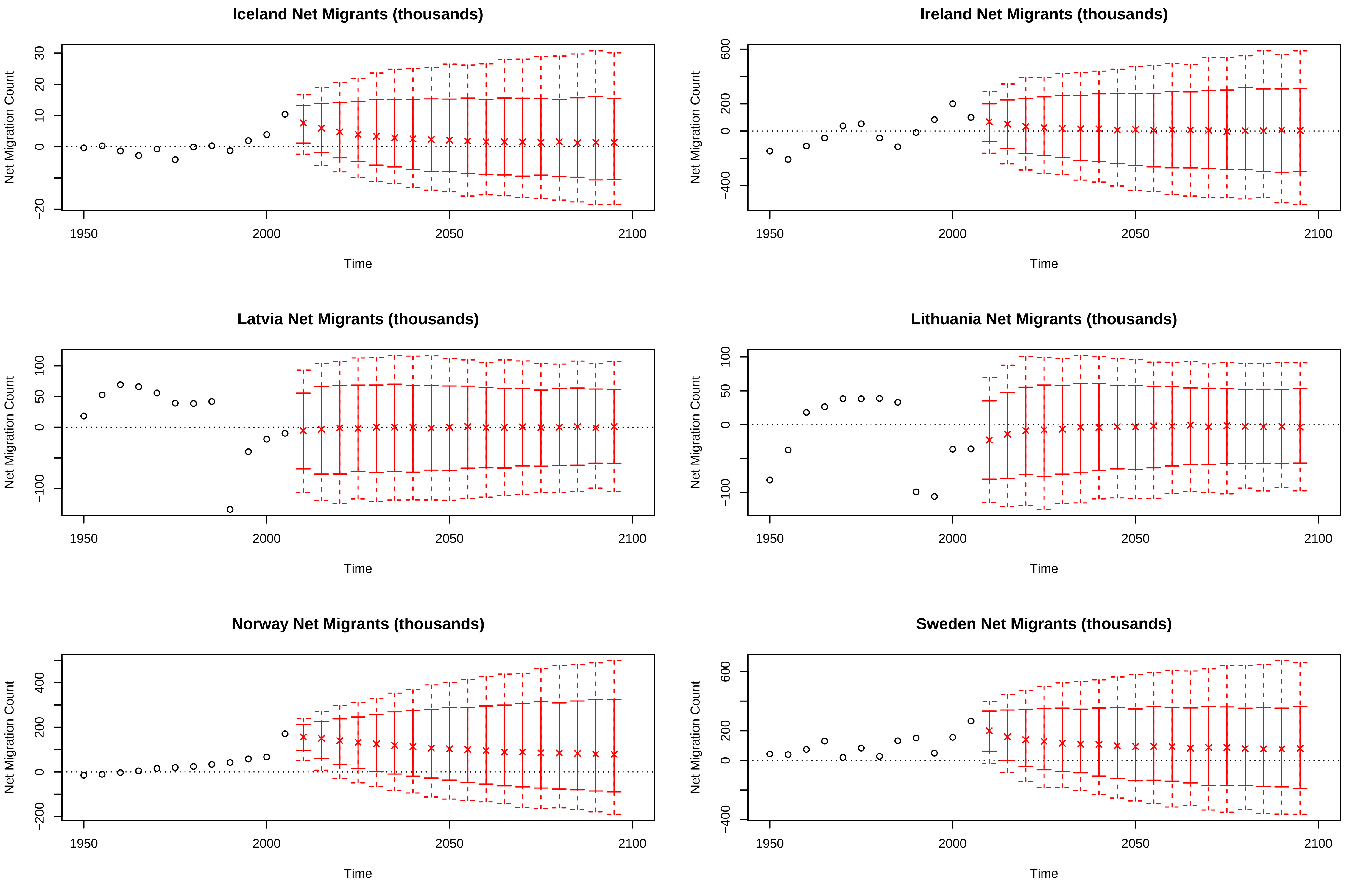
Iceland Net Migrants (thousands)
Ireland Net Migrants (thousands)
Latvia Net Migrants (thousands)
Lithuania Net Migrants (thousands)
Norway Net Migrants (thousands)
Sweden Net Migrants (thousands)
Net Migration Count
Time
1950
2000
2050
2100

United Kingdom Net Migrants (thousands)

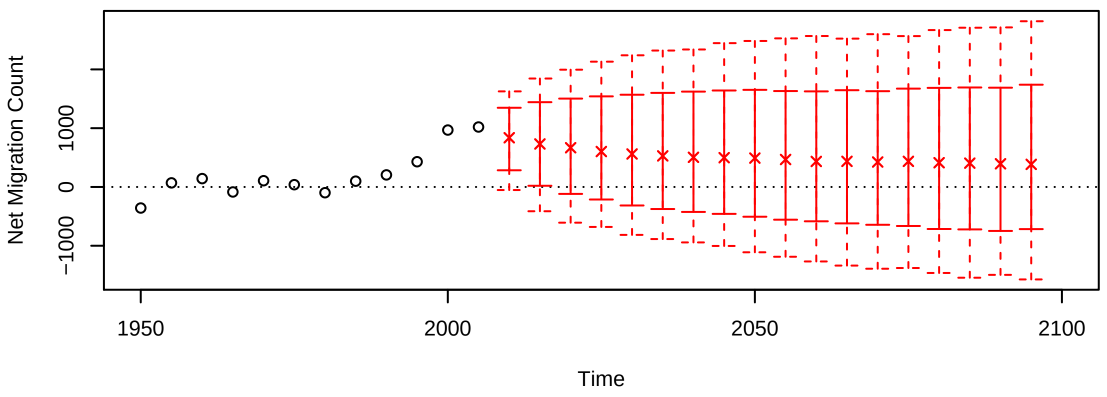

Albania Net Migrants (thousands)

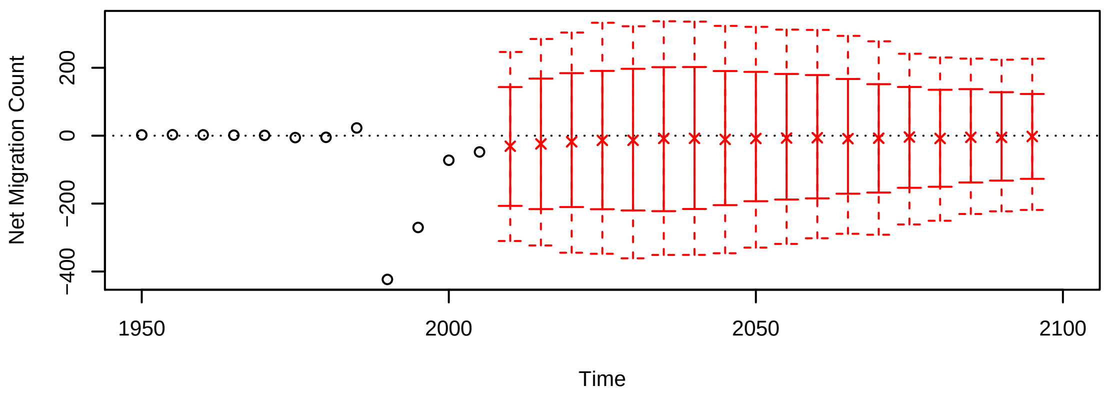

Bosnia and Herzegovina Net Migrants (thousands)

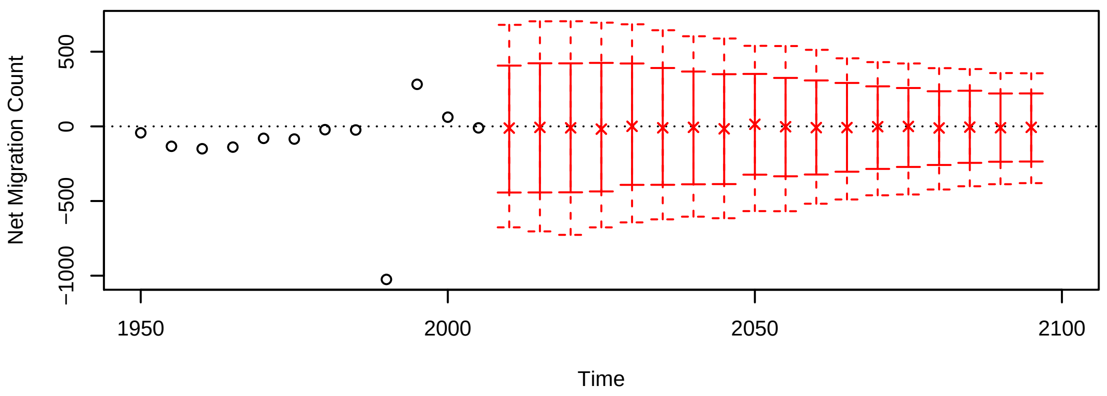

Croatia Net Migrants (thousands)

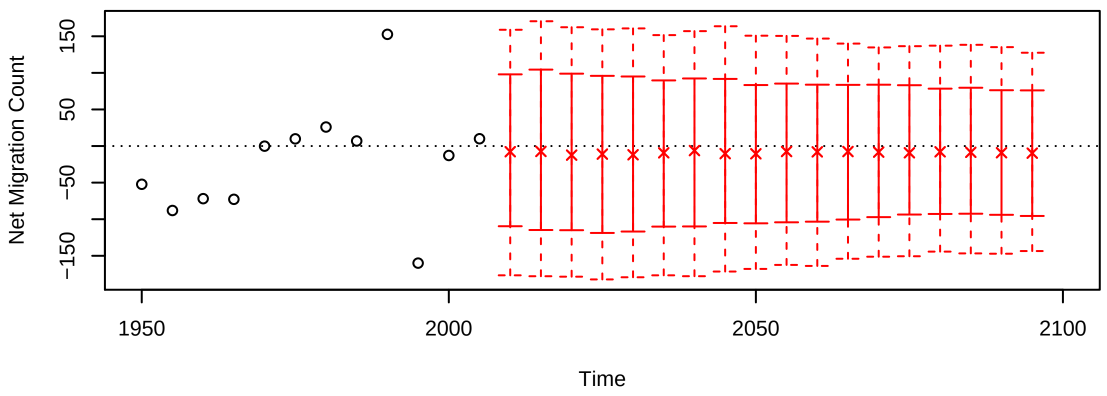

Greece Net Migrants (thousands)

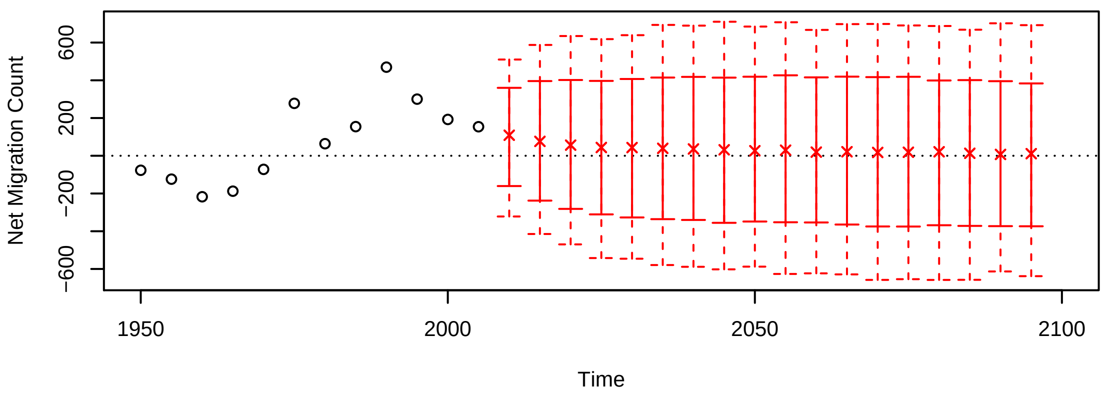

Italy Net Migrants (thousands)

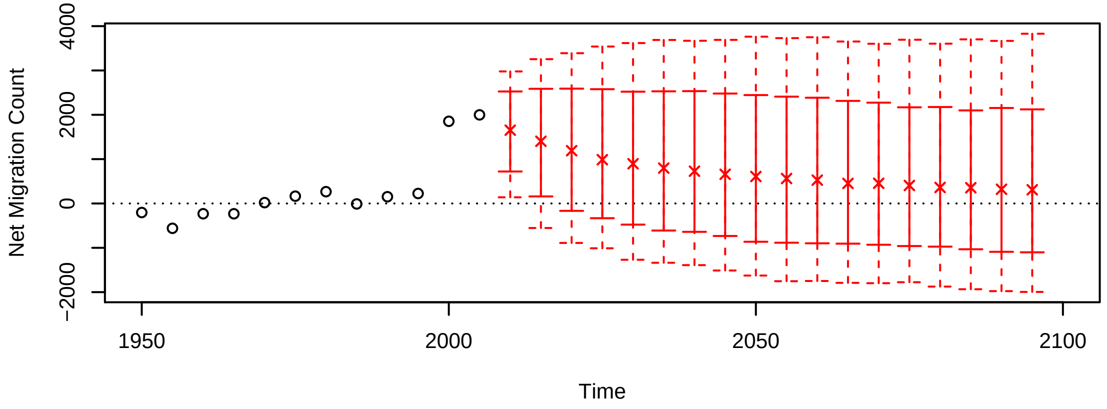

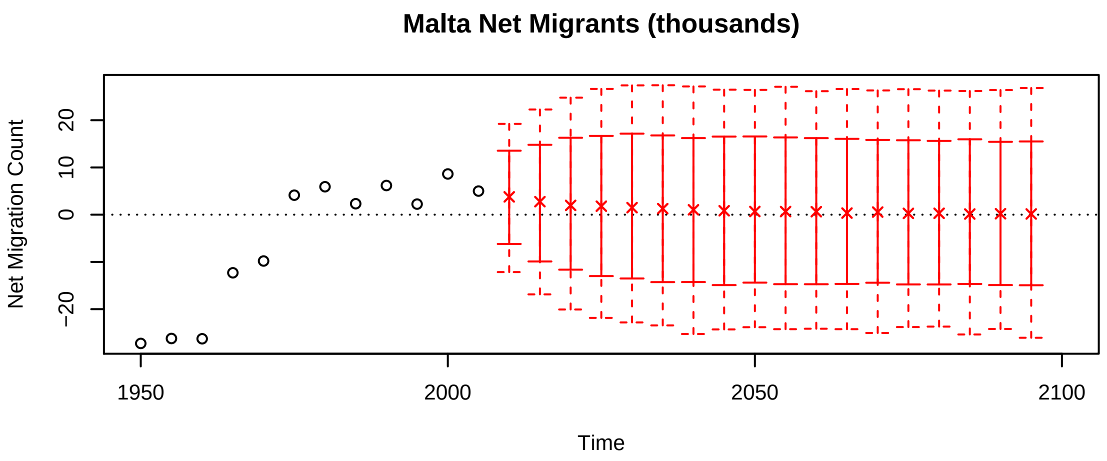
Malta Net Migrants (thousands)
Net Migration Count
20
10
0
−20
1950
2000
2050
2100
Time

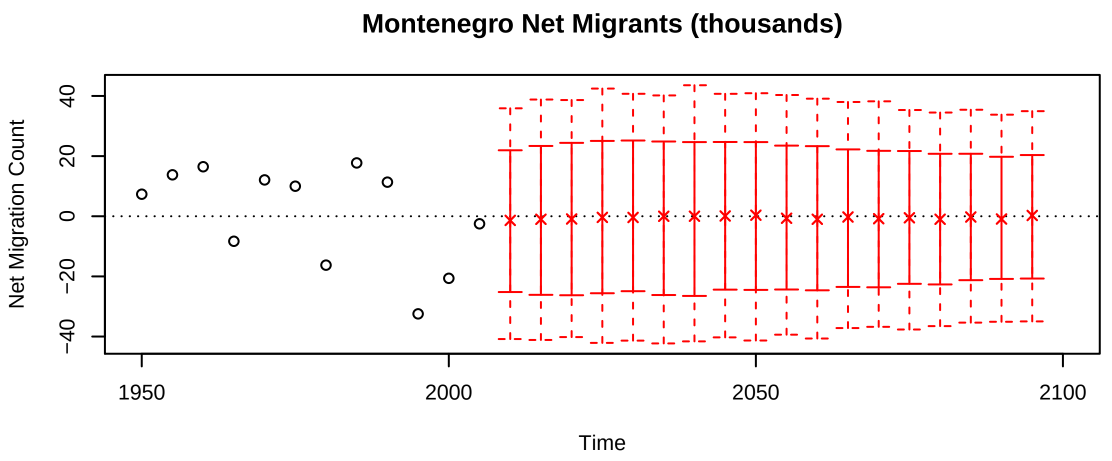
Montenegro Net Migrants (thousands)
Net Migration Count
40
20
0
−20
−40
1950
2000
2050
2100
Time

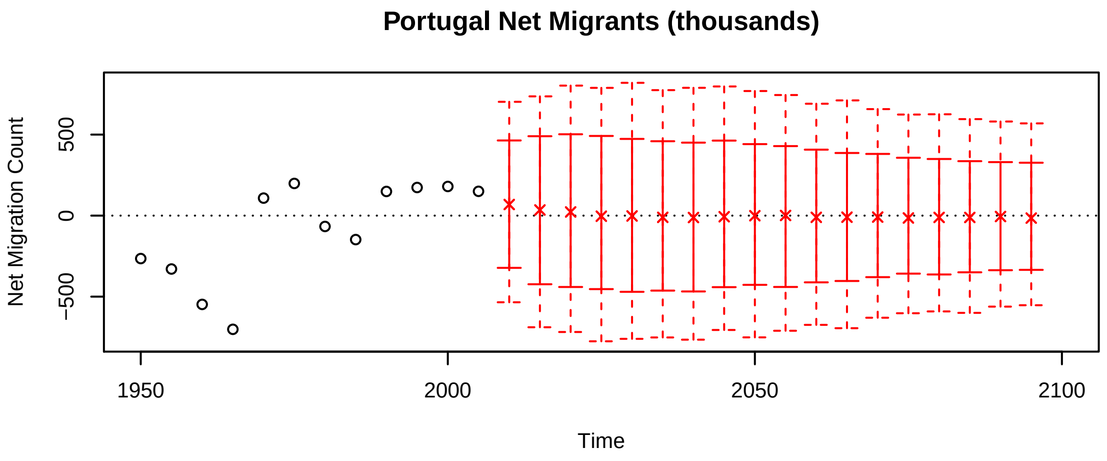
Portugal Net Migrants (thousands)
Net Migration Count
500
0
−500
1950
2000
2050
2100
Time

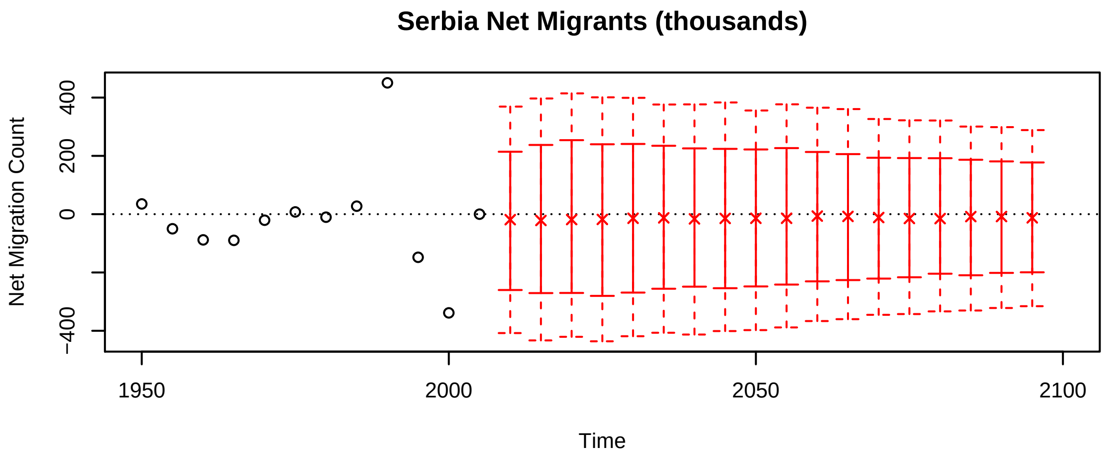
Serbia Net Migrants (thousands)
Net Migration Count
400
200
0
−400
1950
2000
2050
2100
Time

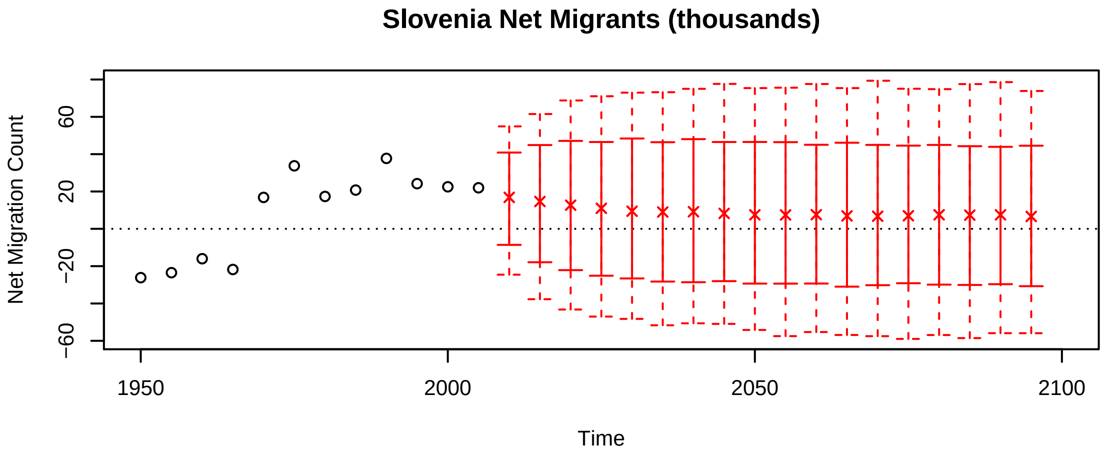
Slovenia Net Migrants (thousands)
Net Migration Count
60
20
−20
−60
1950
2000
2050
2100
Time

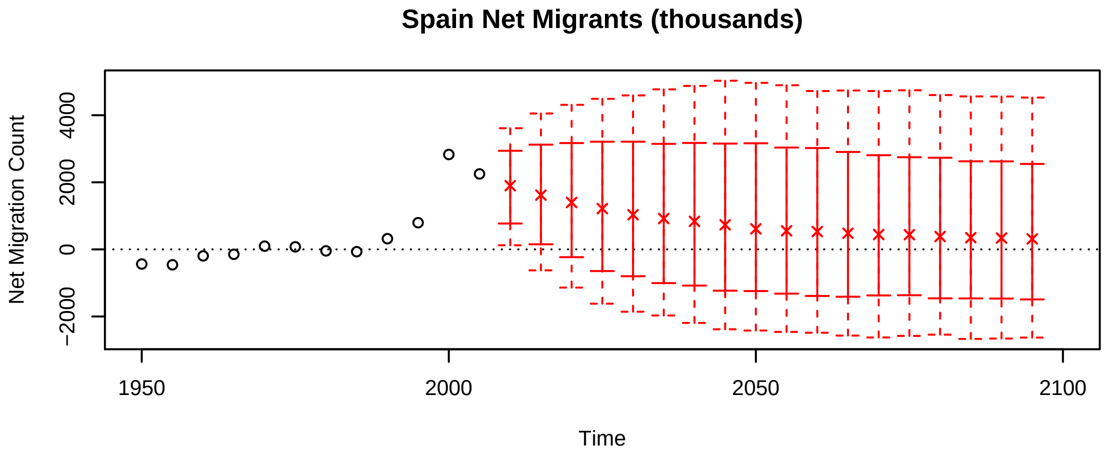
Spain Net Migrants (thousands)
Net Migration Count
4000
2000
0
−2000
1950
2000
2050
2100
Time

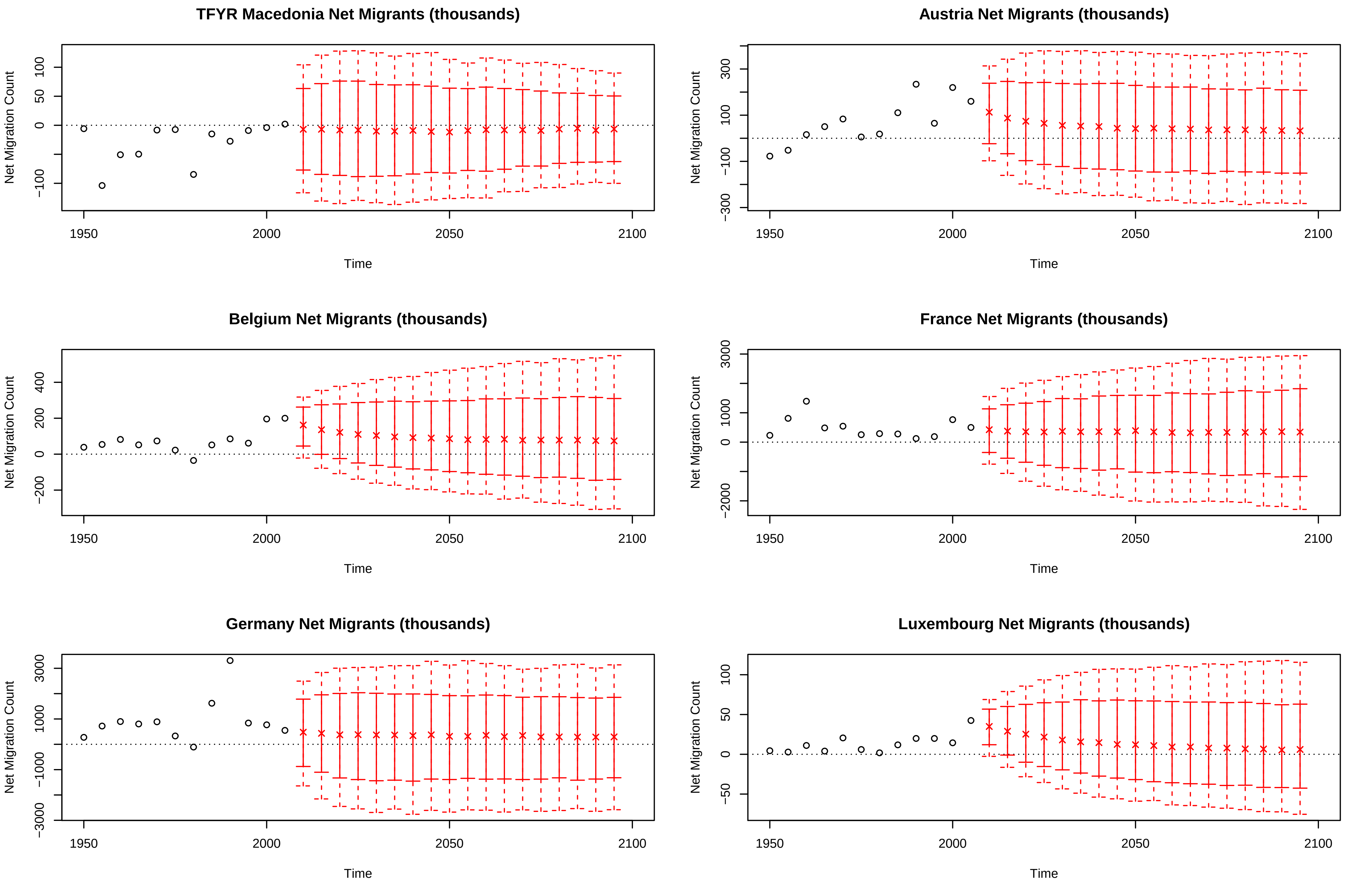
TFYR Macedonia Net Migrants (thousands)
Austria Net Migrants (thousands)
Belgium Net Migrants (thousands)
France Net Migrants (thousands)
Germany Net Migrants (thousands)
Luxembourg Net Migrants (thousands)
Net Migration Count
Time
1950
2000
2050
2100

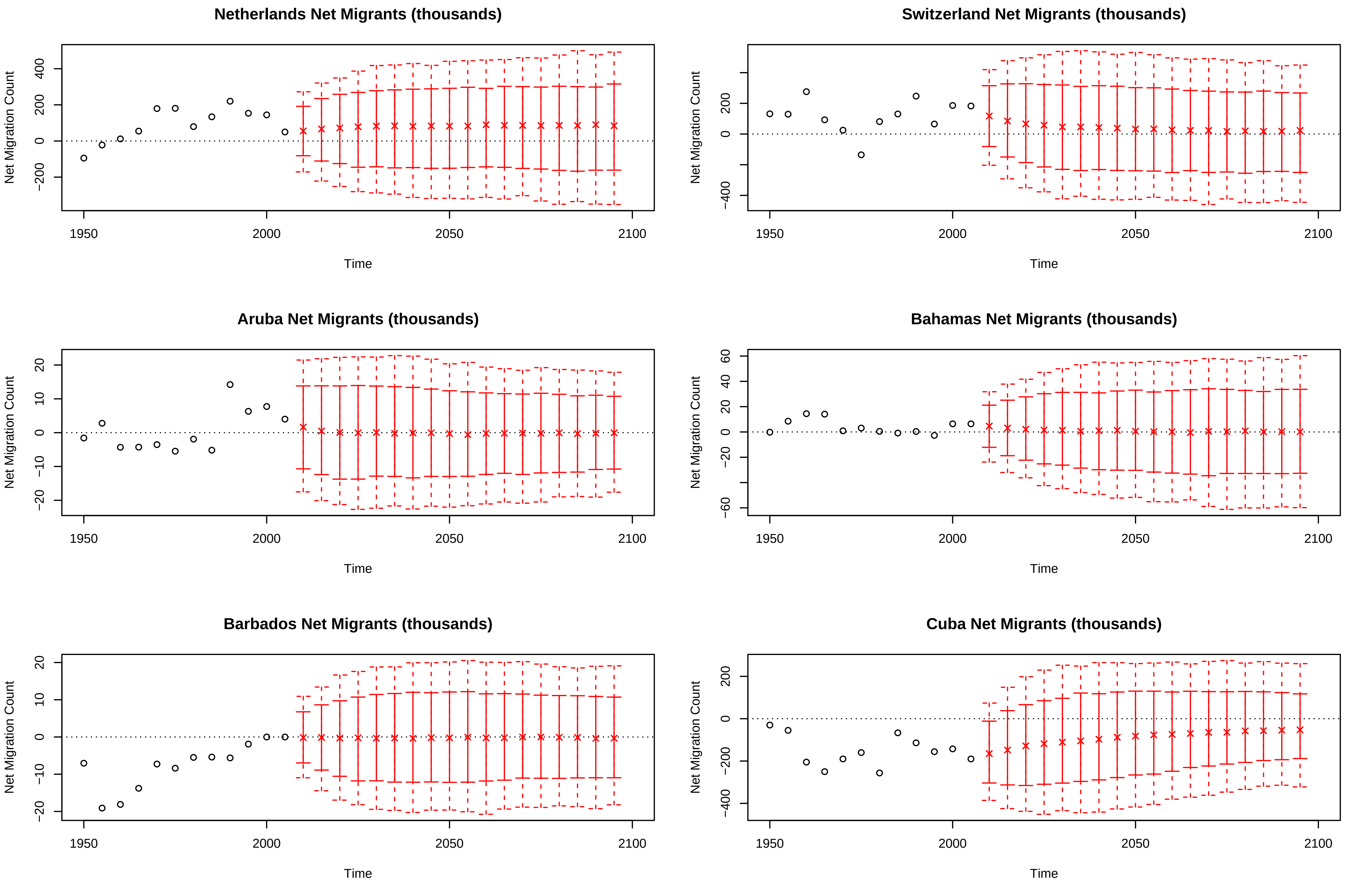
Netherlands Net Migrants (thousands)
Switzerland Net Migrants (thousands)
Aruba Net Migrants (thousands)
Bahamas Net Migrants (thousands)
Barbados Net Migrants (thousands)
Cuba Net Migrants (thousands)
Net Migration Count
Time
1950
2000
2050
2100
−200
0
200
400
−400
−20
−10
10
20
−60
−20
0
20
40
60

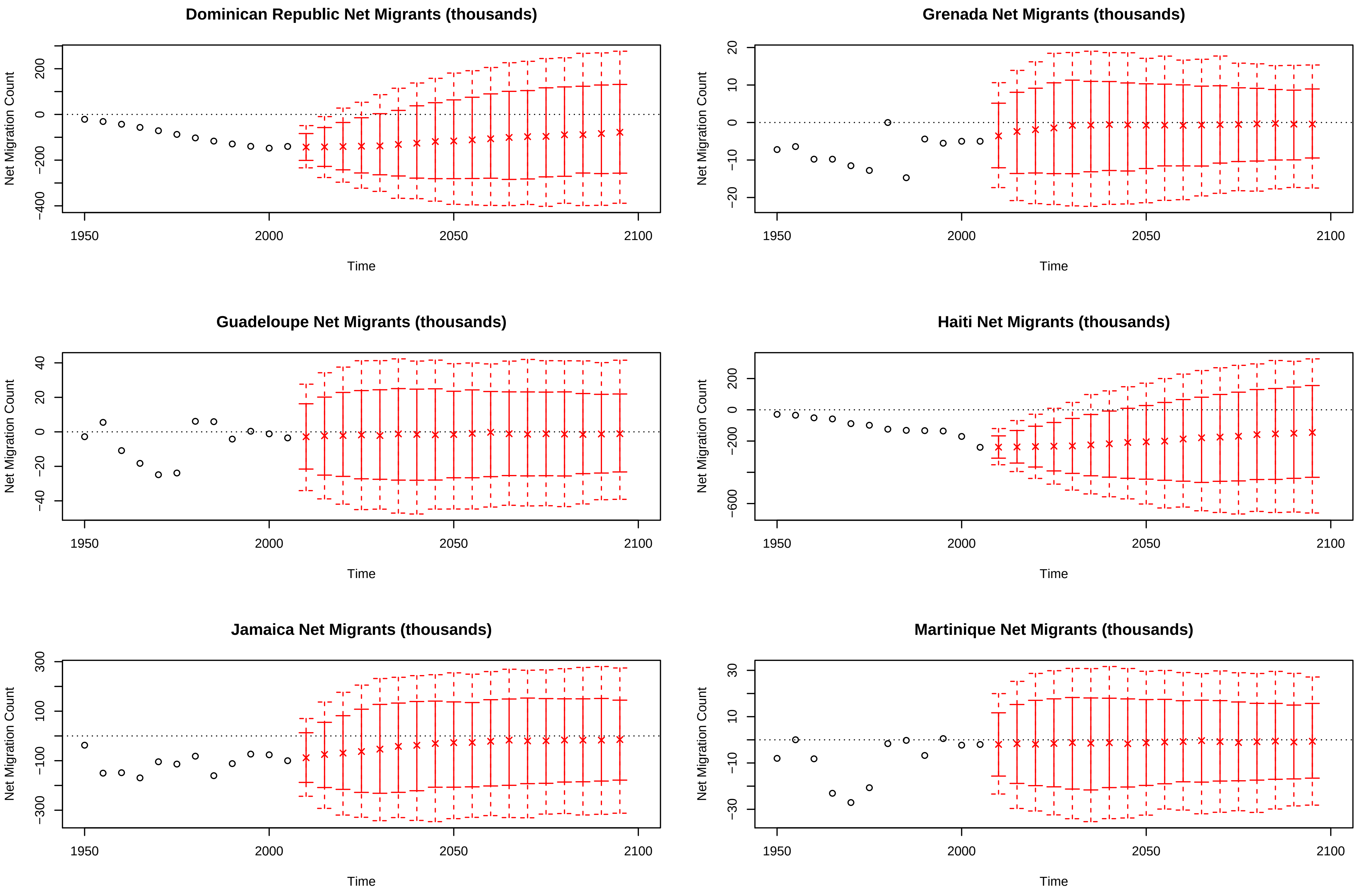
Dominican Republic Net Migrants (thousands)
Grenada Net Migrants (thousands)
Guadeloupe Net Migrants (thousands)
Haiti Net Migrants (thousands)
Jamaica Net Migrants (thousands)
Martinique Net Migrants (thousands)
Net Migration Count
Time
1950
2000
2050
2100
−400
−200
0
200
−20
−10
0
10
20
−40
−20
0
20
40
−600
−200
0
200
−300
−100
100
300
−30
−10
10
30

Netherlands Antilles Net Migrants (thousands)

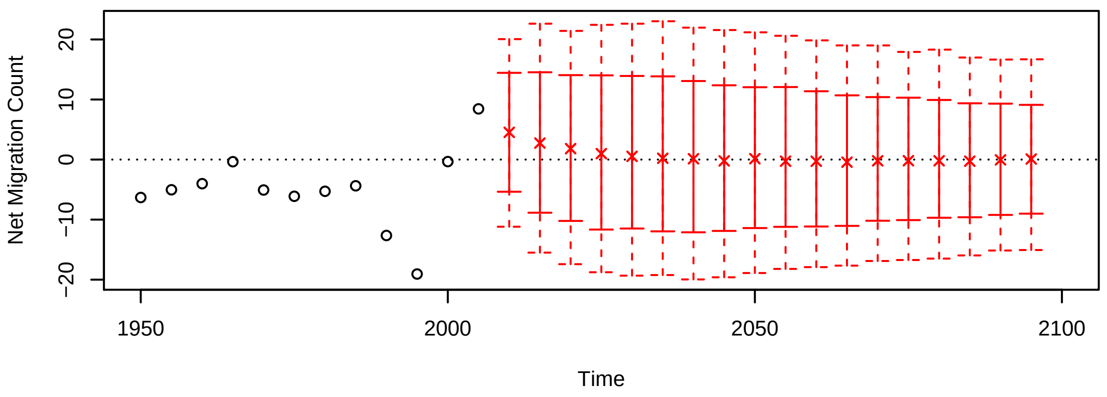

Puerto Rico Net Migrants (thousands)

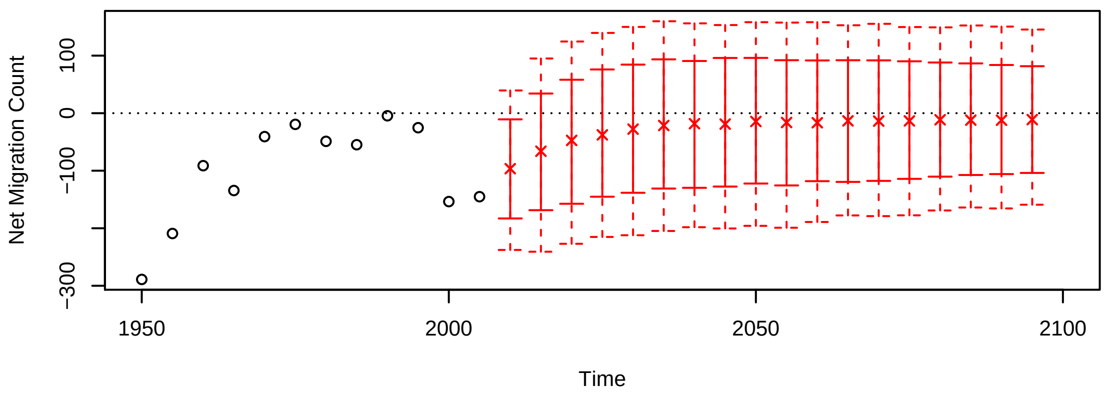

Saint Lucia Net Migrants (thousands)

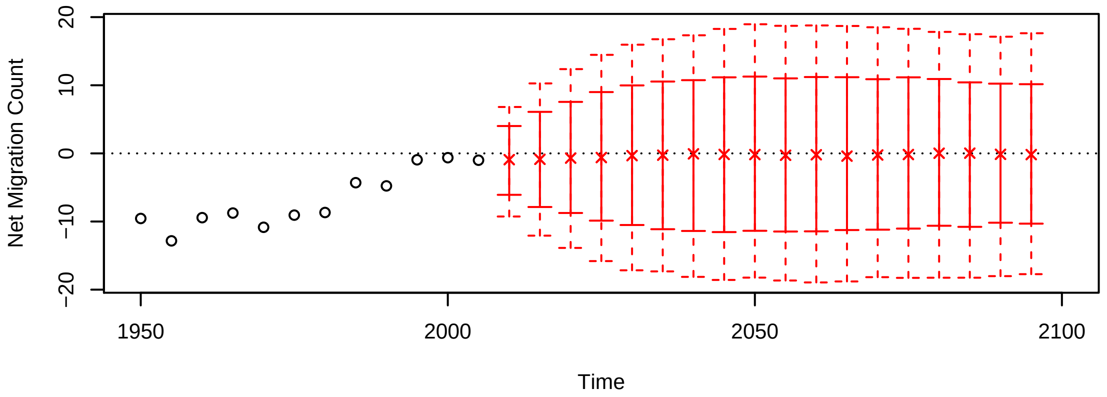

Saint Vincent and the Grenadines Net Migrants (thousands)

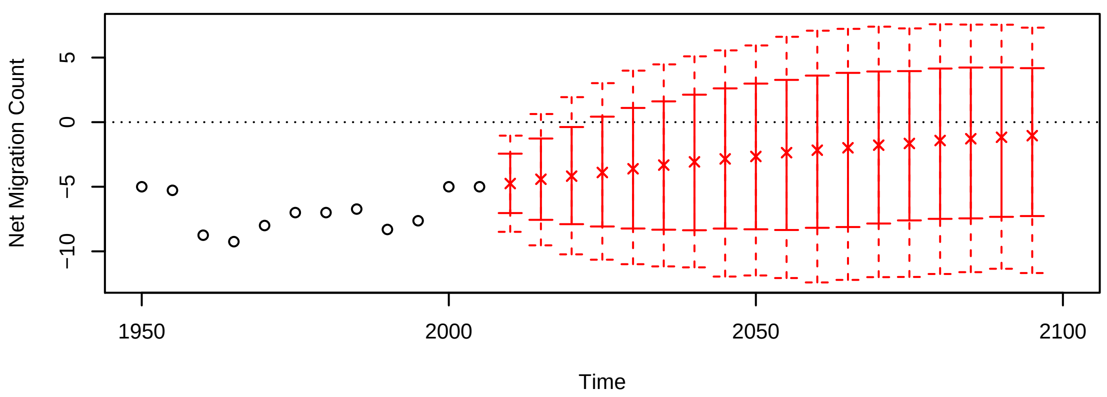

Trinidad and Tobago Net Migrants (thousands)

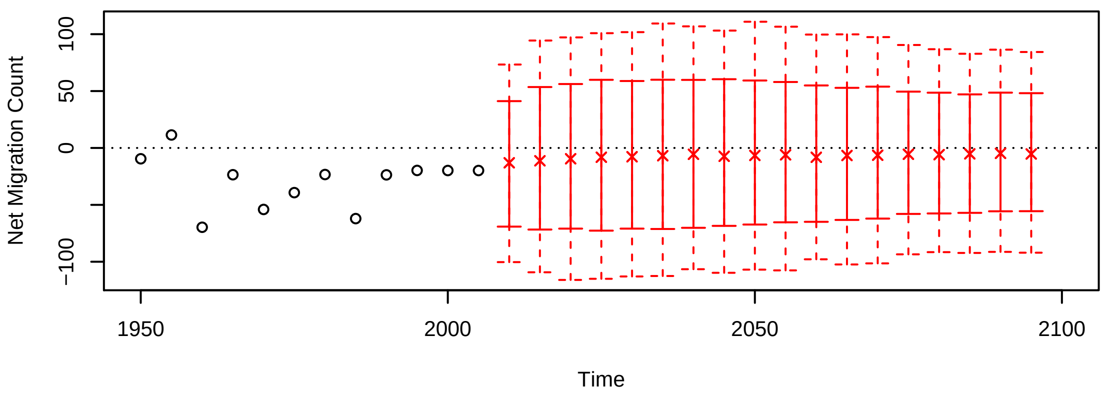

United States Virgin Islands Net Migrants (thousands)

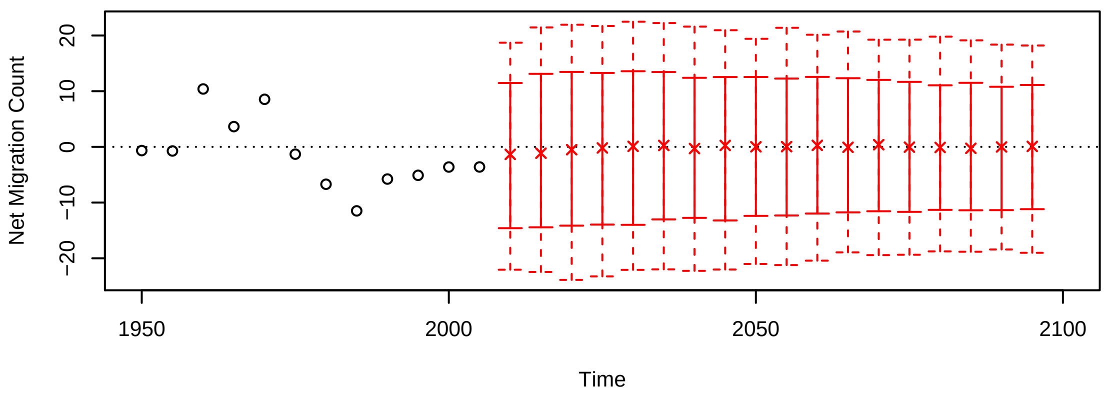

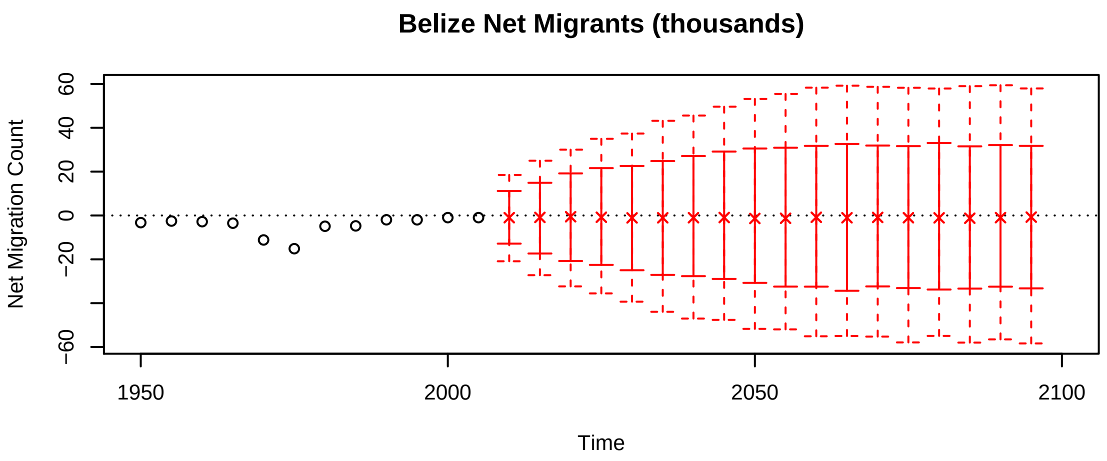
Belize Net Migrants (thousands)
Net Migration Count
60
40
20
0
−20
−60
1950
2000
2050
2100
Time

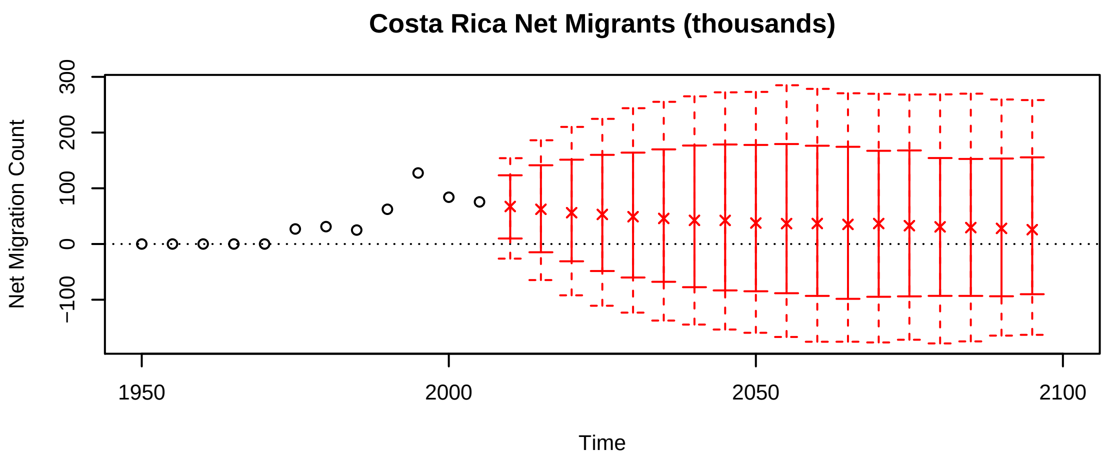
Costa Rica Net Migrants (thousands)
Net Migration Count
300
200
100
0
−100
1950
2000
2050
2100
Time

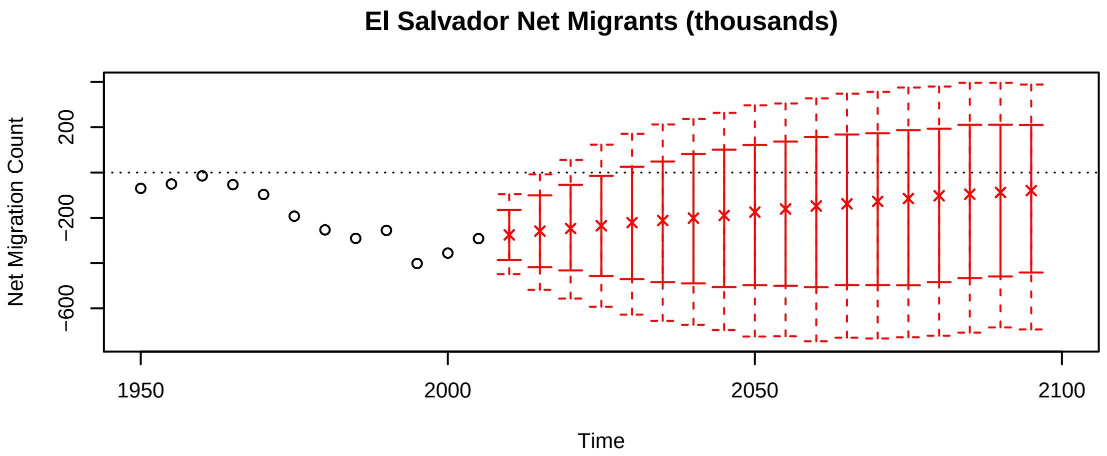
El Salvador Net Migrants (thousands)
Net Migration Count
200
−200
−600
1950
2000
2050
2100
Time

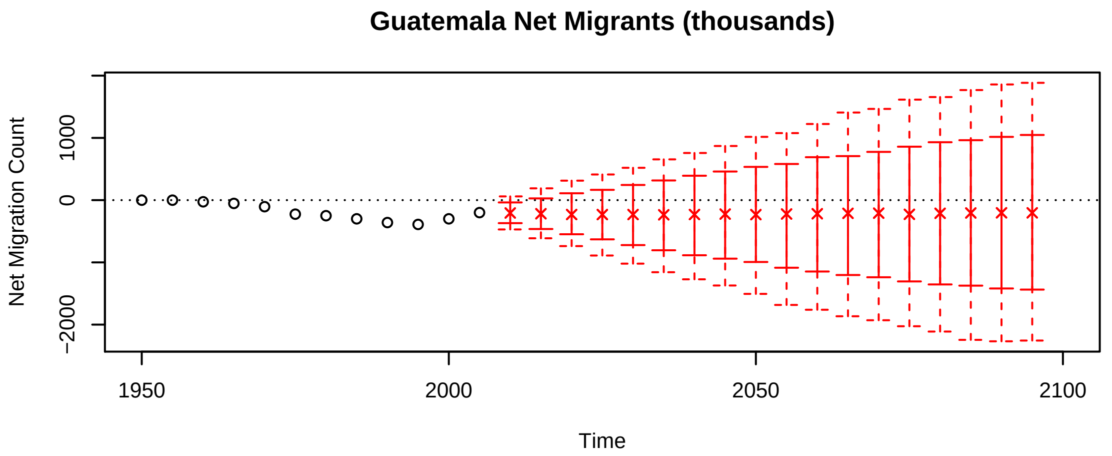
Guatemala Net Migrants (thousands)
Net Migration Count
1000
0
−2000
1950
2000
2050
2100
Time

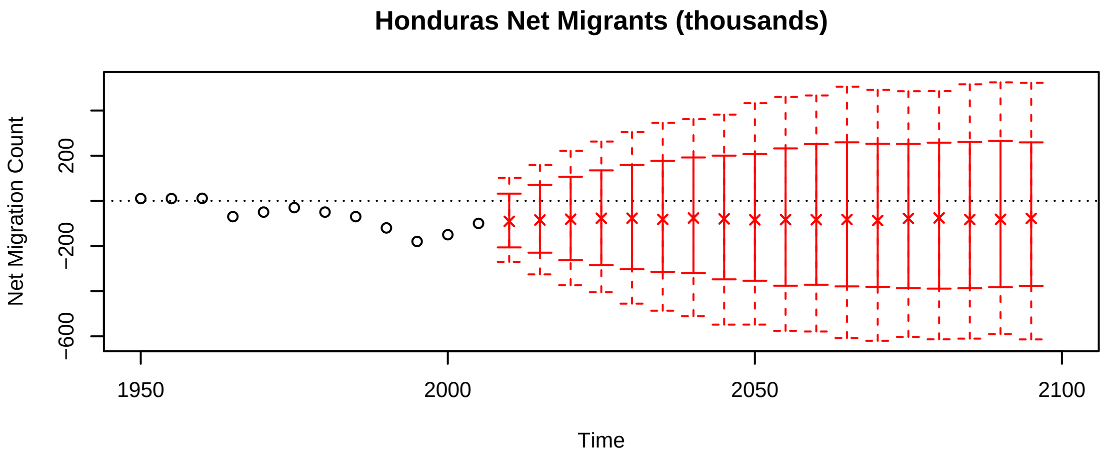
Honduras Net Migrants (thousands)
Net Migration Count
200
−200
−600
1950
2000
2050
2100
Time

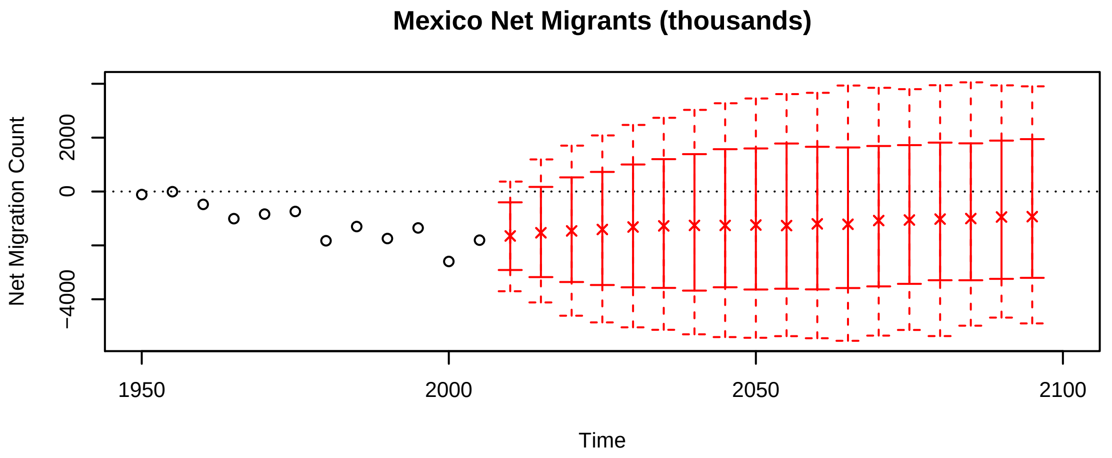
Mexico Net Migrants (thousands)
Net Migration Count
2000
0
−4000
1950
2000
2050
2100
Time

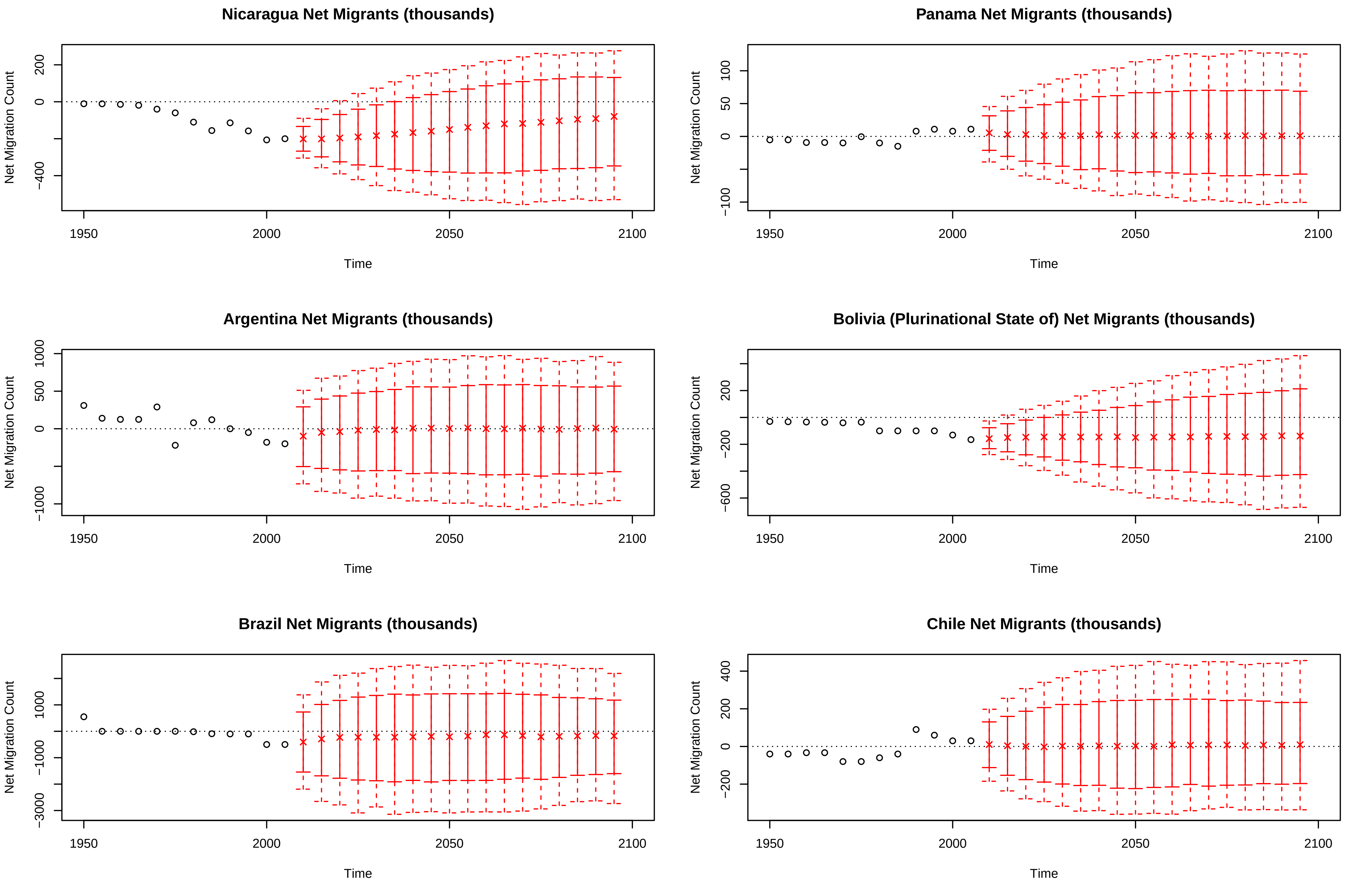

Nicaragua Net Migrants (thousands)
Net Migration Count
200
0
−400
1950
2000
2050
2100
Time
Panama Net Migrants (thousands)
Net Migration Count
100
50
0
−100
1950
2000
2050
2100
Time
Argentina Net Migrants (thousands)
Net Migration Count
1000
500
0
−1000
1950
2000
2050
2100
Time
Bolivia (Plurinational State of) Net Migrants (thousands)
Net Migration Count
200
−200
−600
1950
2000
2050
2100
Time
Brazil Net Migrants (thousands)
Net Migration Count
1000
−1000
−3000
1950
2000
2050
2100
Time
Chile Net Migrants (thousands)
Net Migration Count
400
200
0
−200
1950
2000
2050
2100
Time

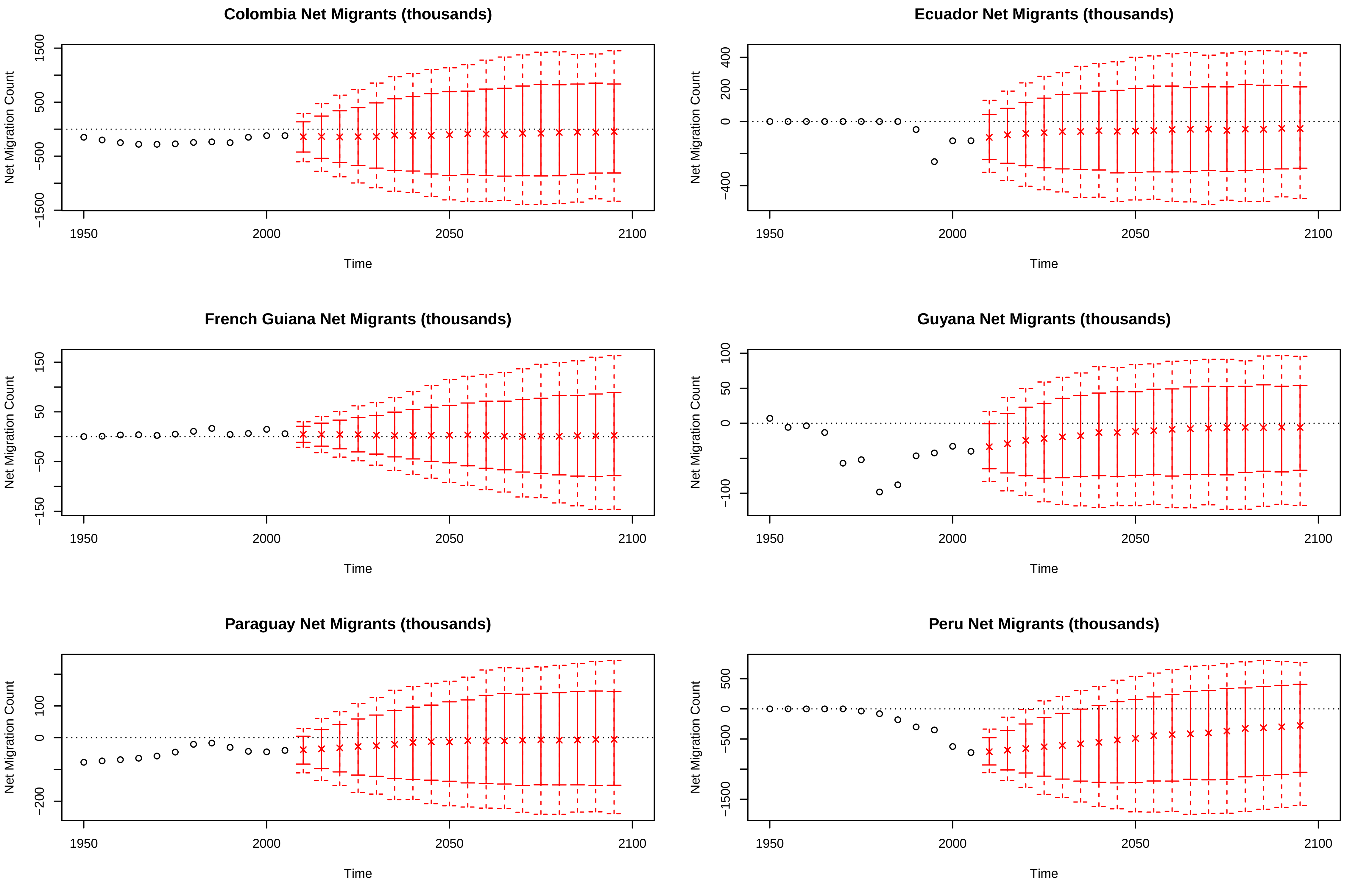
Colombia Net Migrants (thousands)
Ecuador Net Migrants (thousands)
French Guiana Net Migrants (thousands)
Guyana Net Migrants (thousands)
Paraguay Net Migrants (thousands)
Peru Net Migrants (thousands)
Net Migration Count
Time
1950
2000
2050
2100
−1500
−500
500
1500
−400
0
200
400
−150
−50
50
150
−100
100
−200
−1500
−500
0
500

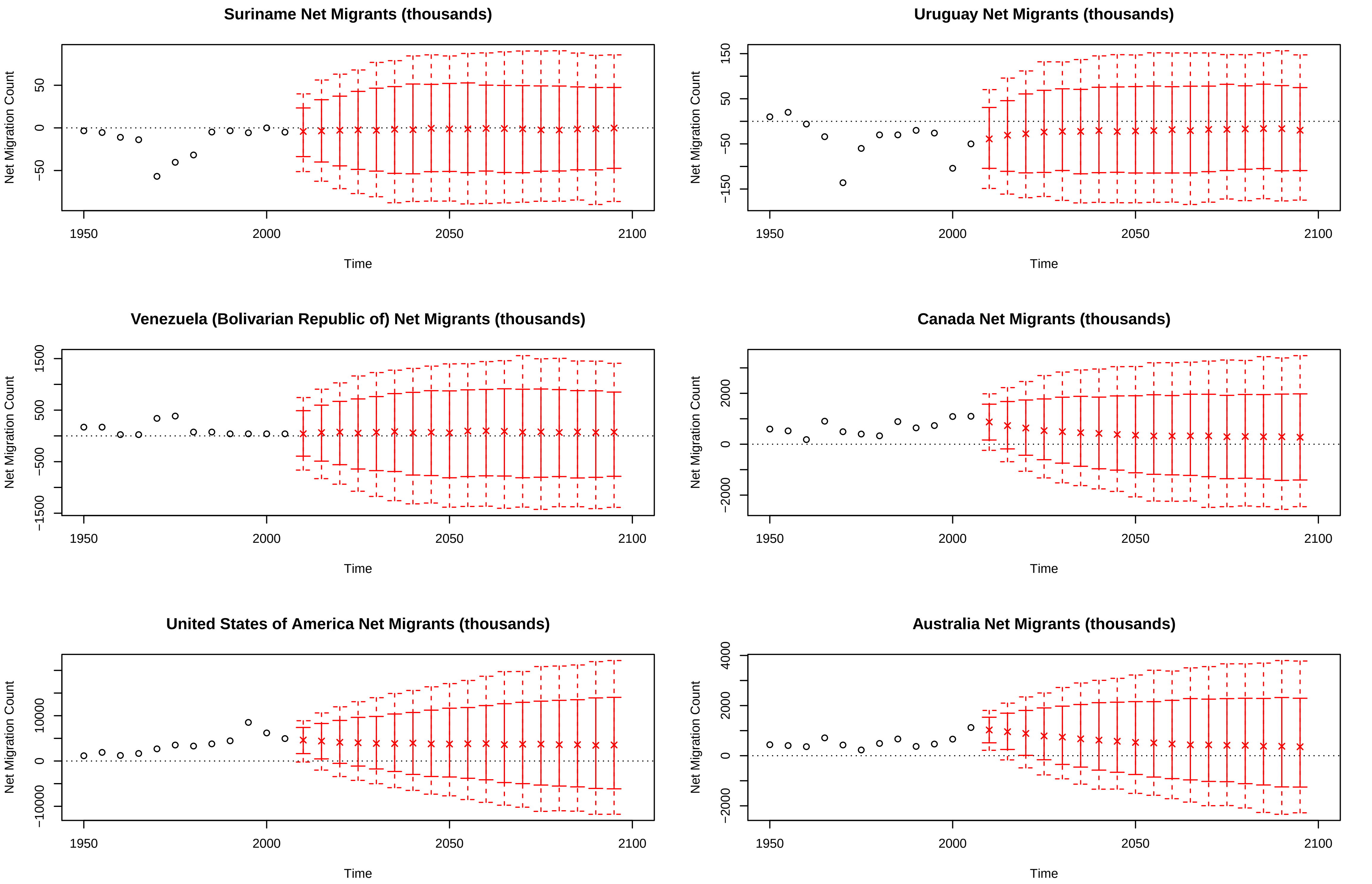

Suriname Net Migrants (thousands)
Net Migration Count
−50
0
50
Time
1950
2000
2050
2100
Uruguay Net Migrants (thousands)
Net Migration Count
−150
−50
50
150
Time
1950
2000
2050
2100
Venezuela (Bolivarian Republic of) Net Migrants (thousands)
Net Migration Count
−1500
−500
500
1500
Time
1950
2000
2050
2100
Canada Net Migrants (thousands)
Net Migration Count
−2000
0
2000
Time
1950
2000
2050
2100
United States of America Net Migrants (thousands)
Net Migration Count
−10000
0
10000
Time
1950
2000
2050
2100
Australia Net Migrants (thousands)
Net Migration Count
−2000
0
2000
4000
Time
1950
2000
2050
2100

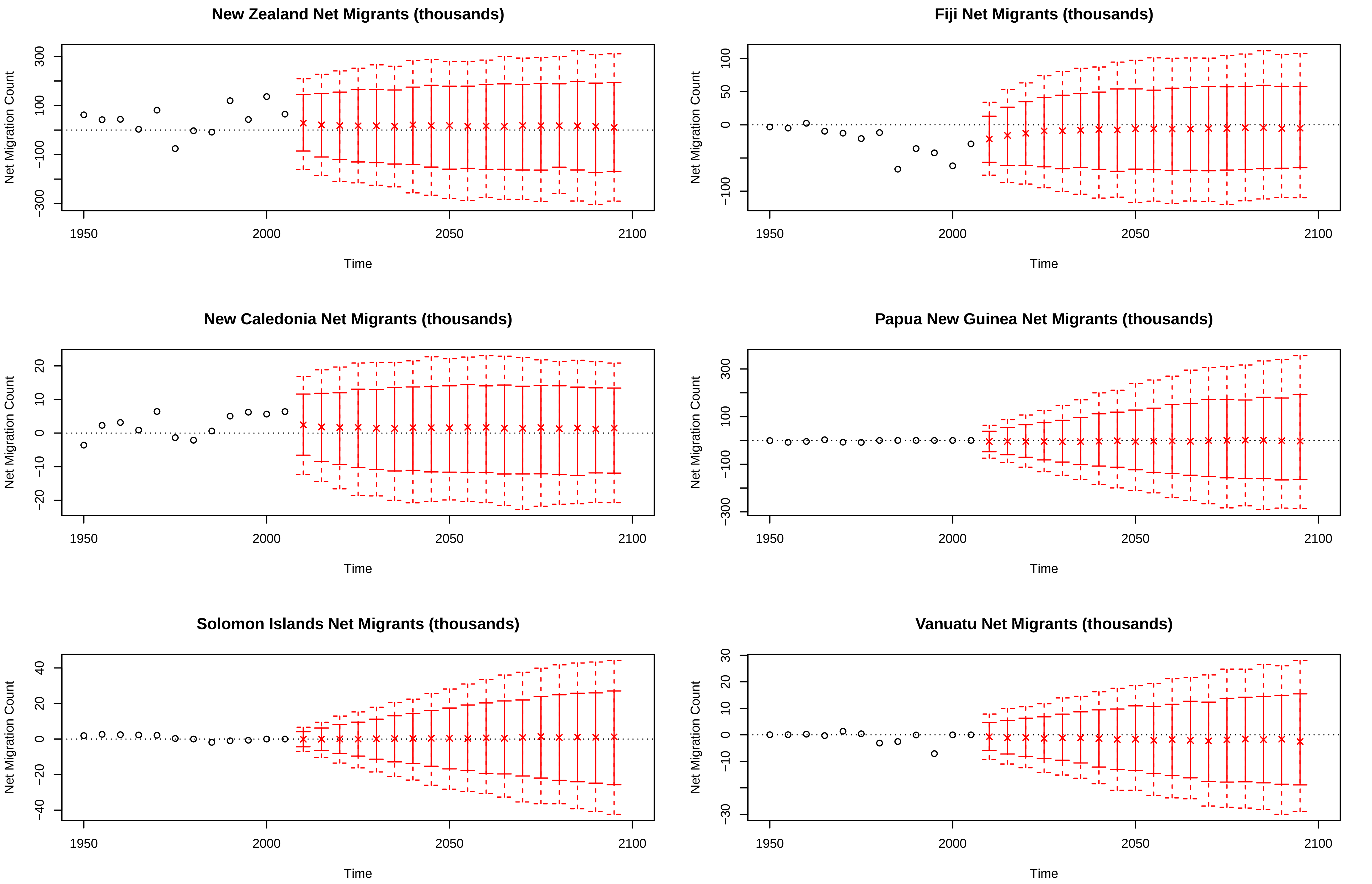
New Zealand Net Migrants (thousands)
Fiji Net Migrants (thousands)
New Caledonia Net Migrants (thousands)
Papua New Guinea Net Migrants (thousands)
Solomon Islands Net Migrants (thousands)
Vanuatu Net Migrants (thousands)
Net Migration Count
Time
1950
2000
2050
2100

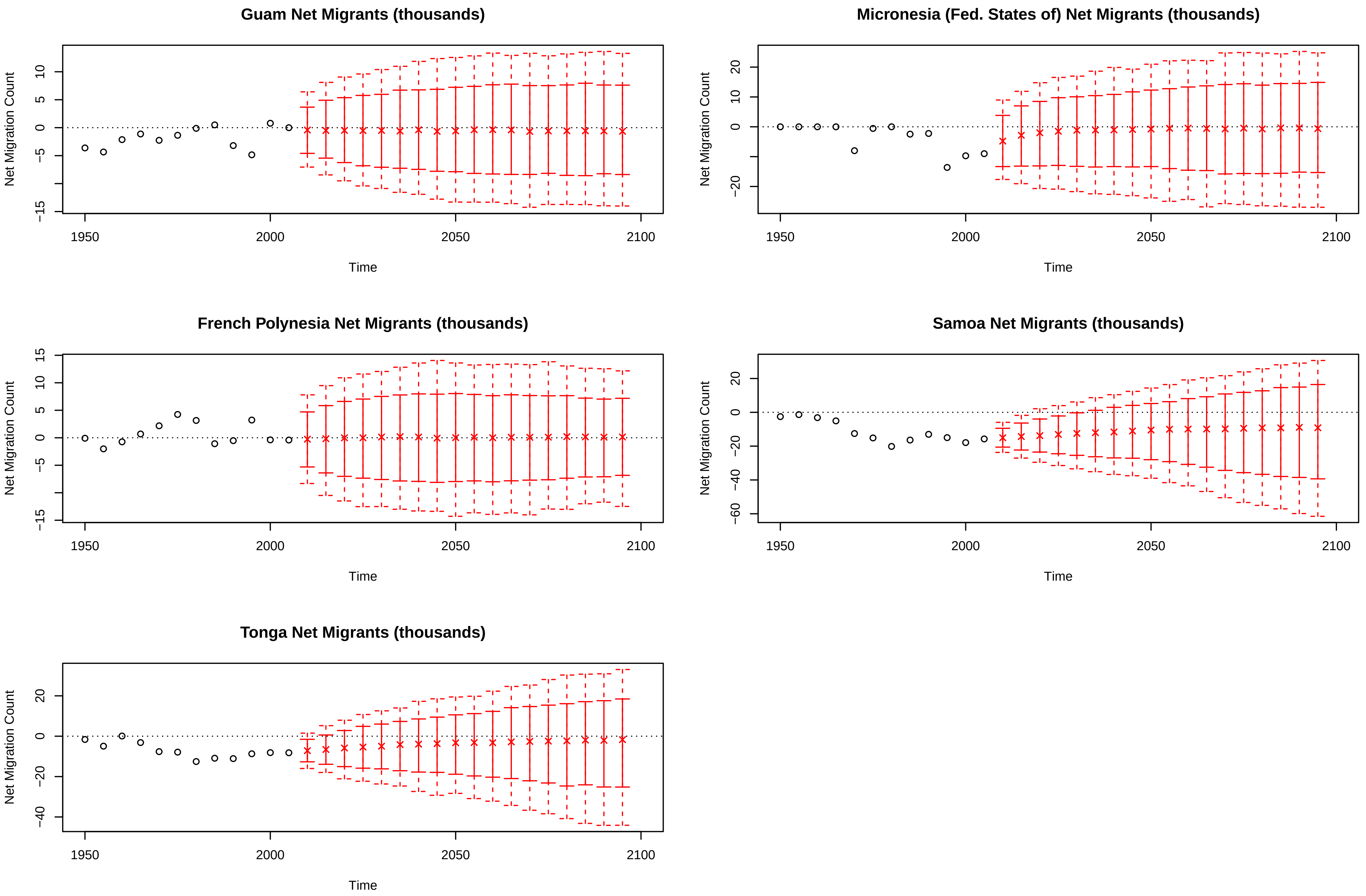
Guam Net Migrants (thousands)
Micronesia (Fed. States of) Net Migrants (thousands)
French Polynesia Net Migrants (thousands)
Samoa Net Migrants (thousands)
Tonga Net Migrants (thousands)
Net Migration Count
Time
1950
2000
2050
2100
