# Supplementary material for: Bayesian Probabilistic Projection of International Migration
Source: Demography. 2015 Sep 10;52(5):1627–50. doi: 10.1007/s13524-015-0415-0 (PMC4605963; doi:10.1007/s13524-015-0415-0)
Supplement: Supplementary file 2 — (PDF 421 KB) [file 13524_2015_415_MOESM2_ESM.pdf]

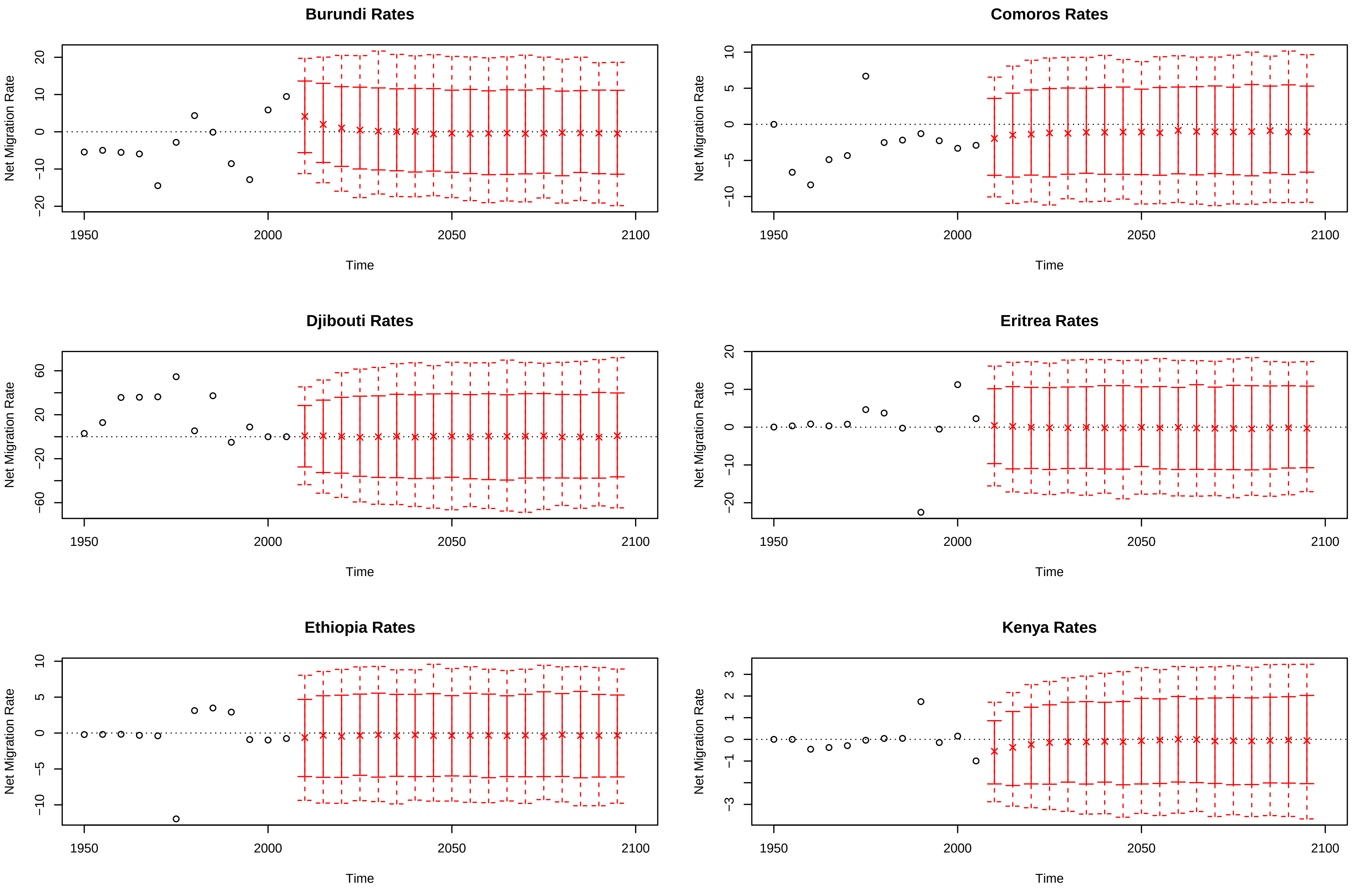
Burundi Rates
Comoros Rates
Djibouti Rates
Eritrea Rates
Ethiopia Rates
Kenya Rates
Net Migration Rate
Time
1950
2000
2050
2100

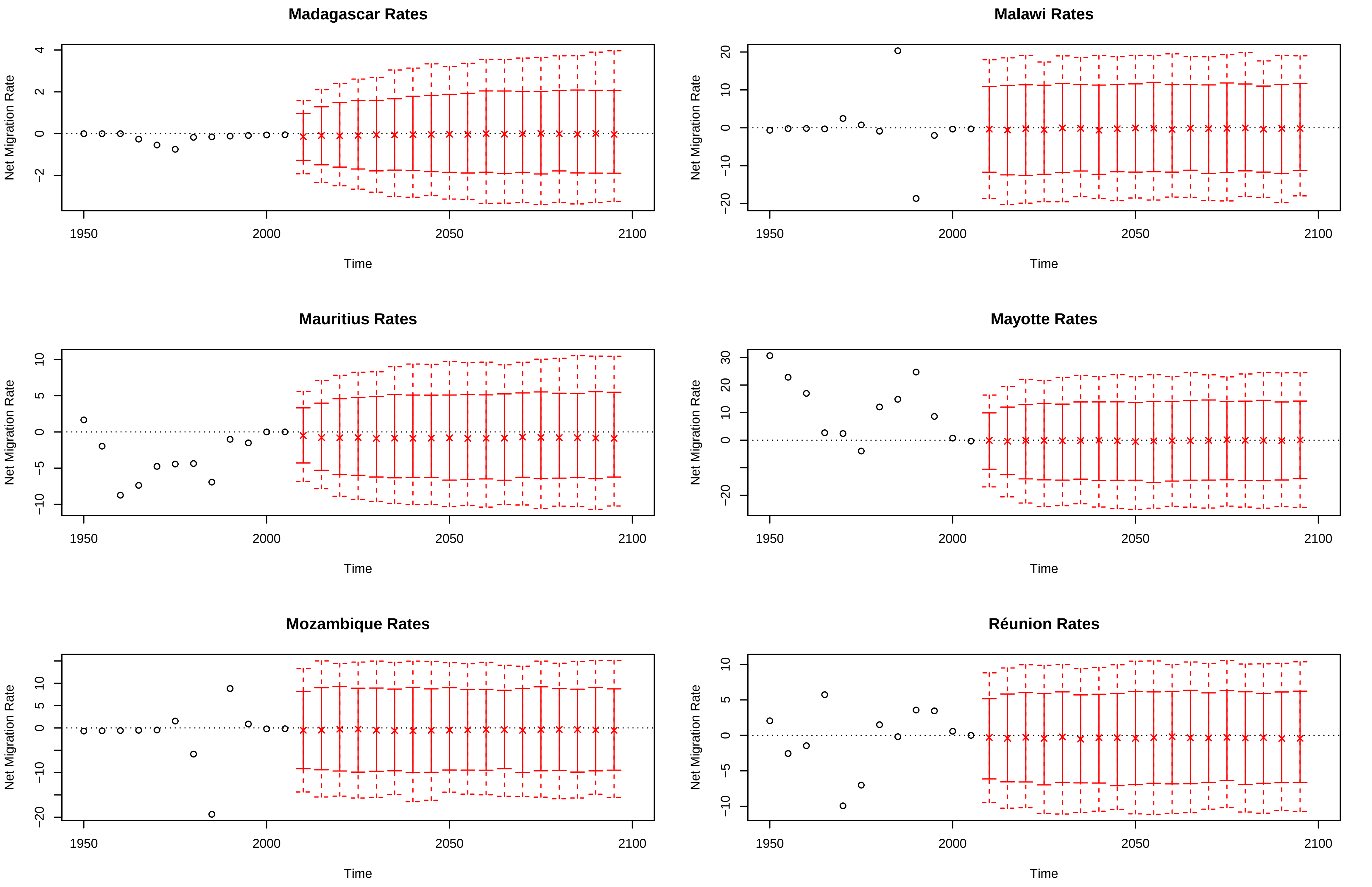
Madagascar Rates
Net Migration Rate
4
2
0
−2
Time
1950
2000
2050
2100
Malawi Rates
Net Migration Rate
20
10
0
−10
−20
Time
1950
2000
2050
2100
Mauritius Rates
Net Migration Rate
10
5
0
−5
−10
Time
1950
2000
2050
2100
Mayotte Rates
Net Migration Rate
30
20
10
0
−20
Time
1950
2000
2050
2100
Mozambique Rates
Net Migration Rate
10
5
0
−10
−20
Time
1950
2000
2050
2100
Réunion Rates
Net Migration Rate
10
5
0
−5
−10
Time
1950
2000
2050
2100

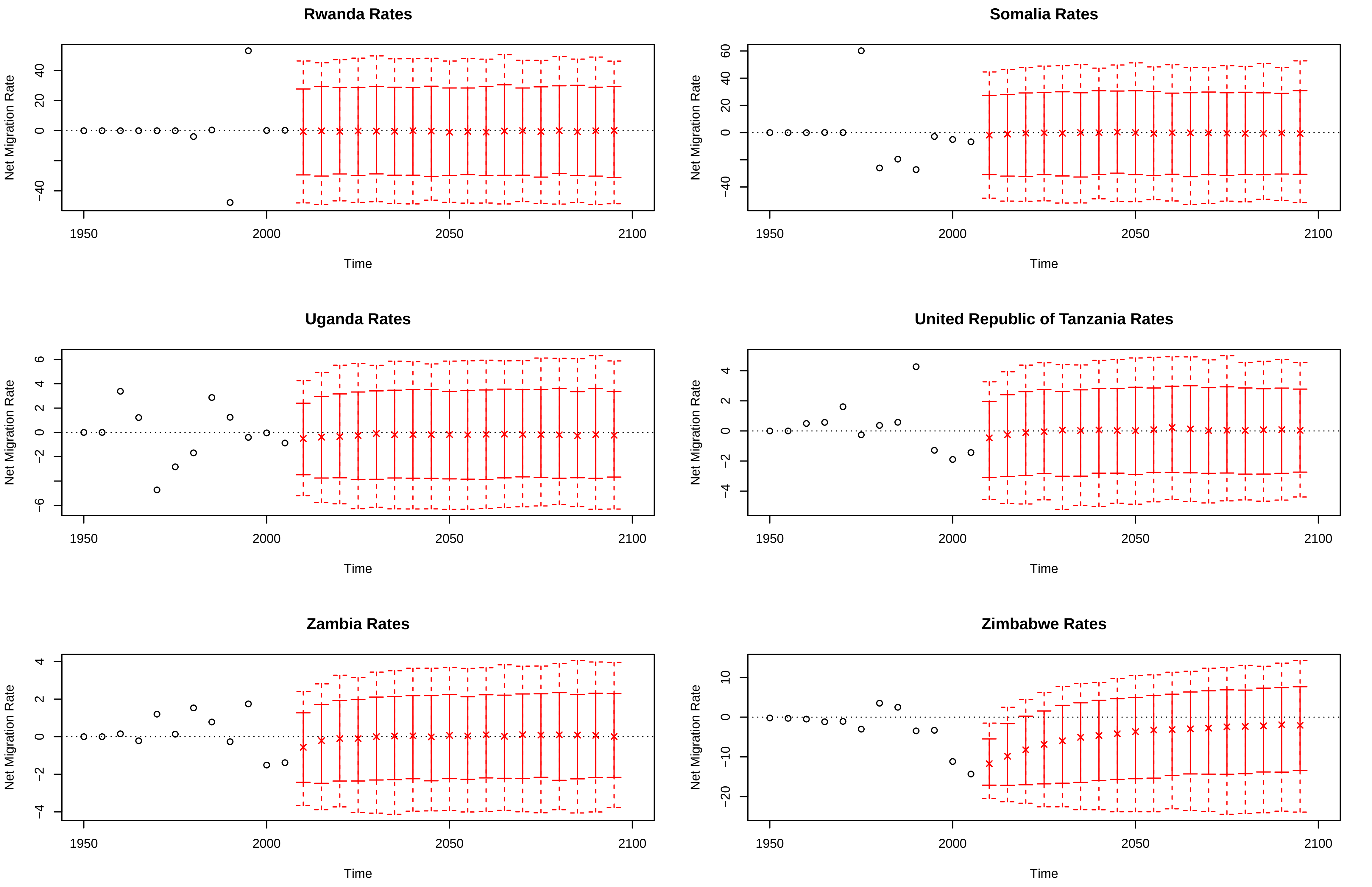
Rwanda Rates
Somalia Rates
Uganda Rates
United Republic of Tanzania Rates
Zambia Rates
Zimbabwe Rates
Net Migration Rate
Time
1950
2000
2050
2100
−40
0
20
40
60
−6
−2
2
4
6
−4
−20
−10
10

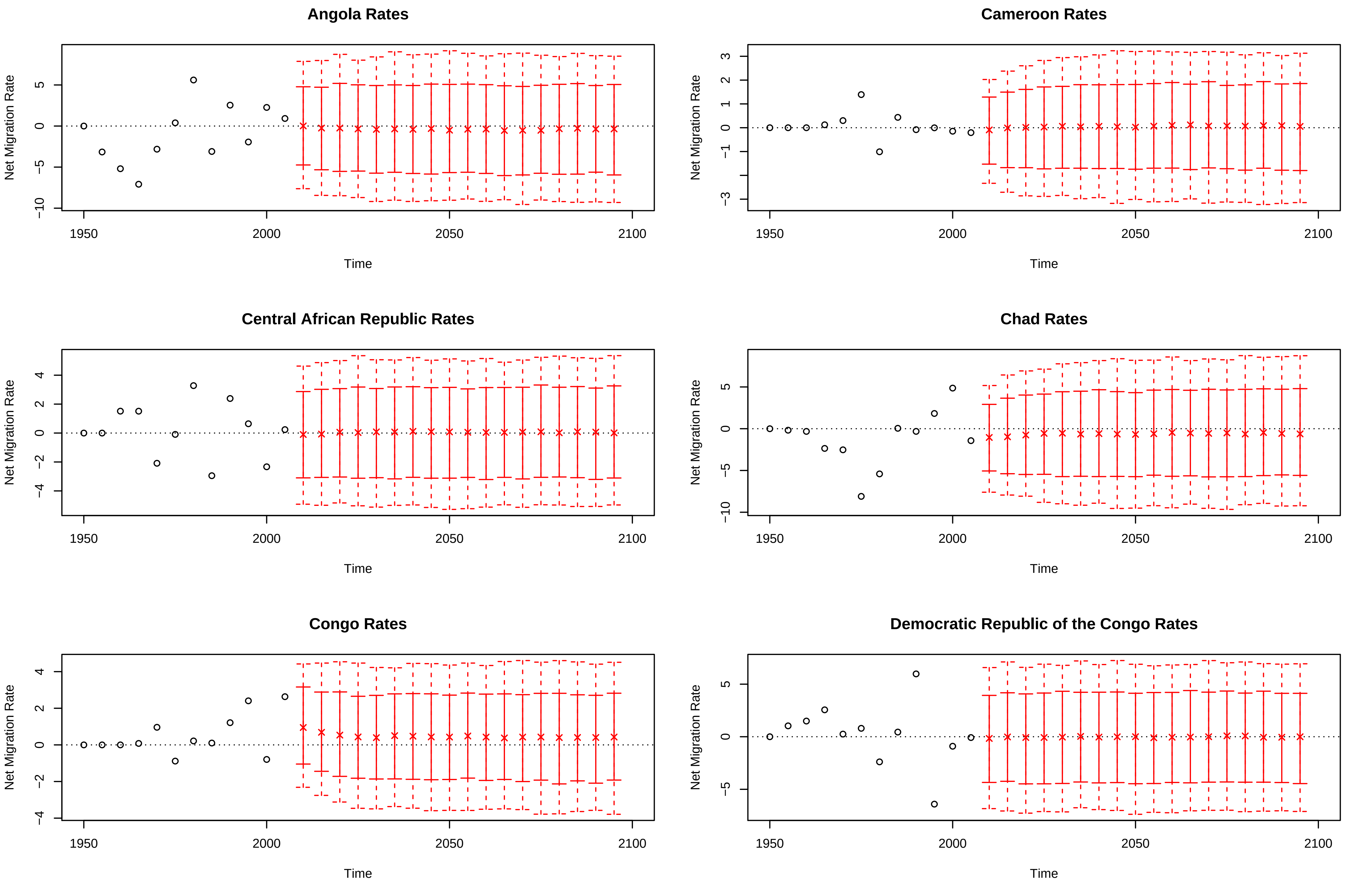

Angola Rates
Cameroon Rates
Central African Republic Rates
Chad Rates
Congo Rates
Democratic Republic of the Congo Rates
Net Migration Rate
Time
1950
2000
2050
2100

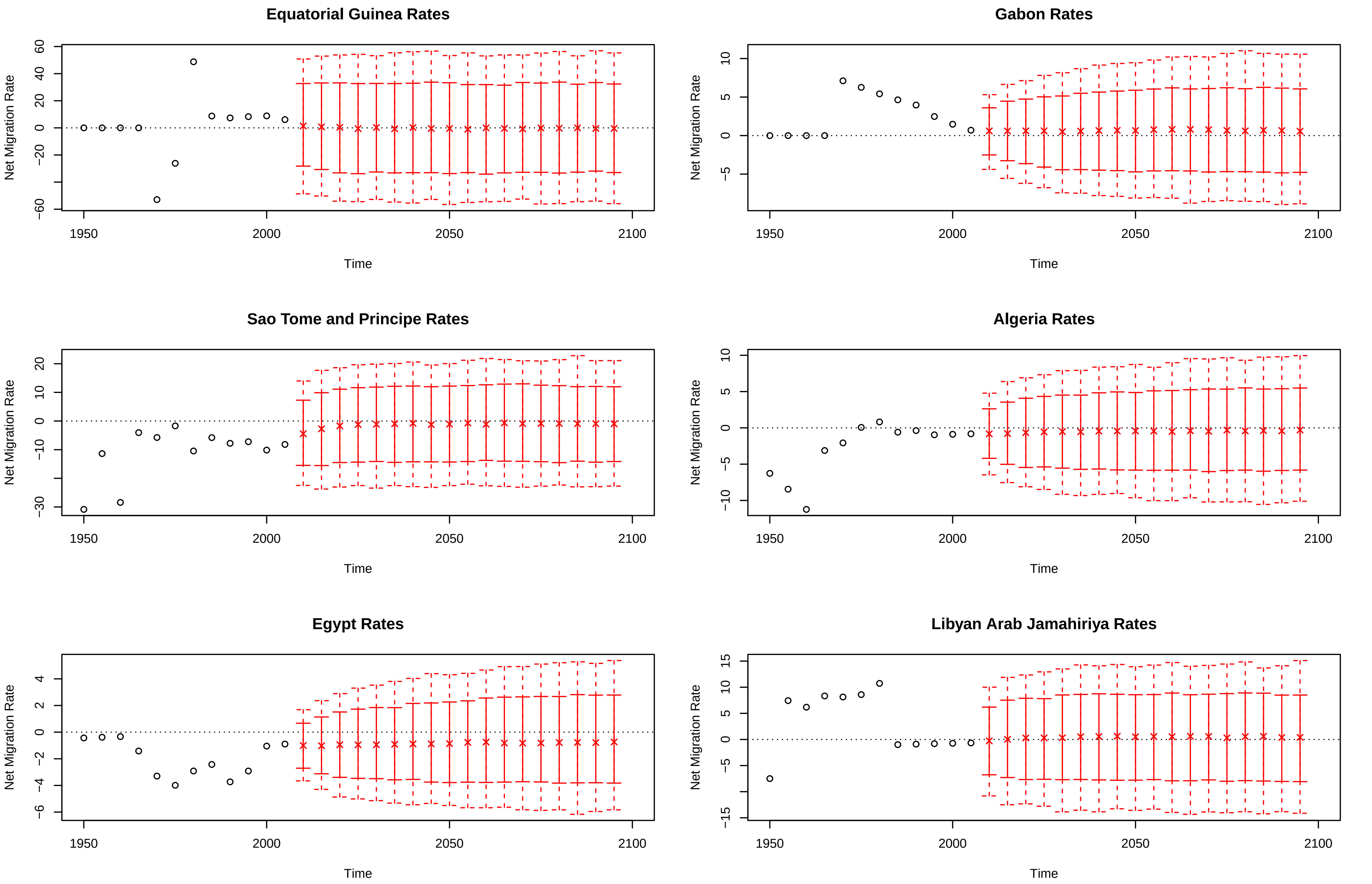
Equatorial Guinea Rates
Gabon Rates
Sao Tome and Principe Rates
Algeria Rates
Egypt Rates
Libyan Arab Jamahiriya Rates
Net Migration Rate
Time
1950
2000
2050
2100
60
40
20
0
−20
−60
10
5
−5
−10
−30
4
2
−2
−4
−6
15
−15

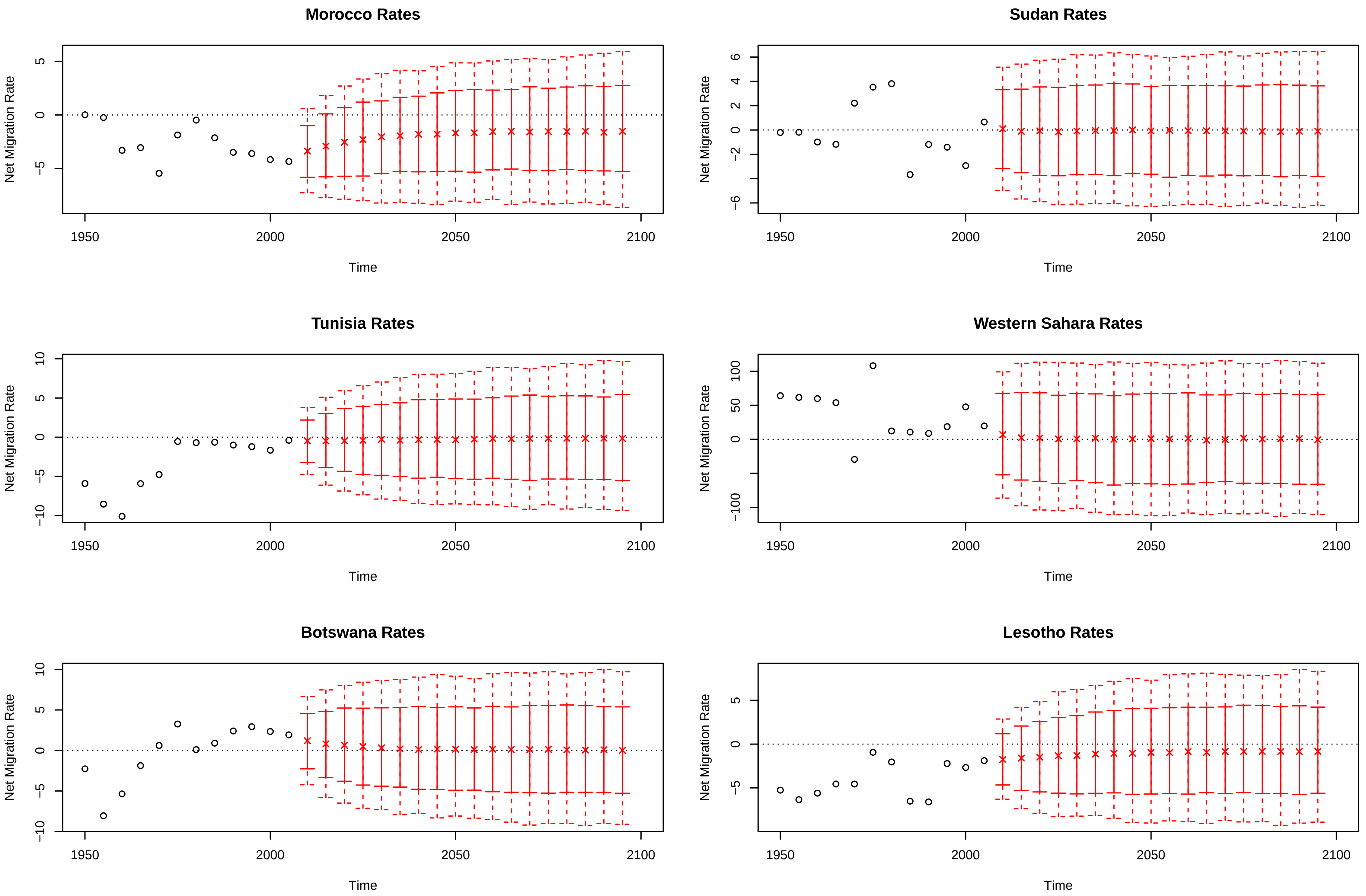
Morocco Rates
Sudan Rates
Tunisia Rates
Western Sahara Rates
Botswana Rates
Lesotho Rates
Net Migration Rate
Time
1950
2000
2050
2100

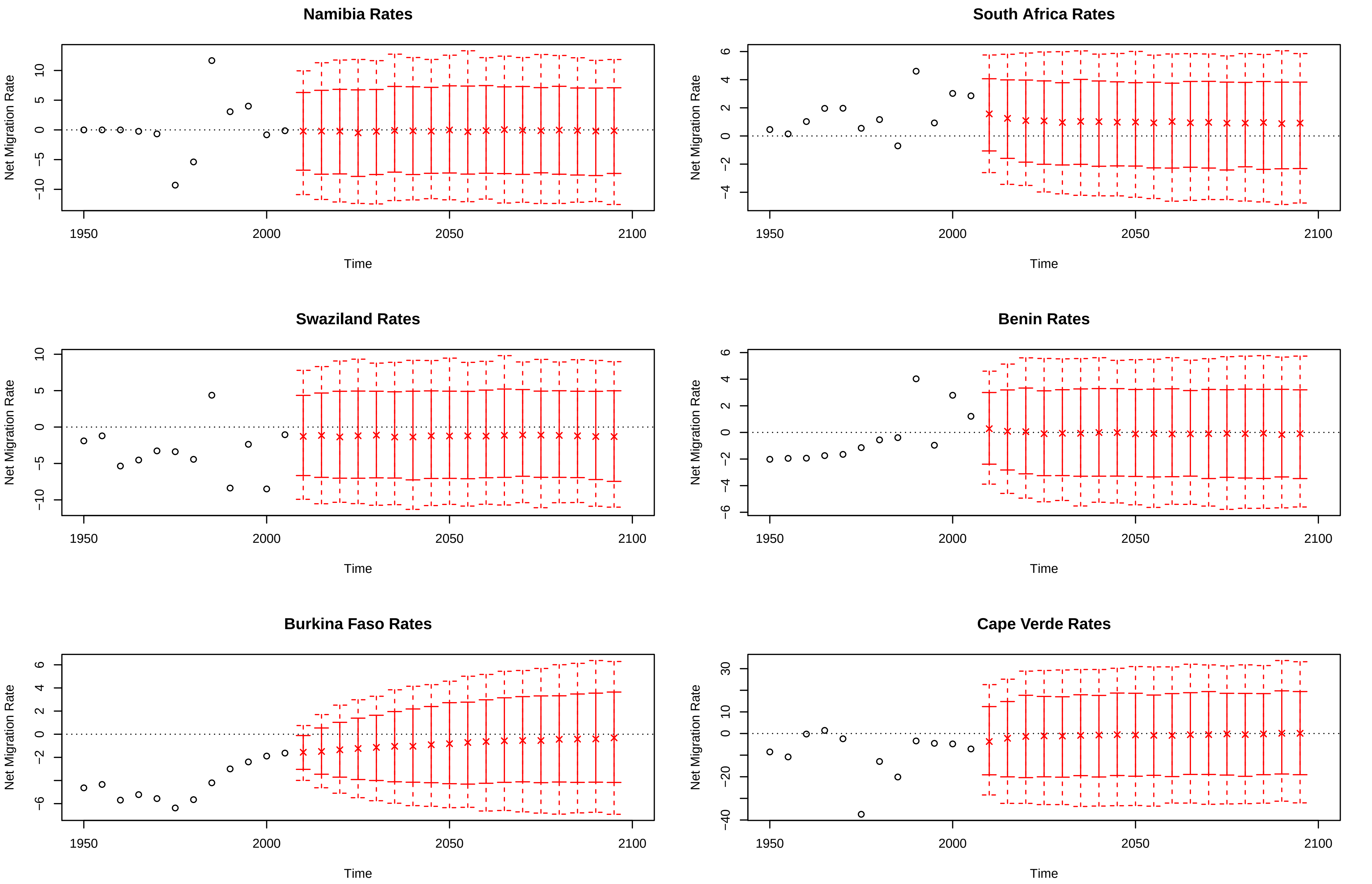
Namibia Rates
South Africa Rates
Swaziland Rates
Benin Rates
Burkina Faso Rates
Cape Verde Rates
Net Migration Rate
Time
1950
2000
2050
2100

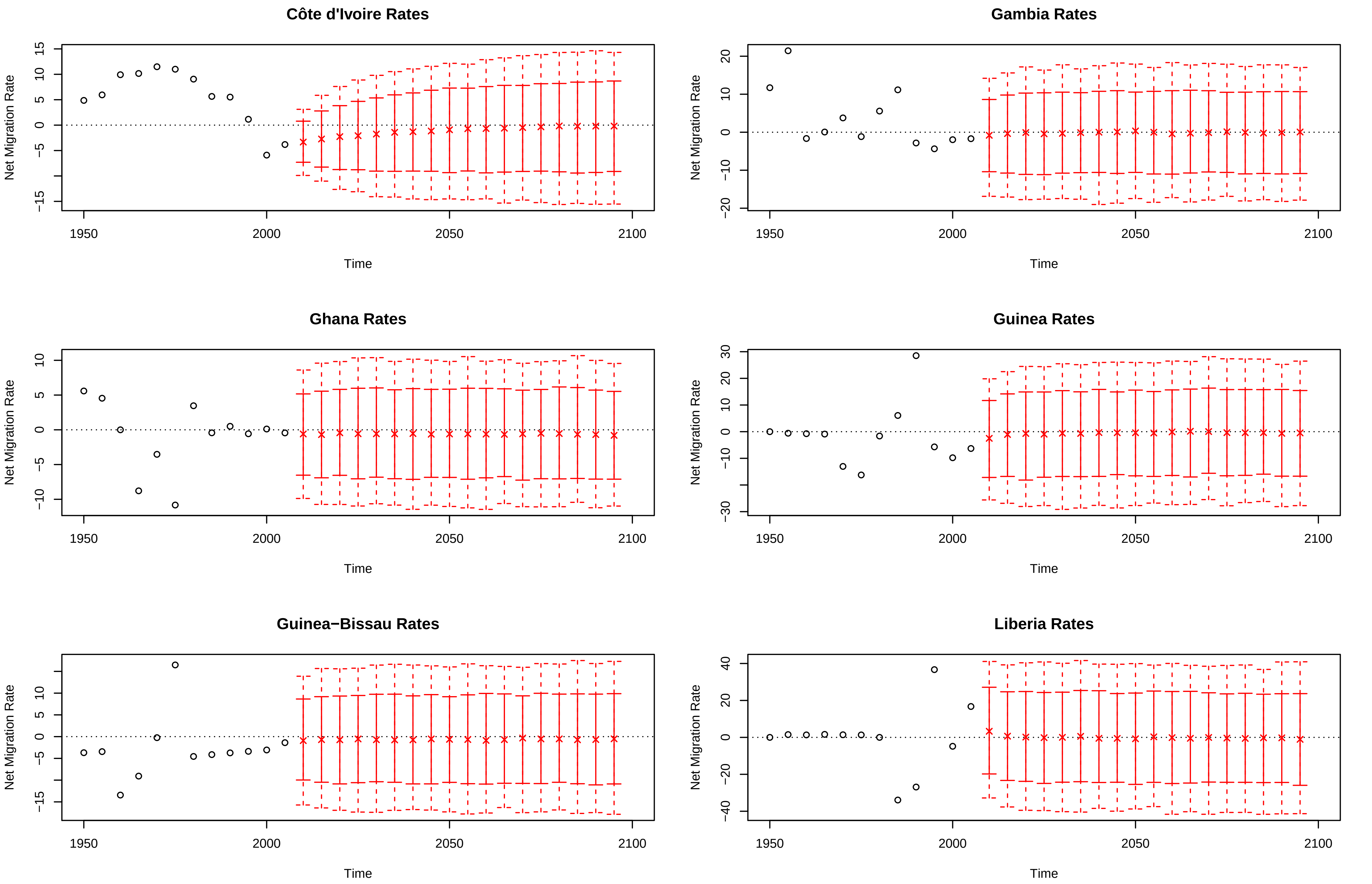
Côte d'Ivoire Rates
Gambia Rates
Ghana Rates
Guinea Rates
Guinea−Bissau Rates
Liberia Rates
Net Migration Rate
Time
1950
2000
2050
2100

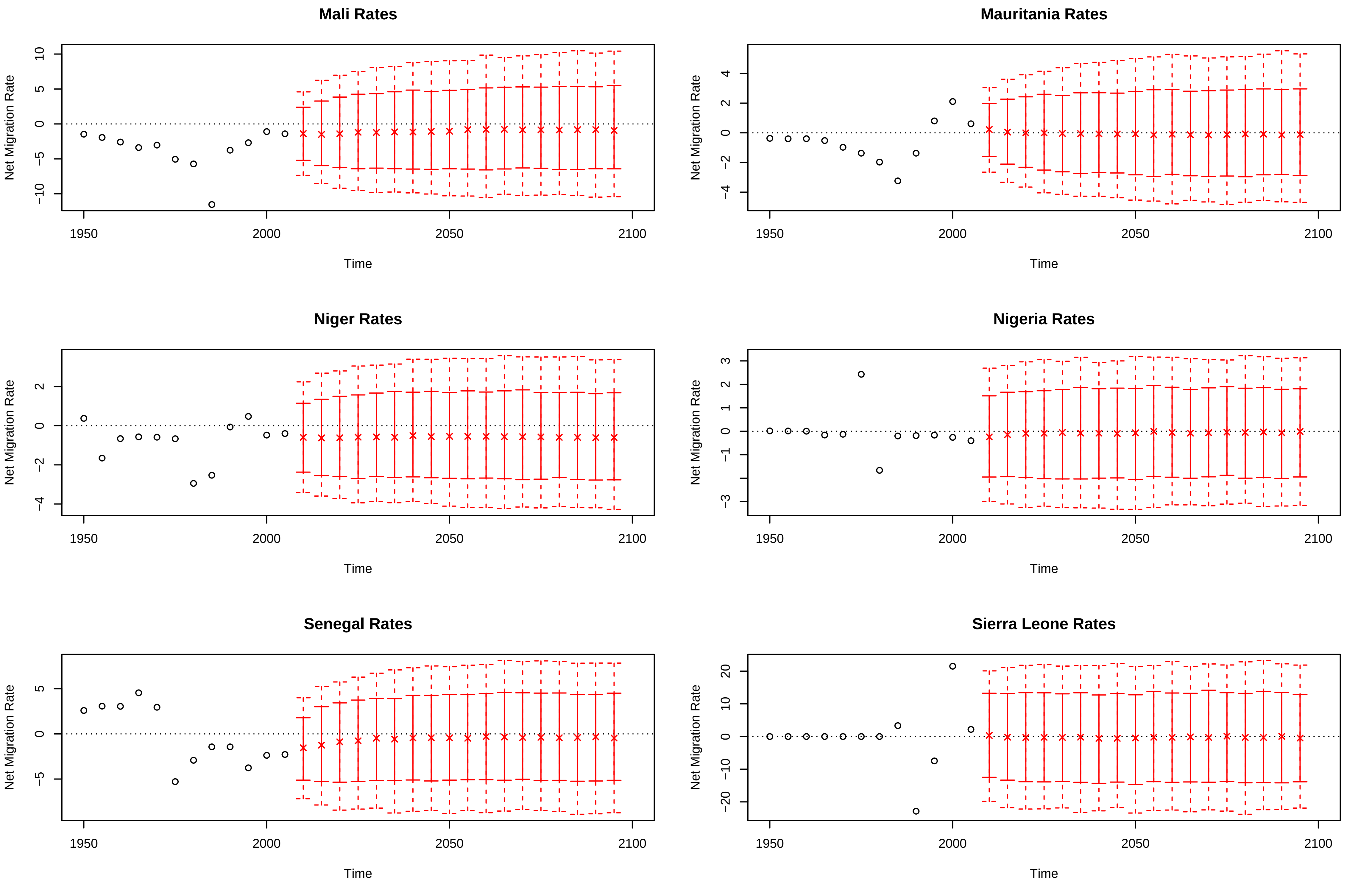
Mali Rates
Net Migration Rate
10
5
0
−5
−10
1950
2000
2050
2100
Time
Mauritania Rates
Net Migration Rate
4
2
0
−2
−4
1950
2000
2050
2100
Time
Niger Rates
Net Migration Rate
2
0
−2
−4
1950
2000
2050
2100
Time
Nigeria Rates
Net Migration Rate
3
2
1
0
−1
−3
1950
2000
2050
2100
Time
Senegal Rates
Net Migration Rate
5
0
−5
1950
2000
2050
2100
Time
Sierra Leone Rates
Net Migration Rate
20
10
0
−10
−20
1950
2000
2050
2100
Time

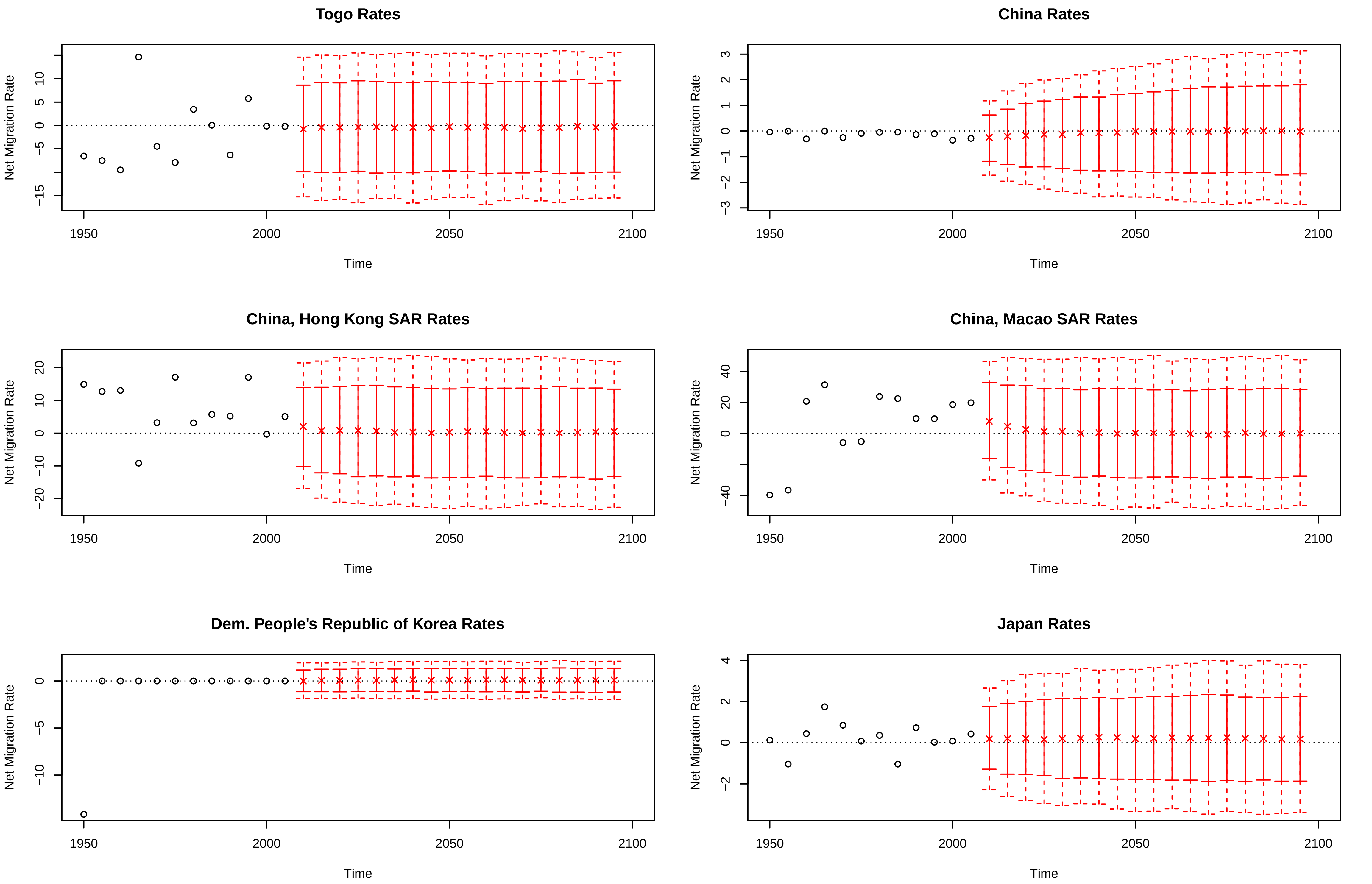

Togo Rates
China Rates
China, Hong Kong SAR Rates
China, Macao SAR Rates
Dem. People's Republic of Korea Rates
Japan Rates
Net Migration Rate
Time
1950
2000
2050
2100

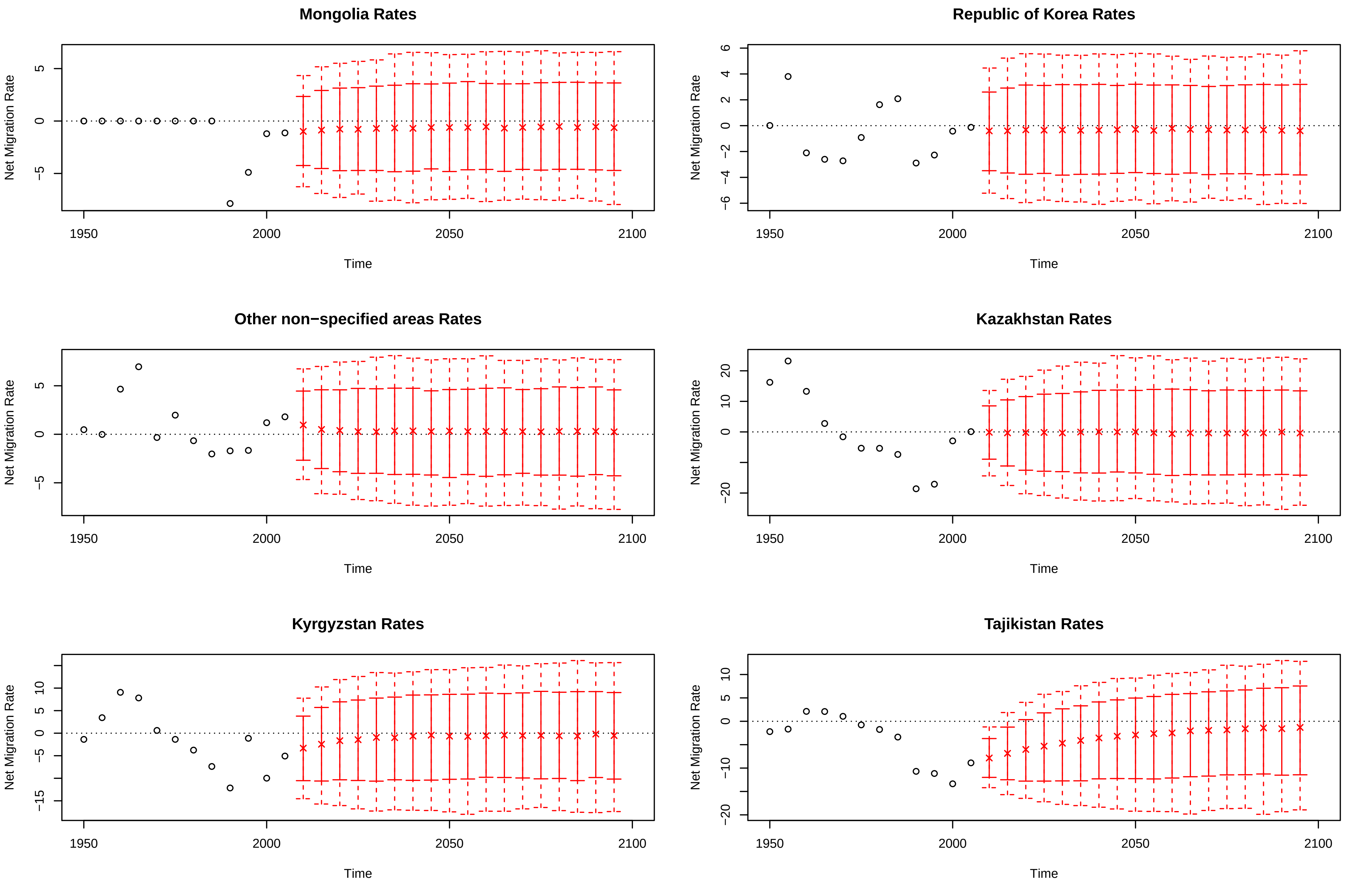
Mongolia Rates
Republic of Korea Rates
Other non−specified areas Rates
Kazakhstan Rates
Kyrgyzstan Rates
Tajikistan Rates
Net Migration Rate
Time
1950
2000
2050
2100

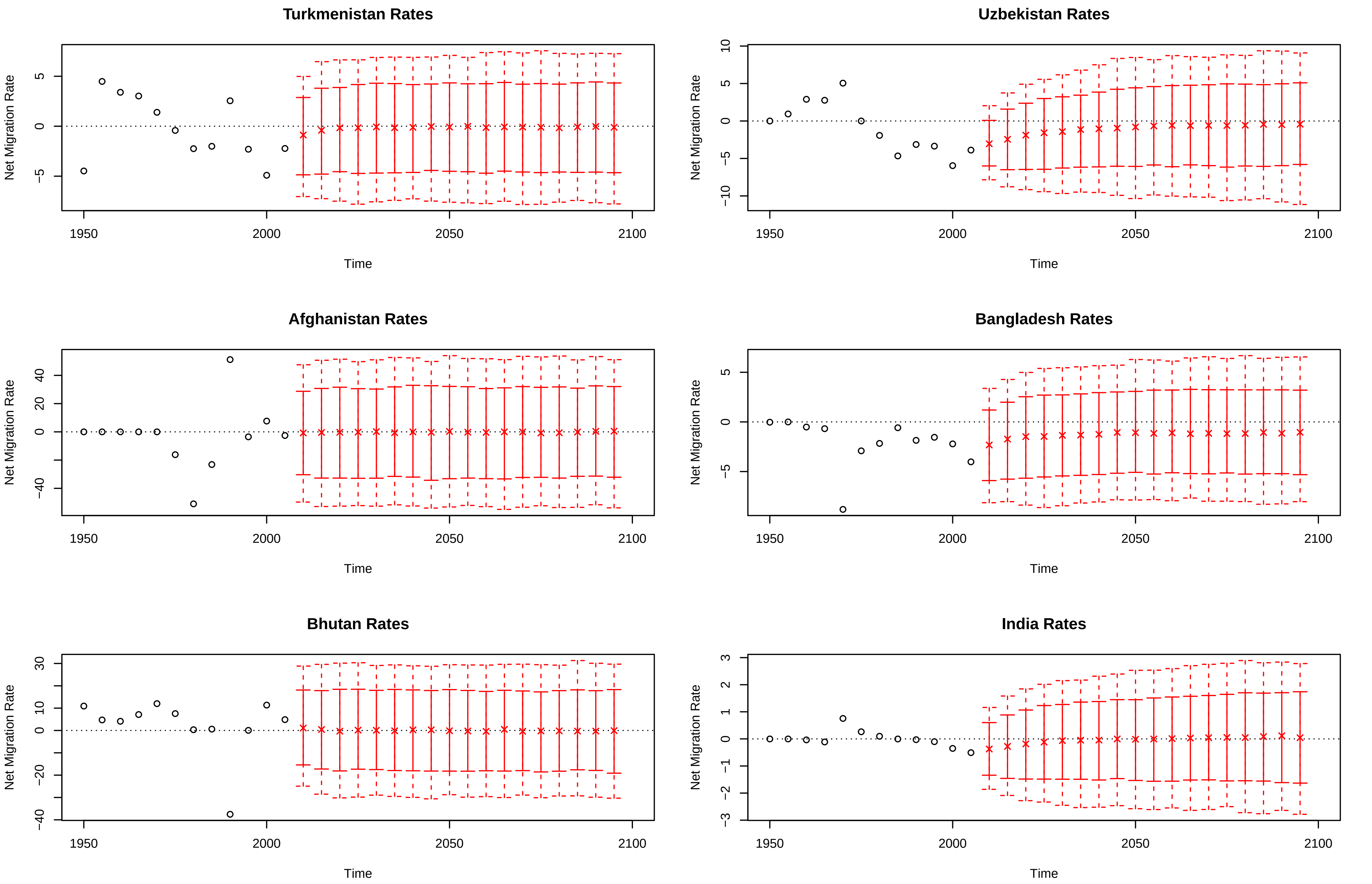

Turkmenistan Rates
Net Migration Rate
−5
0
5
1950
2000
2050
2100
Time
Uzbekistan Rates
Net Migration Rate
−10
−5
0
5
10
1950
2000
2050
2100
Time
Afghanistan Rates
Net Migration Rate
−40
0
20
40
1950
2000
2050
2100
Time
Bangladesh Rates
Net Migration Rate
−5
0
5
1950
2000
2050
2100
Time
Bhutan Rates
Net Migration Rate
−40
−20
0
10
30
1950
2000
2050
2100
Time
India Rates
Net Migration Rate
−3
−2
−1
0
1
2
3
1950
2000
2050
2100
Time

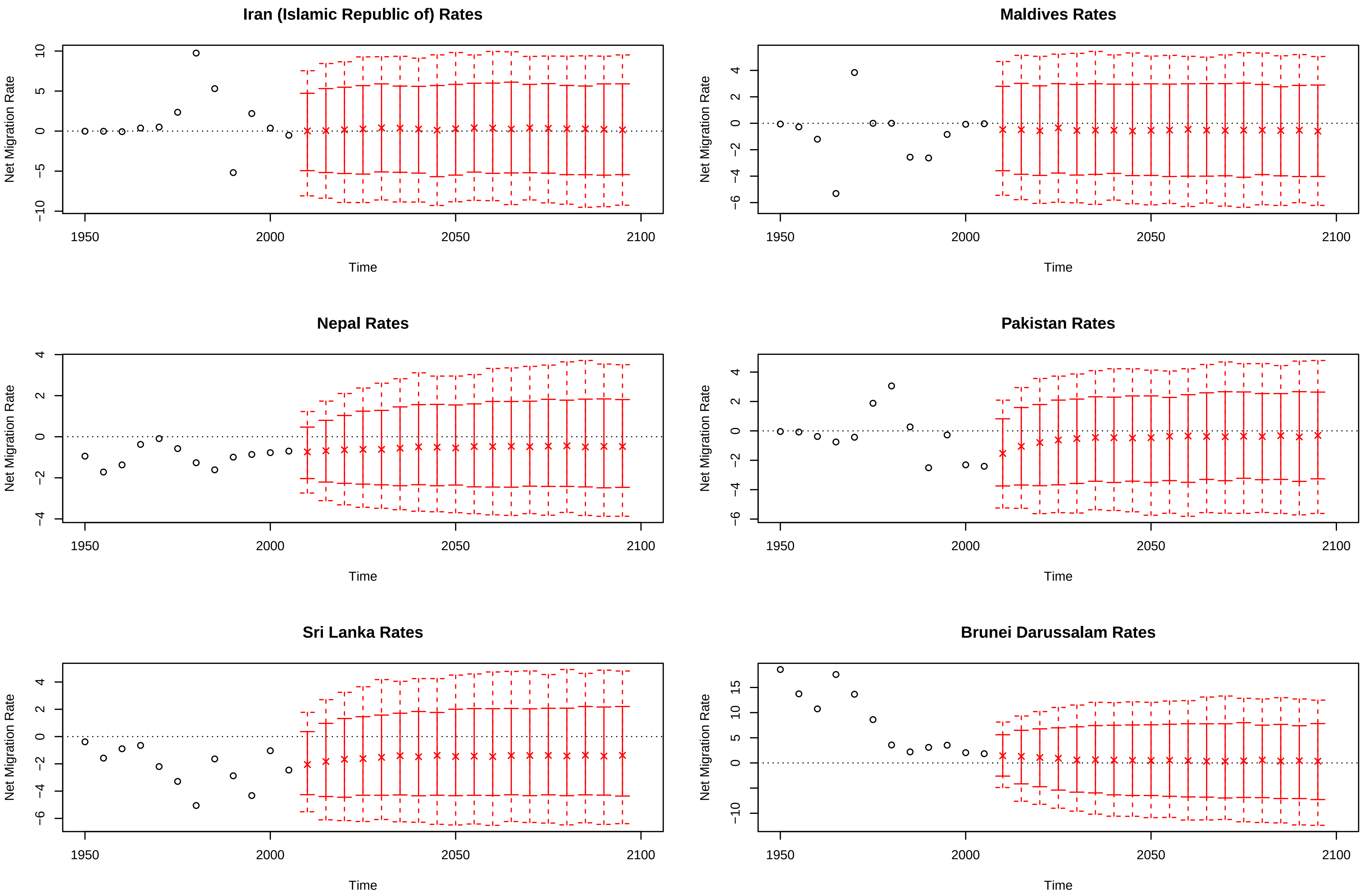
Iran (Islamic Republic of) Rates
Net Migration Rate
−10
−5
0
5
10
1950
2000
2050
2100
Time
Maldives Rates
Net Migration Rate
−6
−4
−2
0
2
4
1950
2000
2050
2100
Time
Nepal Rates
Net Migration Rate
−4
−2
0
2
4
1950
2000
2050
2100
Time
Pakistan Rates
Net Migration Rate
−6
−4
−2
0
2
4
1950
2000
2050
2100
Time
Sri Lanka Rates
Net Migration Rate
−6
−4
−2
0
2
4
1950
2000
2050
2100
Time
Brunei Darussalam Rates
Net Migration Rate
−10
0
5
10
15
1950
2000
2050
2100
Time

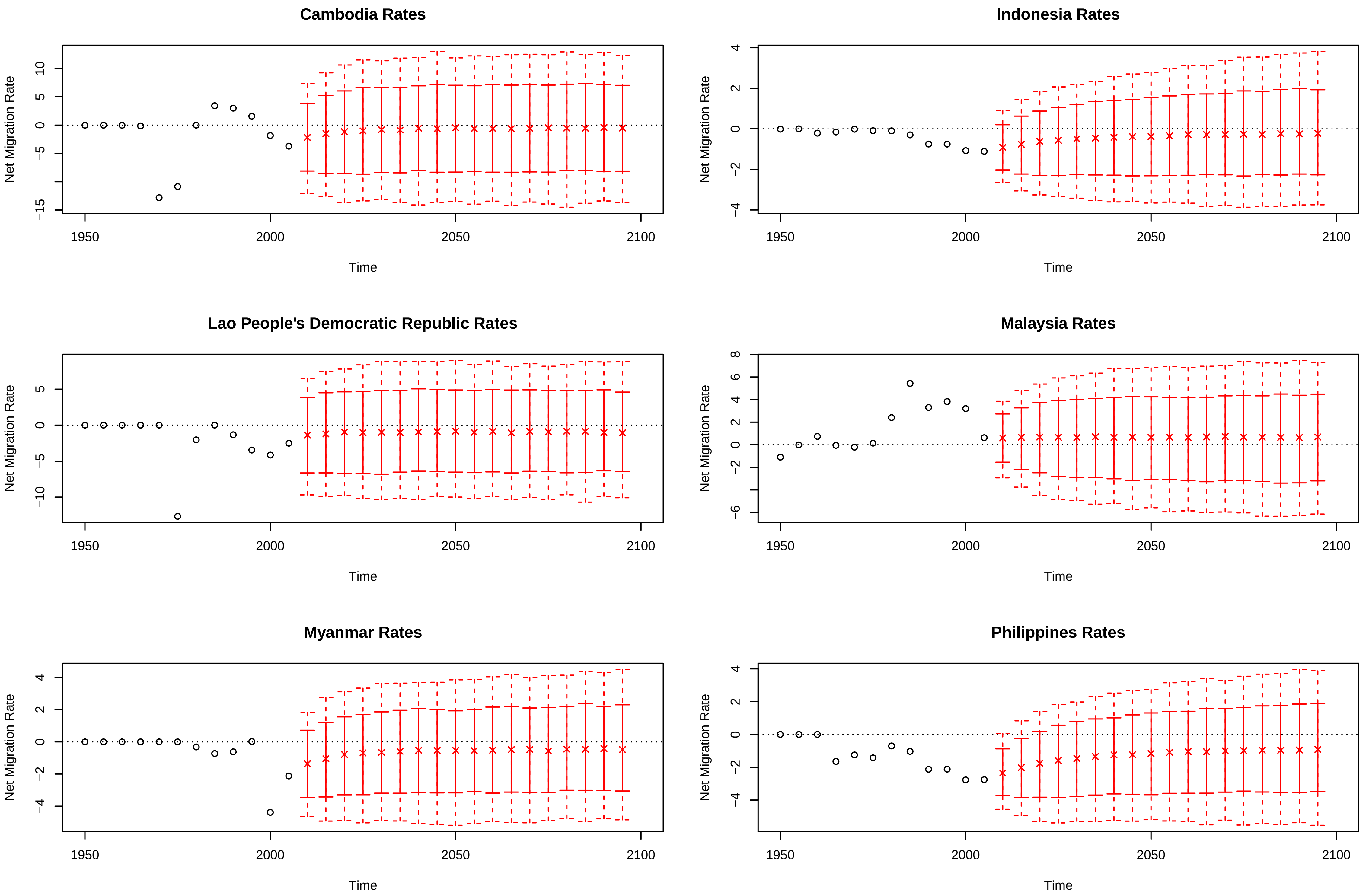

Cambodia Rates
Net Migration Rate
10
5
0
−5
−15
1950
2000
2050
2100
Time
Indonesia Rates
Net Migration Rate
4
2
0
−2
−4
1950
2000
2050
2100
Time
Lao People's Democratic Republic Rates
Net Migration Rate
5
0
−5
−10
1950
2000
2050
2100
Time
Malaysia Rates
Net Migration Rate
8
6
4
2
0
−2
−6
1950
2000
2050
2100
Time
Myanmar Rates
Net Migration Rate
4
2
0
−2
−4
1950
2000
2050
2100
Time
Philippines Rates
Net Migration Rate
4
2
0
−2
−4
1950
2000
2050
2100
Time

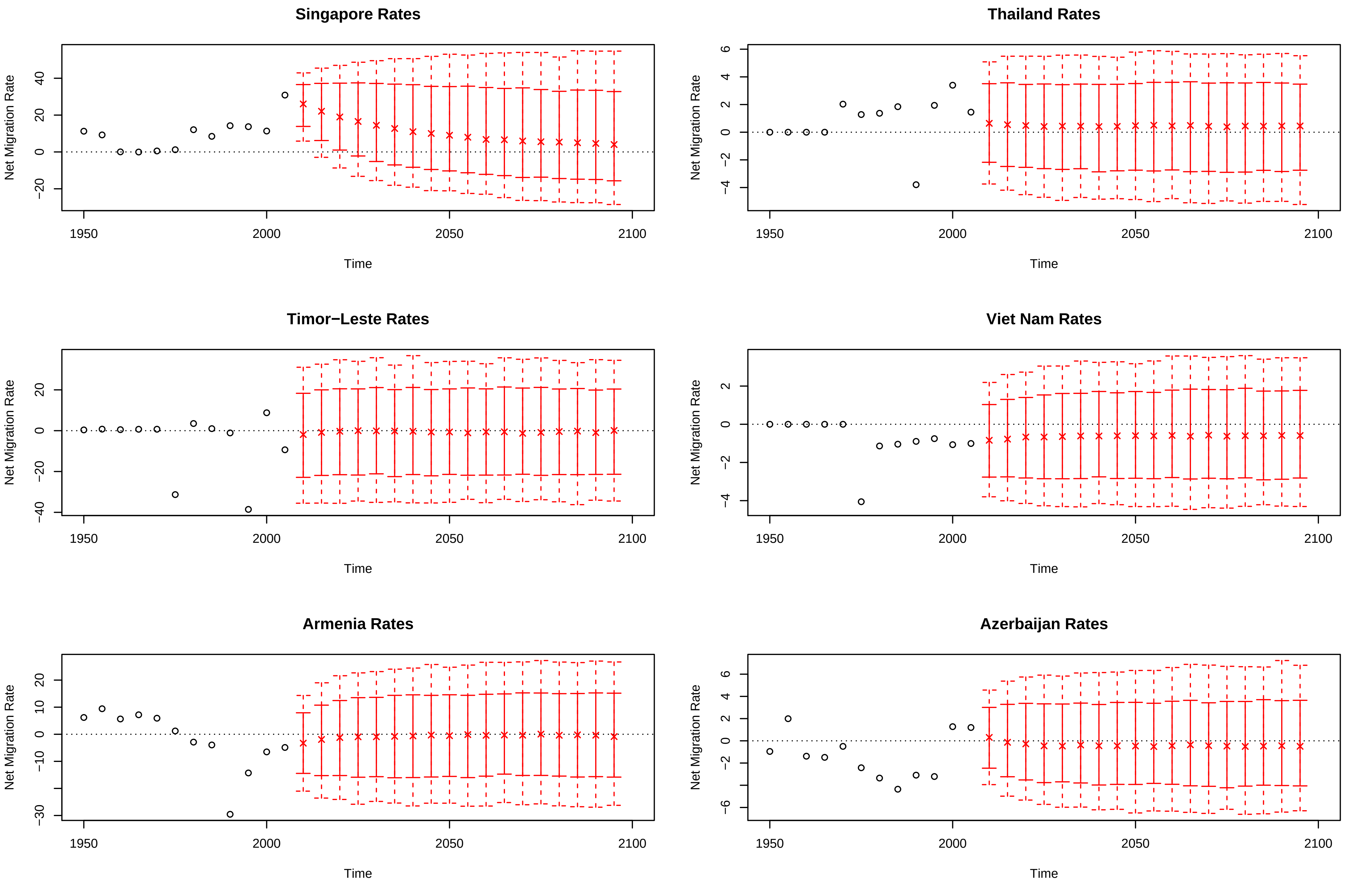
Singapore Rates
Net Migration Rate
−20
0
20
40
1950
2000
2050
2100
Time
Thailand Rates
Net Migration Rate
−4
−2
0
2
4
6
1950
2000
2050
2100
Time
Timor−Leste Rates
Net Migration Rate
−40
−20
0
20
1950
2000
2050
2100
Time
Viet Nam Rates
Net Migration Rate
−4
−2
0
2
1950
2000
2050
2100
Time
Armenia Rates
Net Migration Rate
−30
−10
0
10
20
1950
2000
2050
2100
Time
Azerbaijan Rates
Net Migration Rate
−6
−2
0
2
4
6
1950
2000
2050
2100
Time

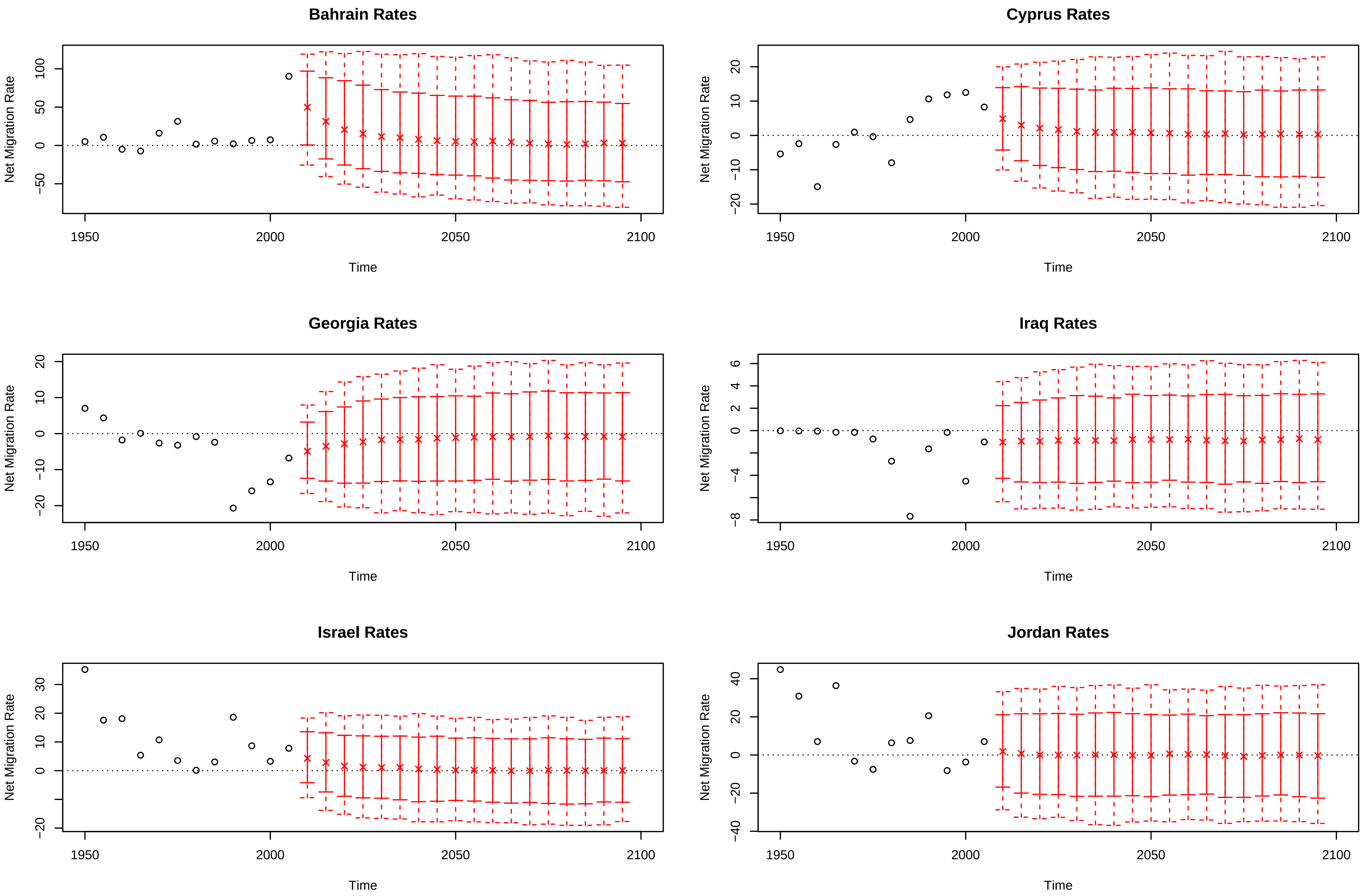
Bahrain Rates
Cyprus Rates
Georgia Rates
Iraq Rates
Israel Rates
Jordan Rates
Net Migration Rate
Time
1950
2000
2050
2100

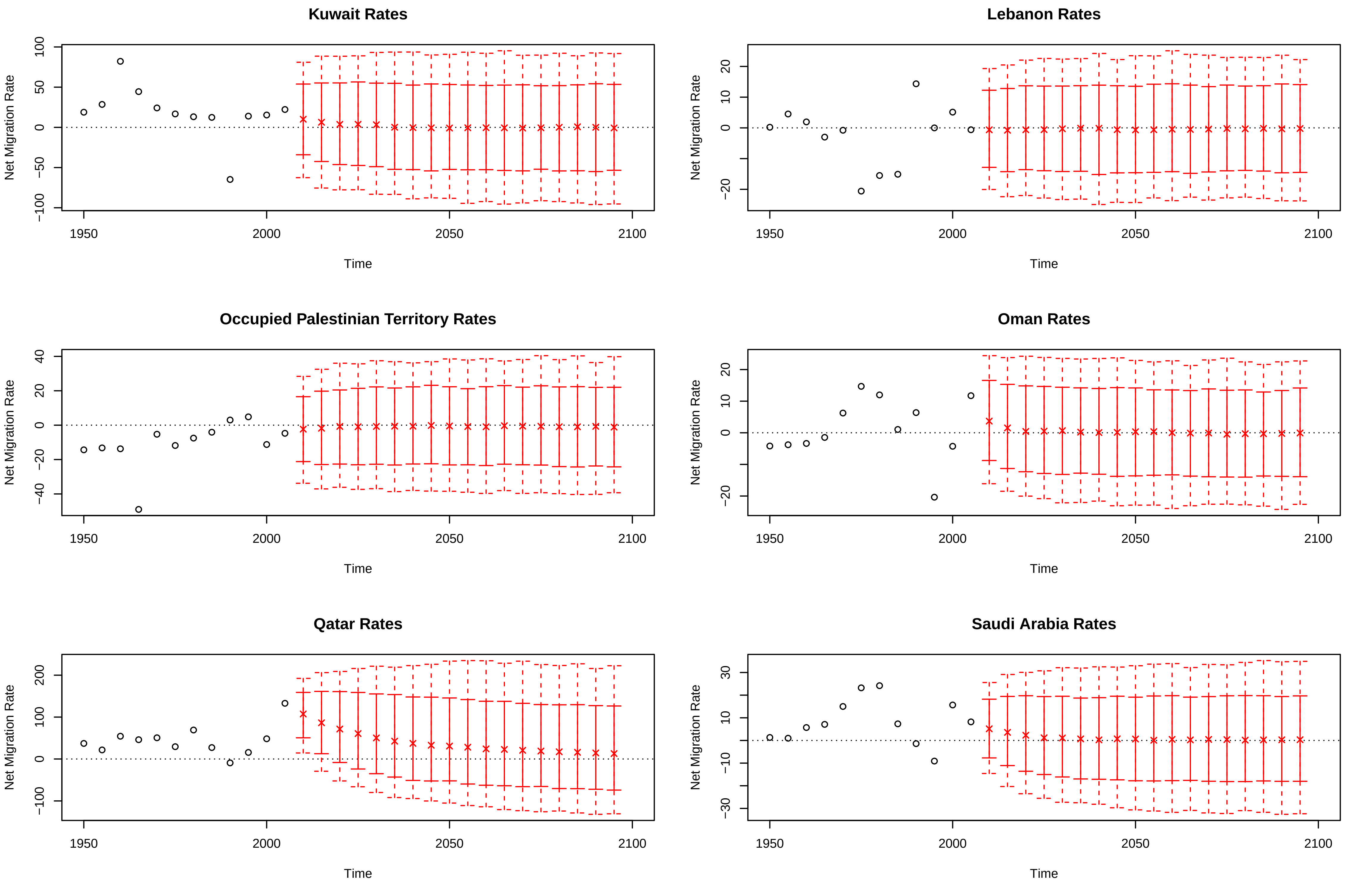
Kuwait Rates
Lebanon Rates
Occupied Palestinian Territory Rates
Oman Rates
Qatar Rates
Saudi Arabia Rates
Net Migration Rate
Time
1950
2000
2050
2100

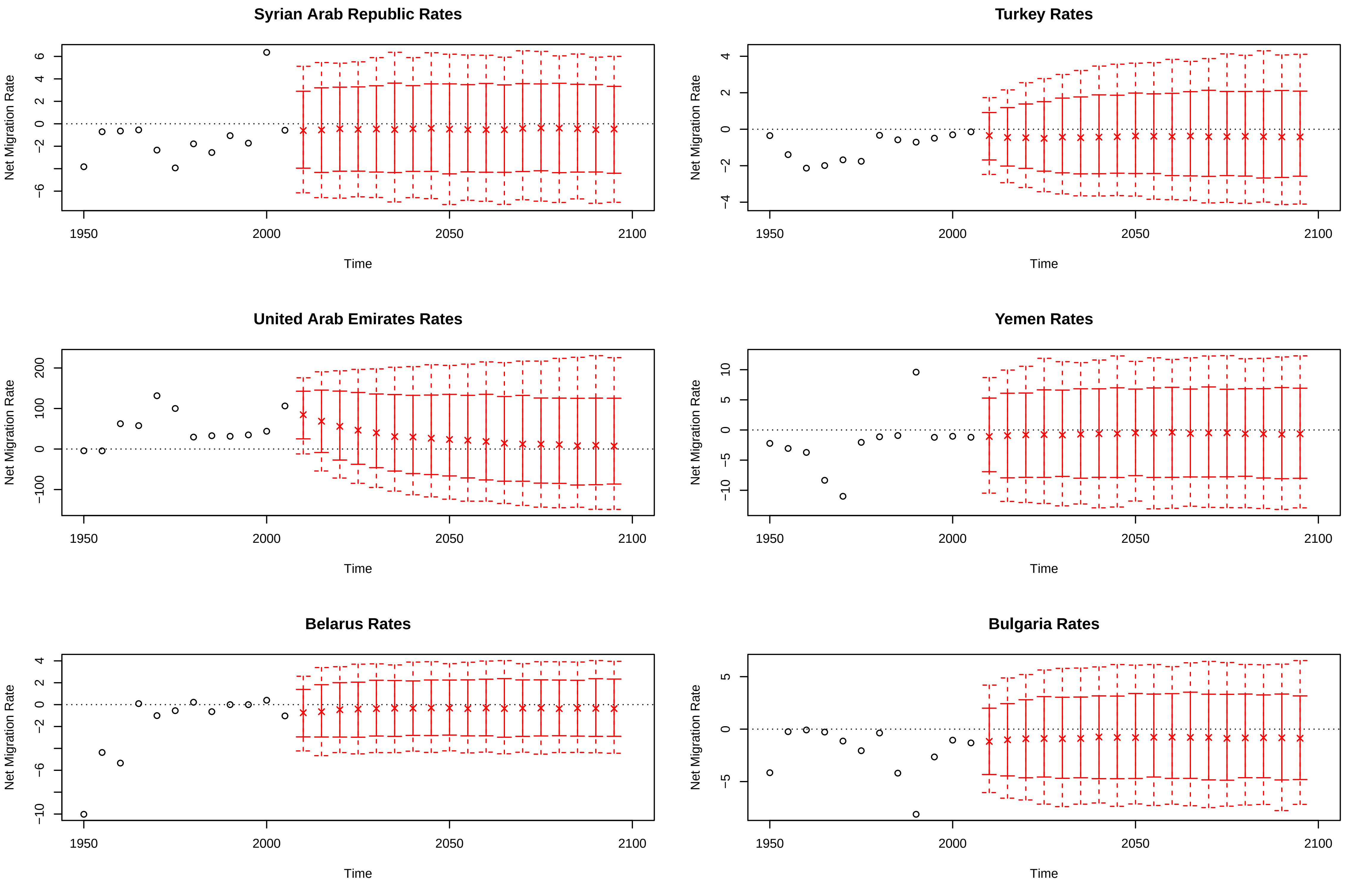

Syrian Arab Republic Rates
Turkey Rates
United Arab Emirates Rates
Yemen Rates
Belarus Rates
Bulgaria Rates
Net Migration Rate
Time
1950
2000
2050
2100

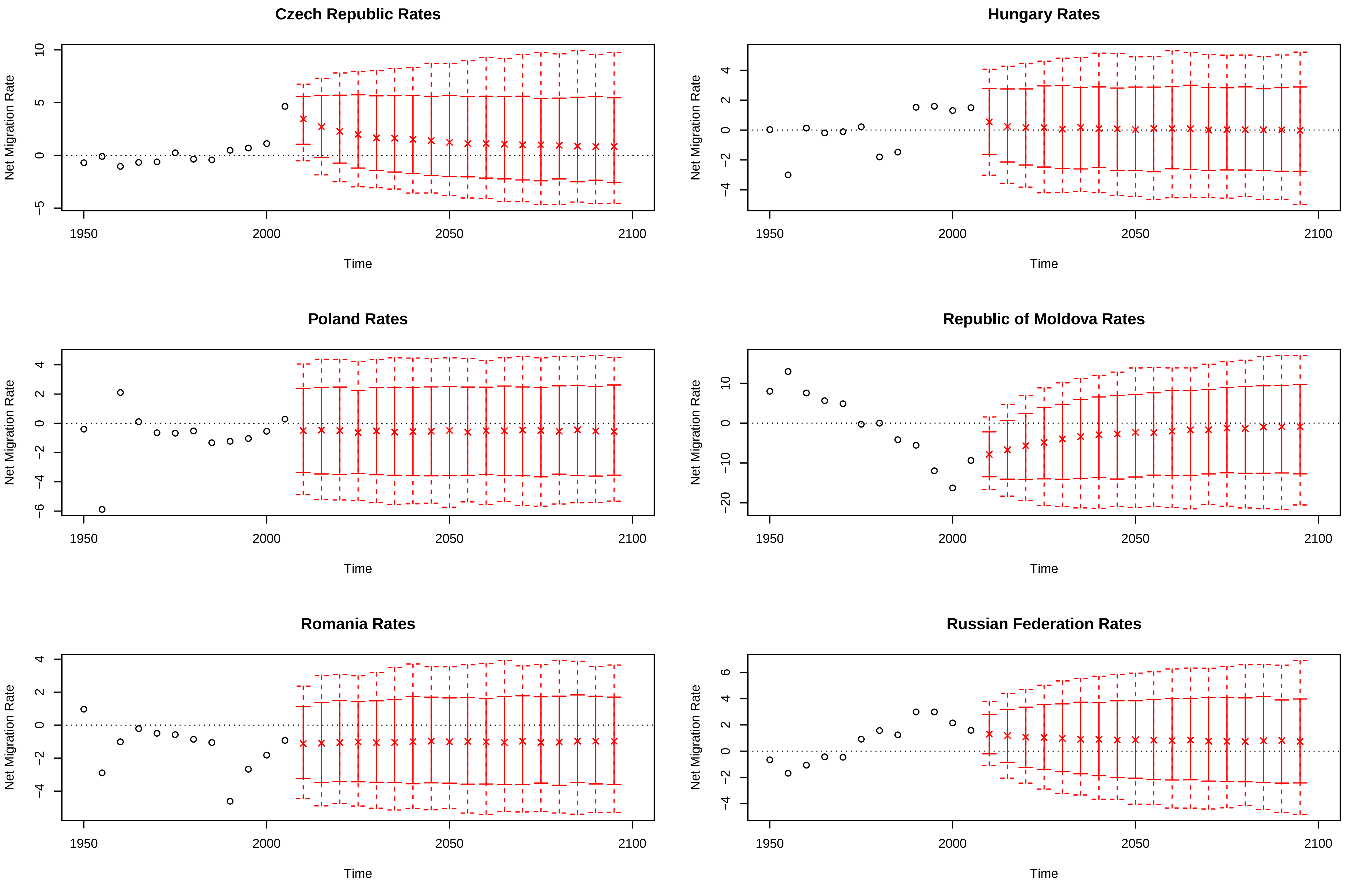
Czech Republic Rates
Hungary Rates
Poland Rates
Republic of Moldova Rates
Romania Rates
Russian Federation Rates
Net Migration Rate
Time
1950
2000
2050
2100

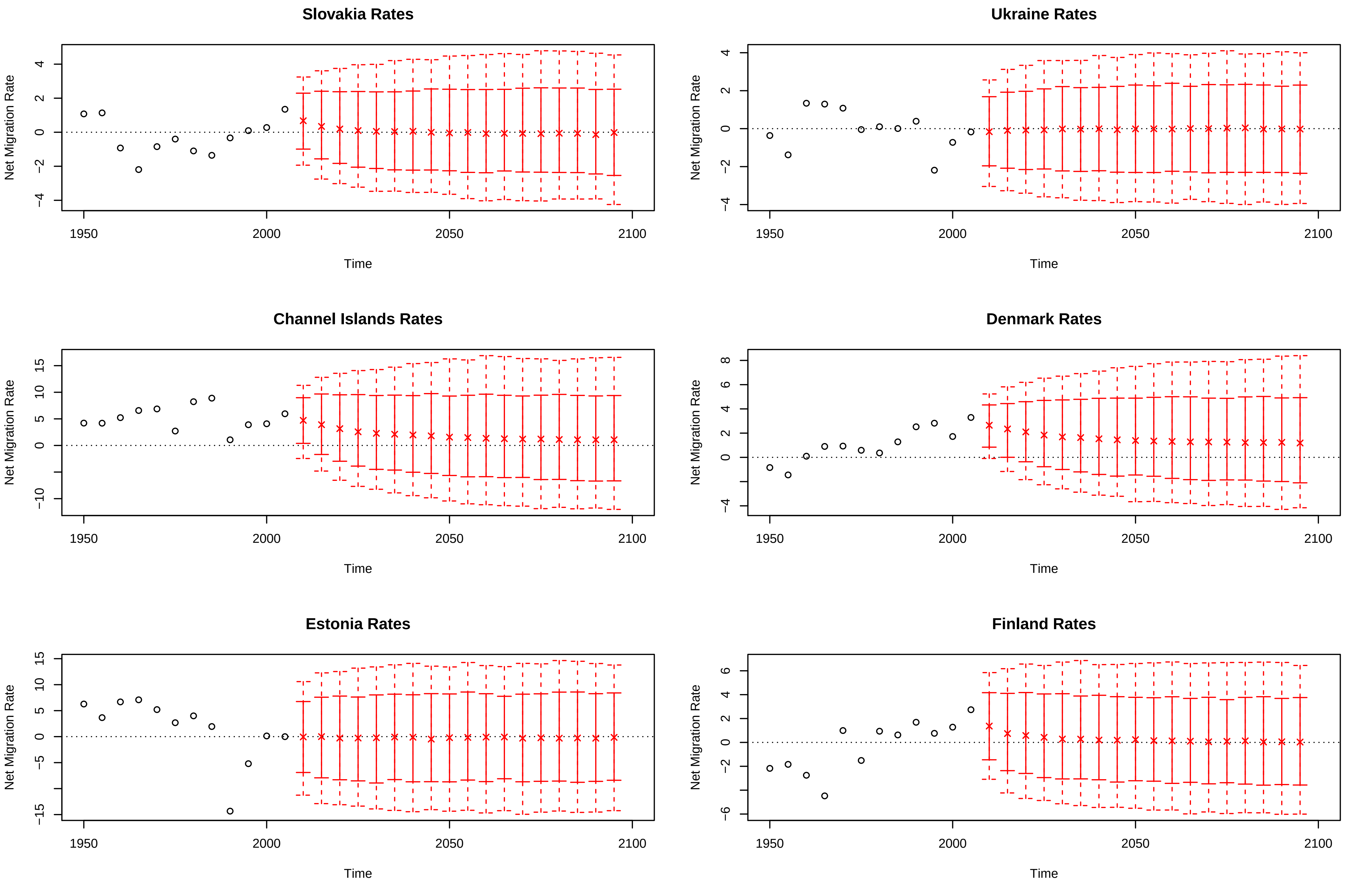

Slovakia Rates
Ukraine Rates
Channel Islands Rates
Denmark Rates
Estonia Rates
Finland Rates
Net Migration Rate
Time
1950
2000
2050
2100

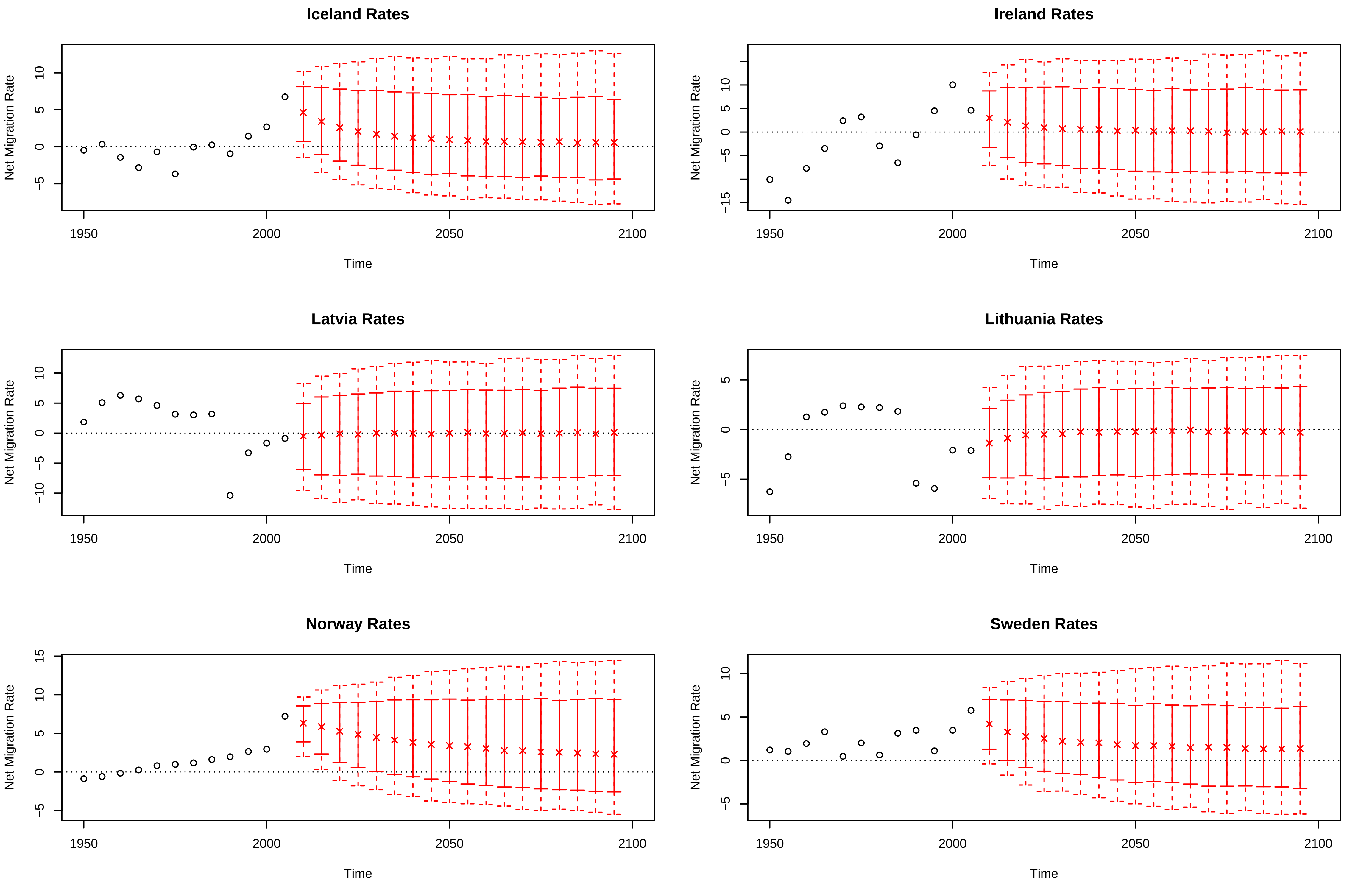

Iceland Rates
Ireland Rates
Latvia Rates
Lithuania Rates
Norway Rates
Sweden Rates
Net Migration Rate
Time
1950
2000
2050
2100

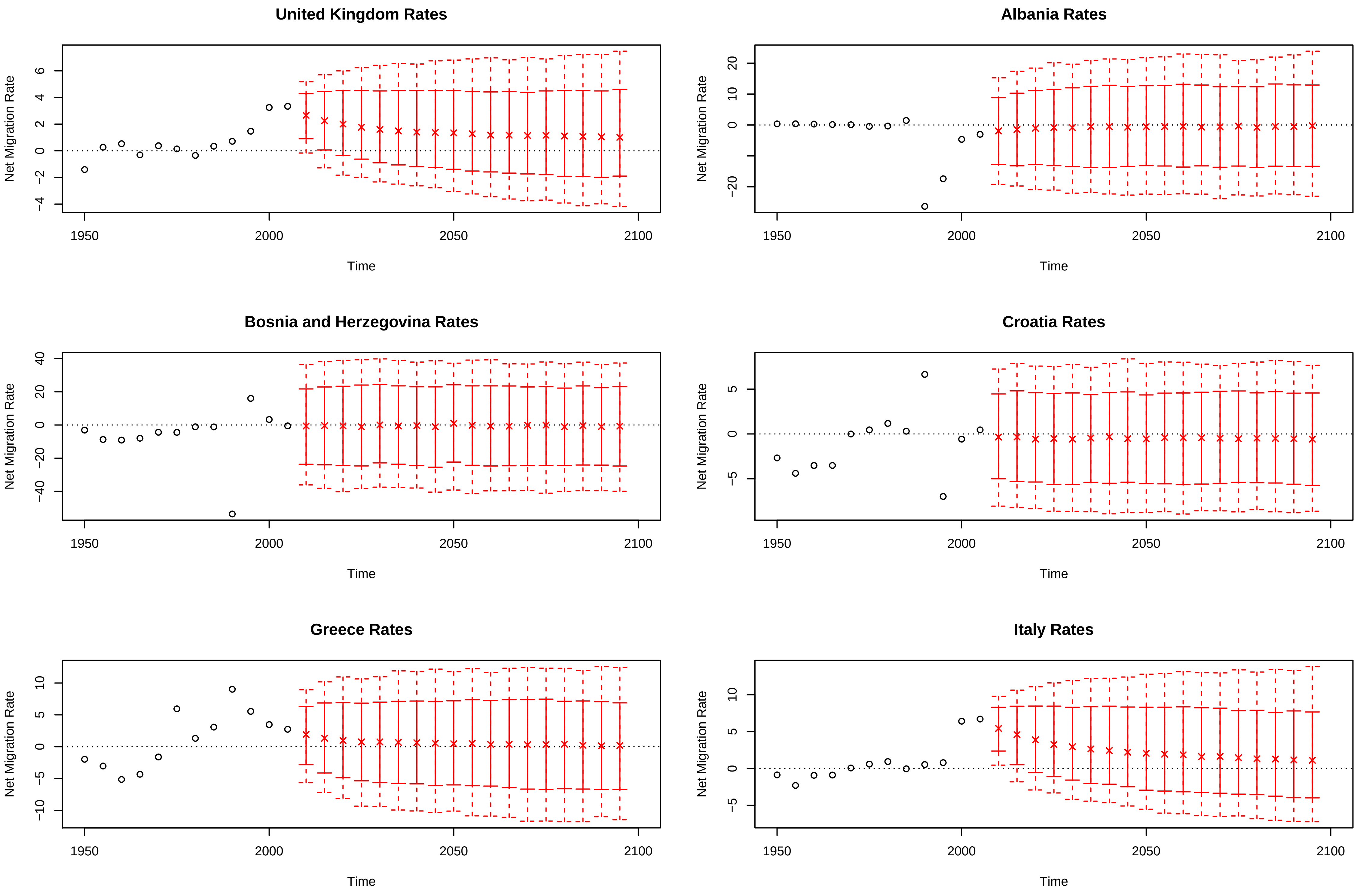

United Kingdom Rates
Albania Rates
Bosnia and Herzegovina Rates
Croatia Rates
Greece Rates
Italy Rates
Net Migration Rate
Time
1950
2000
2050
2100

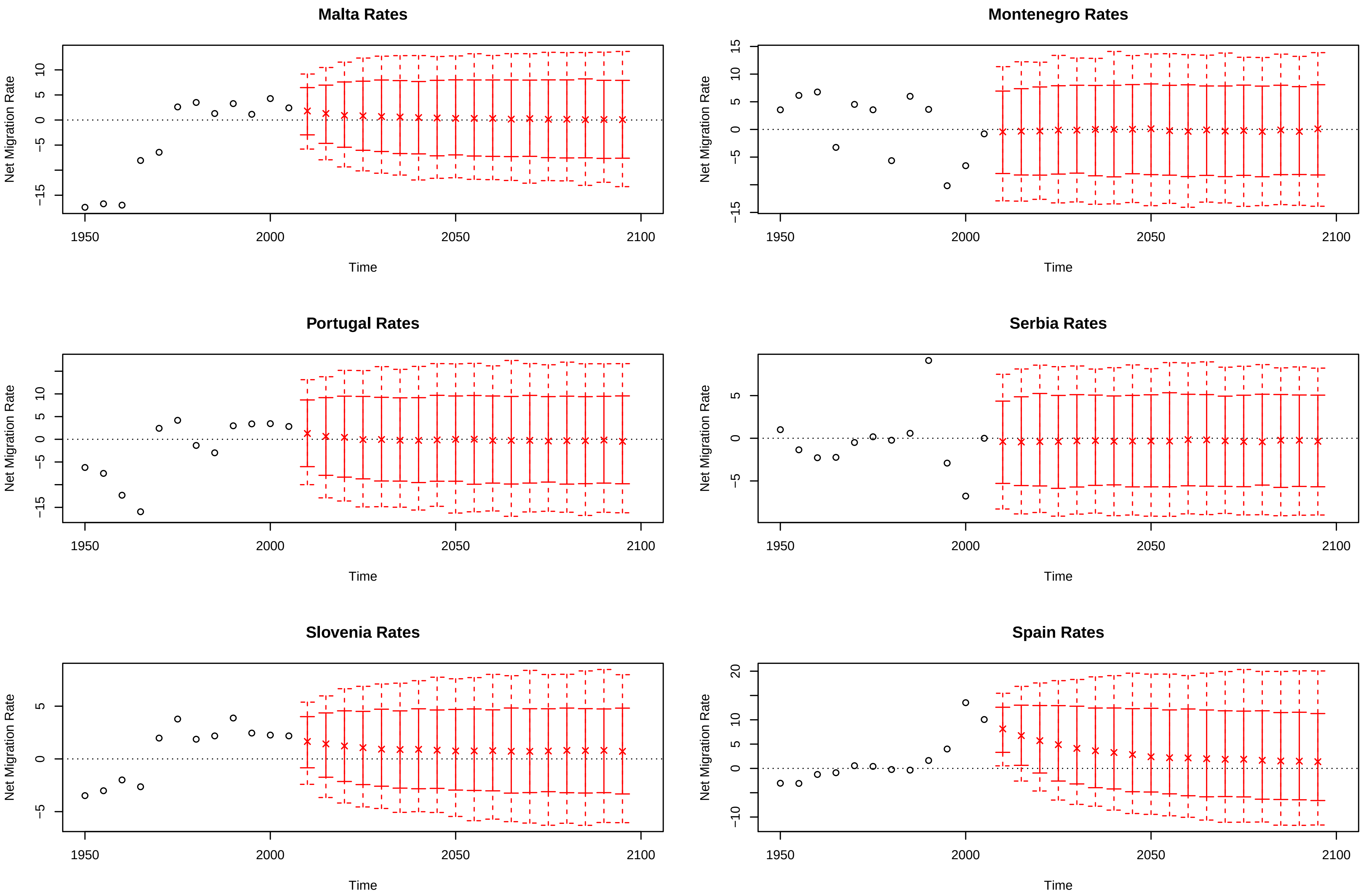
Malta Rates
Net Migration Rate
−15
−5
0
5
10
1950
2000
2050
2100
Time
Montenegro Rates
Net Migration Rate
−15
−5
0
5
10
15
1950
2000
2050
2100
Time
Portugal Rates
Net Migration Rate
−15
−5
0
5
10
1950
2000
2050
2100
Time
Serbia Rates
Net Migration Rate
−5
0
5
1950
2000
2050
2100
Time
Slovenia Rates
Net Migration Rate
−5
0
5
1950
2000
2050
2100
Time
Spain Rates
Net Migration Rate
−10
0
5
10
20
1950
2000
2050
2100
Time

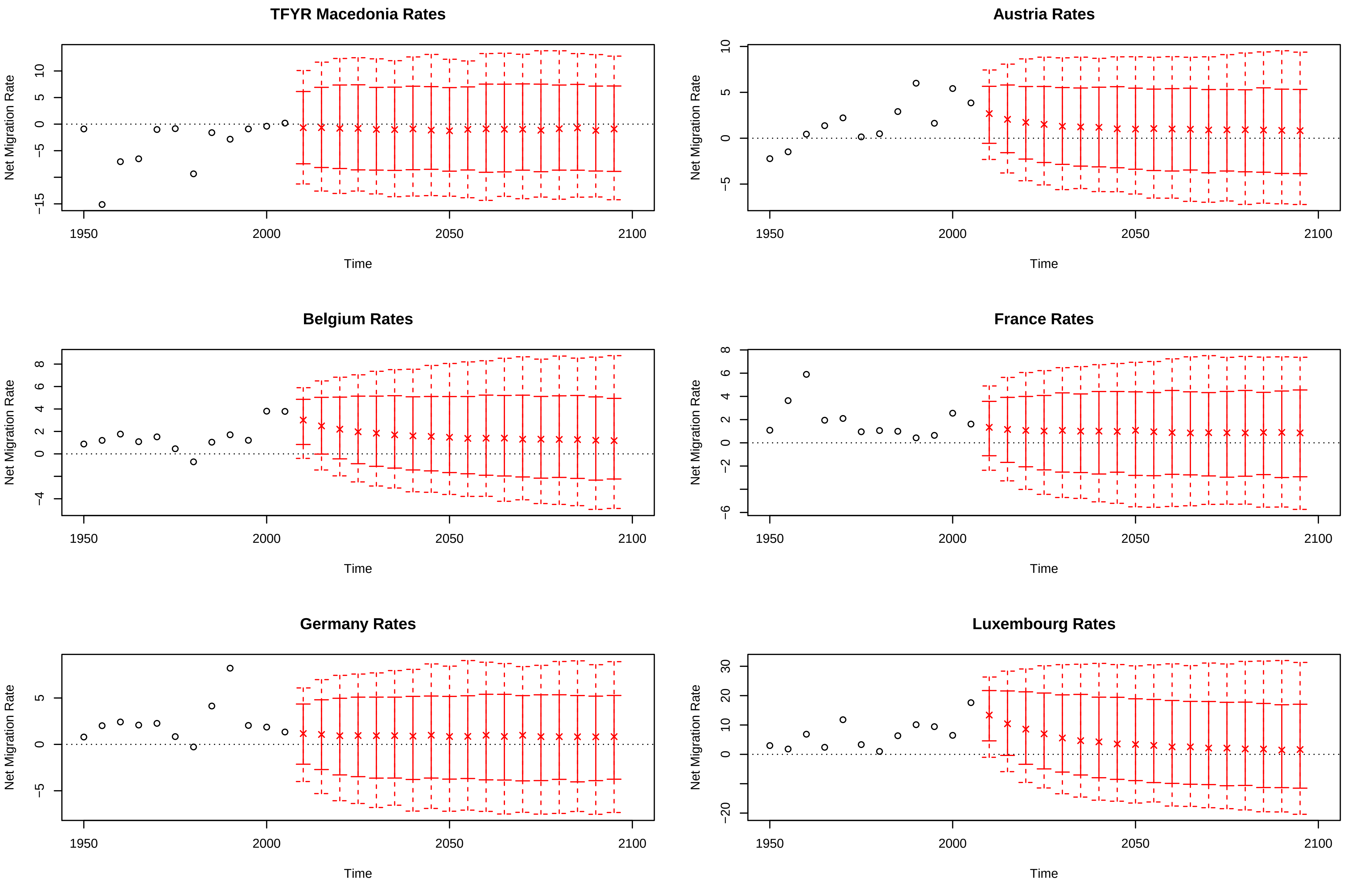
TFYR Macedonia Rates
Net Migration Rate
−15
−5
0
5
10
1950
2000
2050
2100
Time
Austria Rates
Net Migration Rate
−5
0
5
10
1950
2000
2050
2100
Time
Belgium Rates
Net Migration Rate
−4
0
2
4
6
8
1950
2000
2050
2100
Time
France Rates
Net Migration Rate
−6
−2
0
2
4
6
8
1950
2000
2050
2100
Time
Germany Rates
Net Migration Rate
−5
0
5
1950
2000
2050
2100
Time
Luxembourg Rates
Net Migration Rate
−20
0
10
20
30
1950
2000
2050
2100
Time

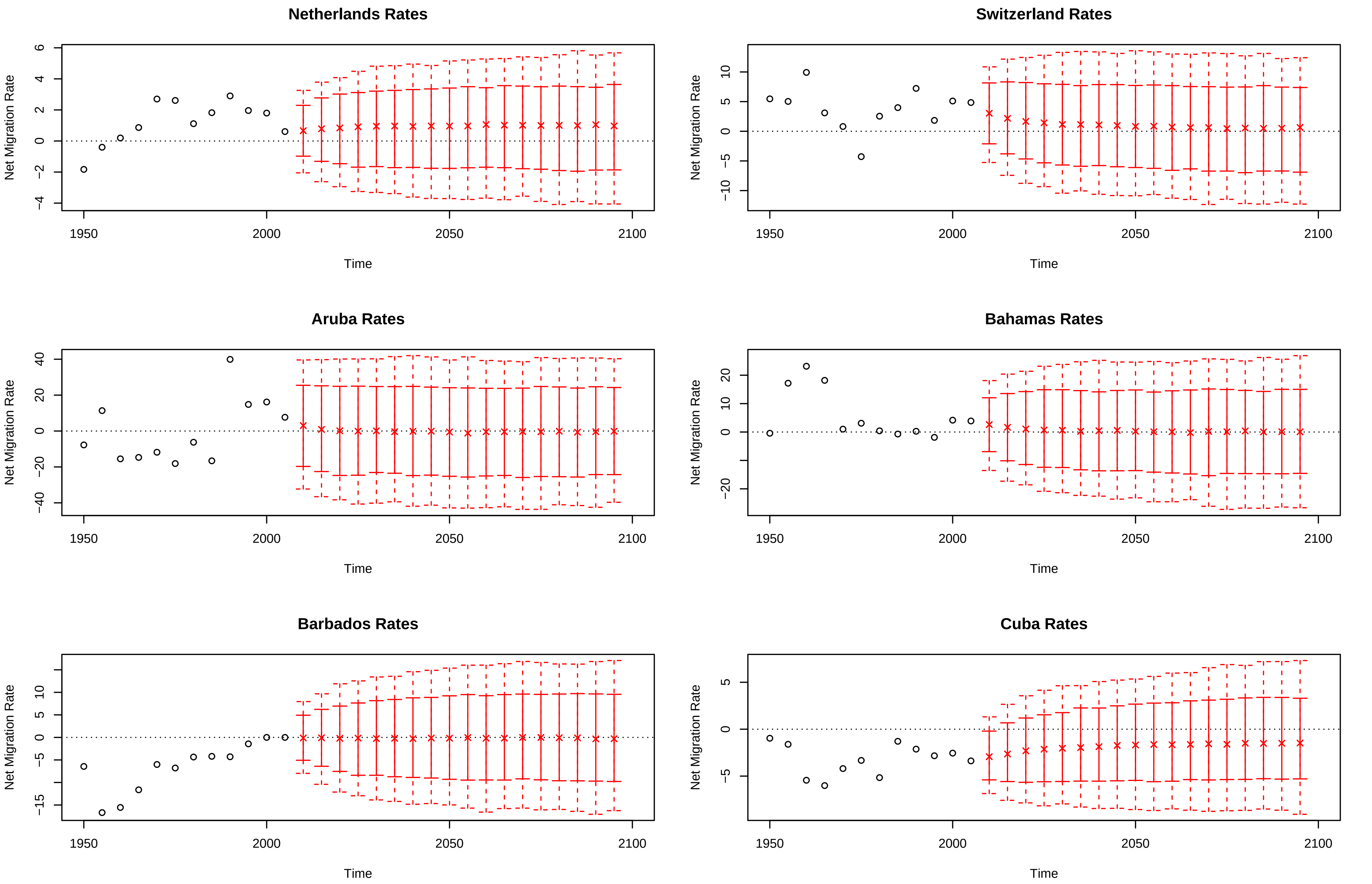

Netherlands Rates
Switzerland Rates
Aruba Rates
Bahamas Rates
Barbados Rates
Cuba Rates
Net Migration Rate
Time
1950
2000
2050
2100

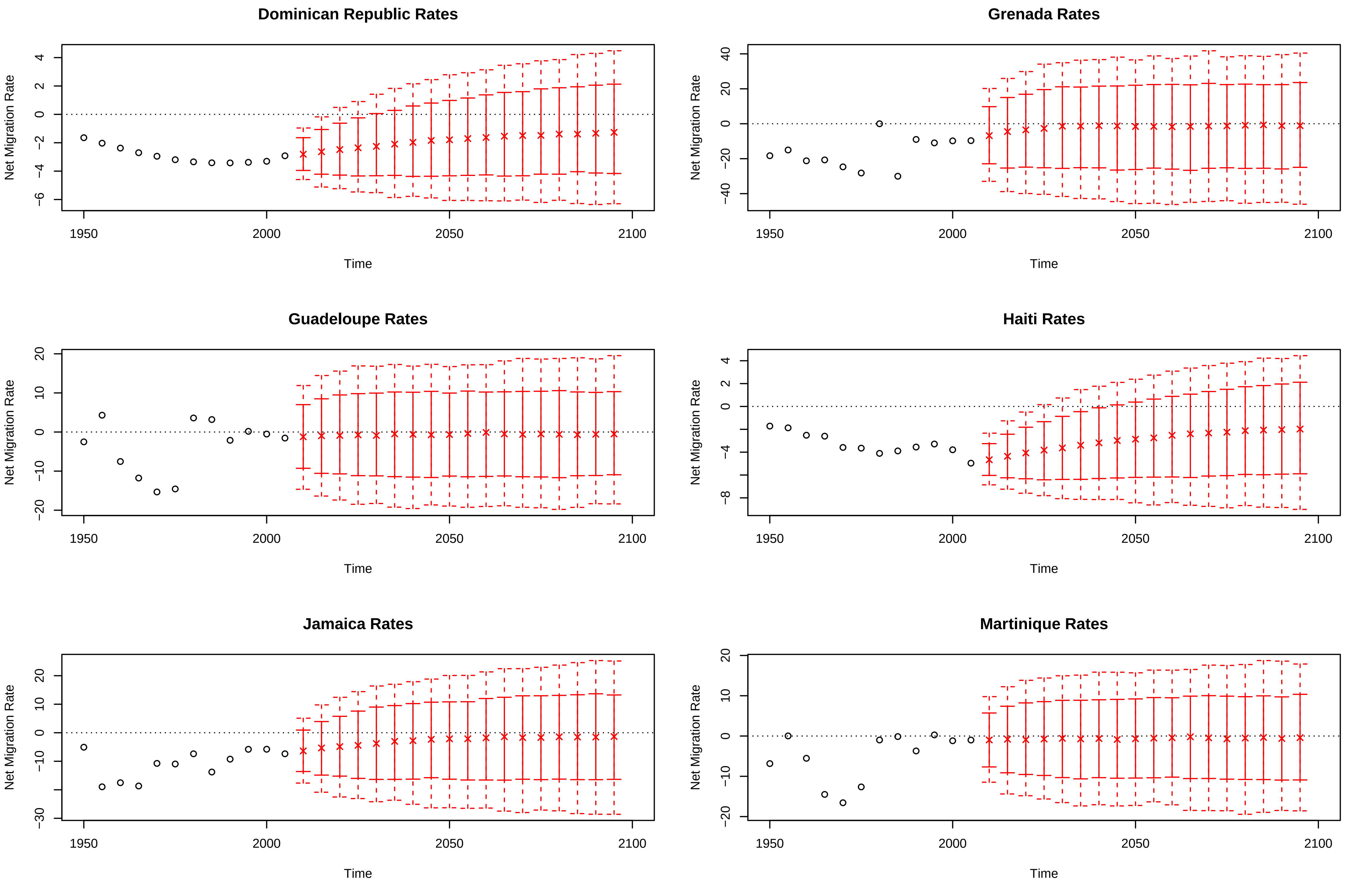
Dominican Republic Rates
Grenada Rates
Guadeloupe Rates
Haiti Rates
Jamaica Rates
Martinique Rates
Net Migration Rate
Time
1950
2000
2050
2100

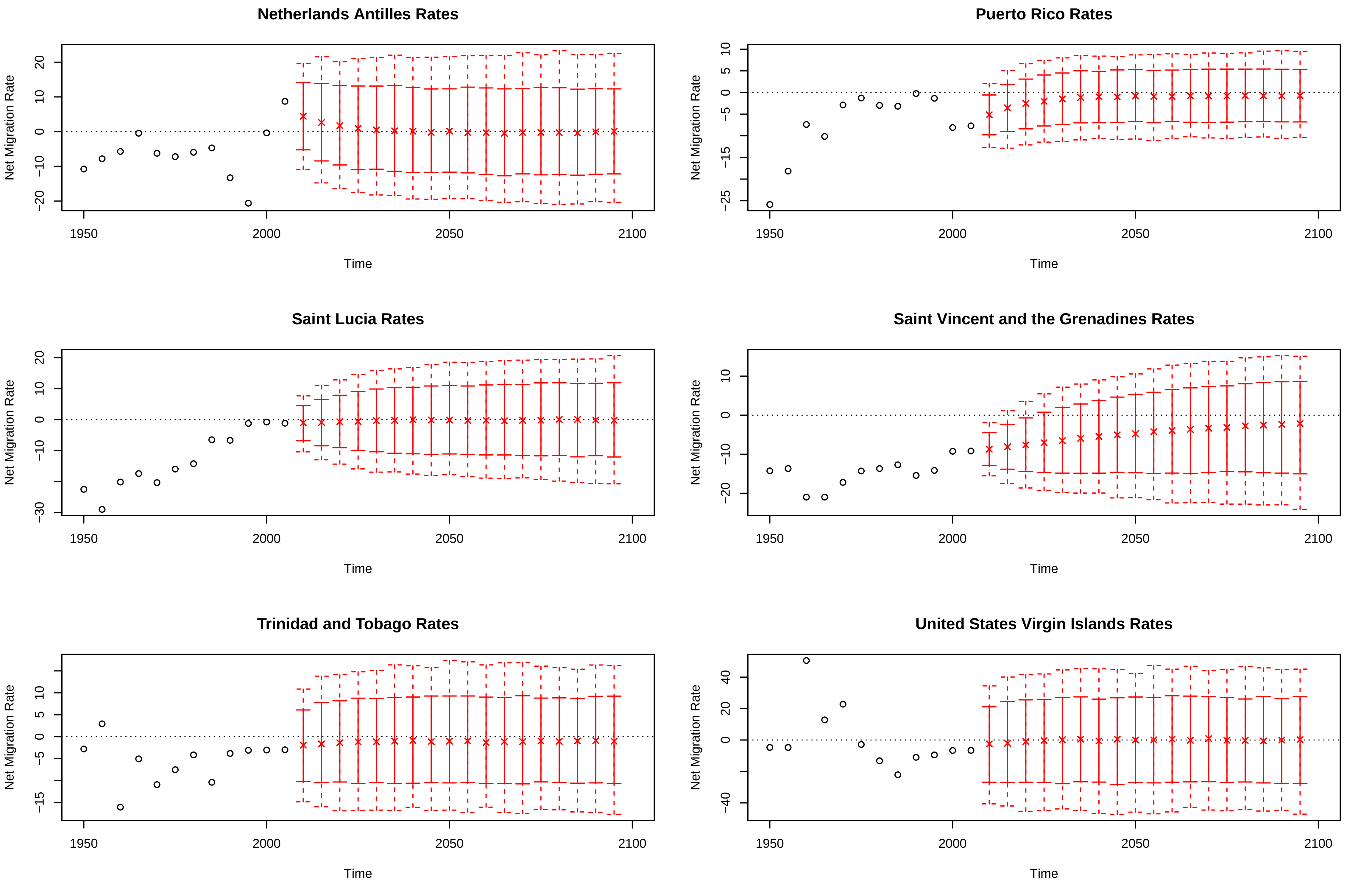

Netherlands Antilles Rates
Puerto Rico Rates
Saint Lucia Rates
Saint Vincent and the Grenadines Rates
Trinidad and Tobago Rates
United States Virgin Islands Rates
Net Migration Rate
Time
1950
2000
2050
2100

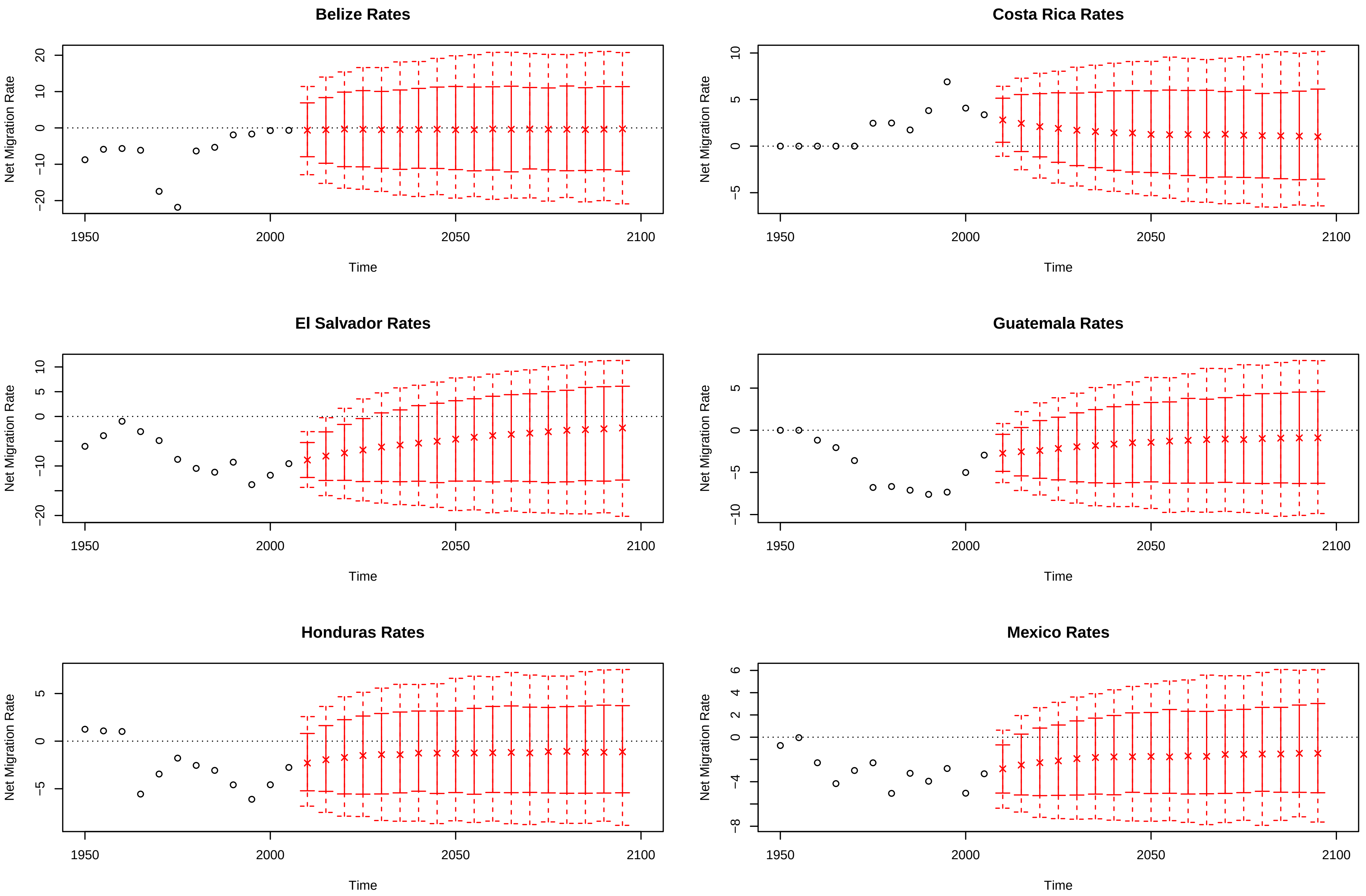
Belize Rates
Costa Rica Rates
El Salvador Rates
Guatemala Rates
Honduras Rates
Mexico Rates
Net Migration Rate
Time
1950
2000
2050
2100

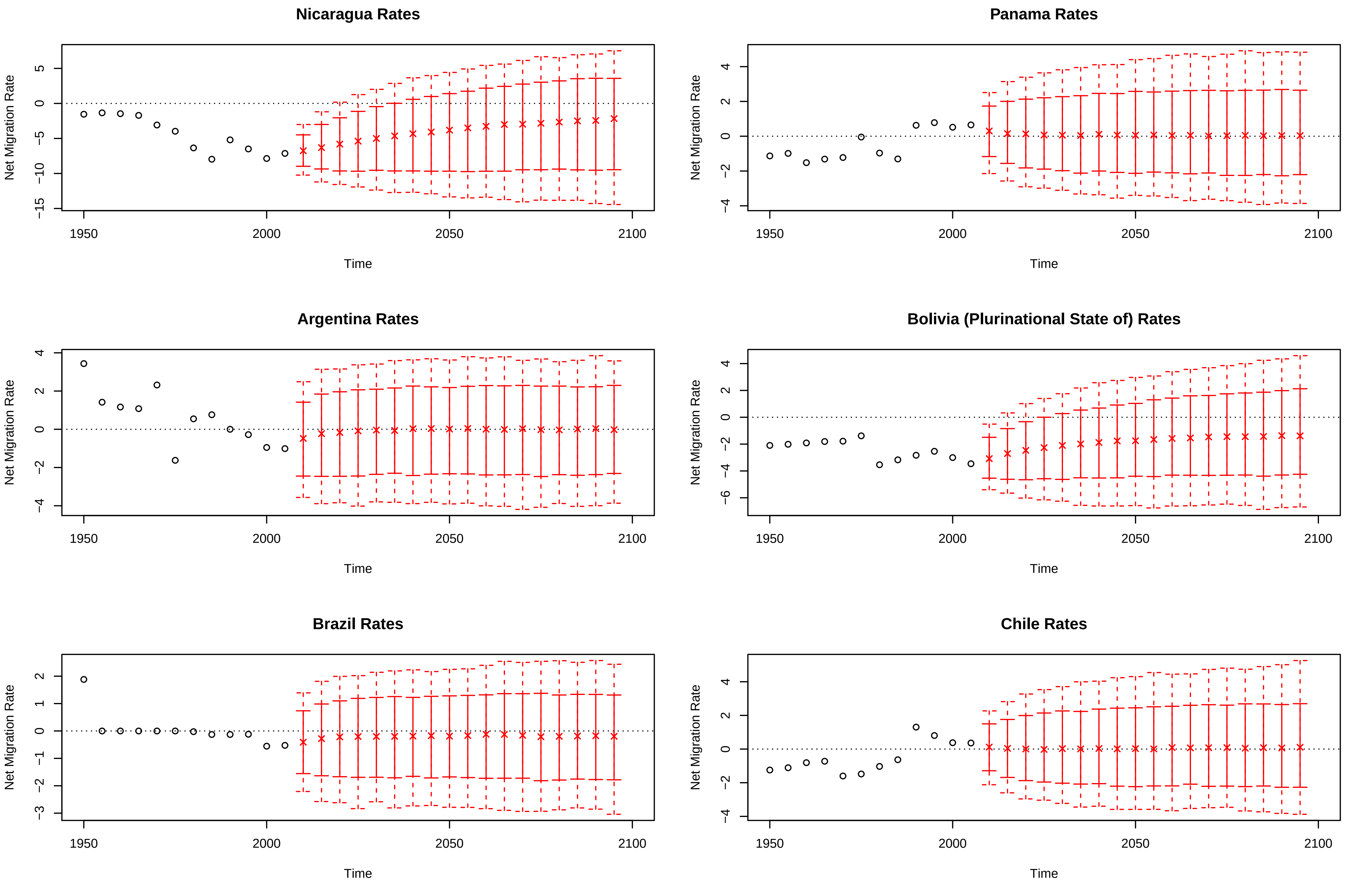

Nicaragua Rates
Panama Rates
Argentina Rates
Bolivia (Plurinational State of) Rates
Brazil Rates
Chile Rates
Net Migration Rate
Time
1950
2000
2050
2100

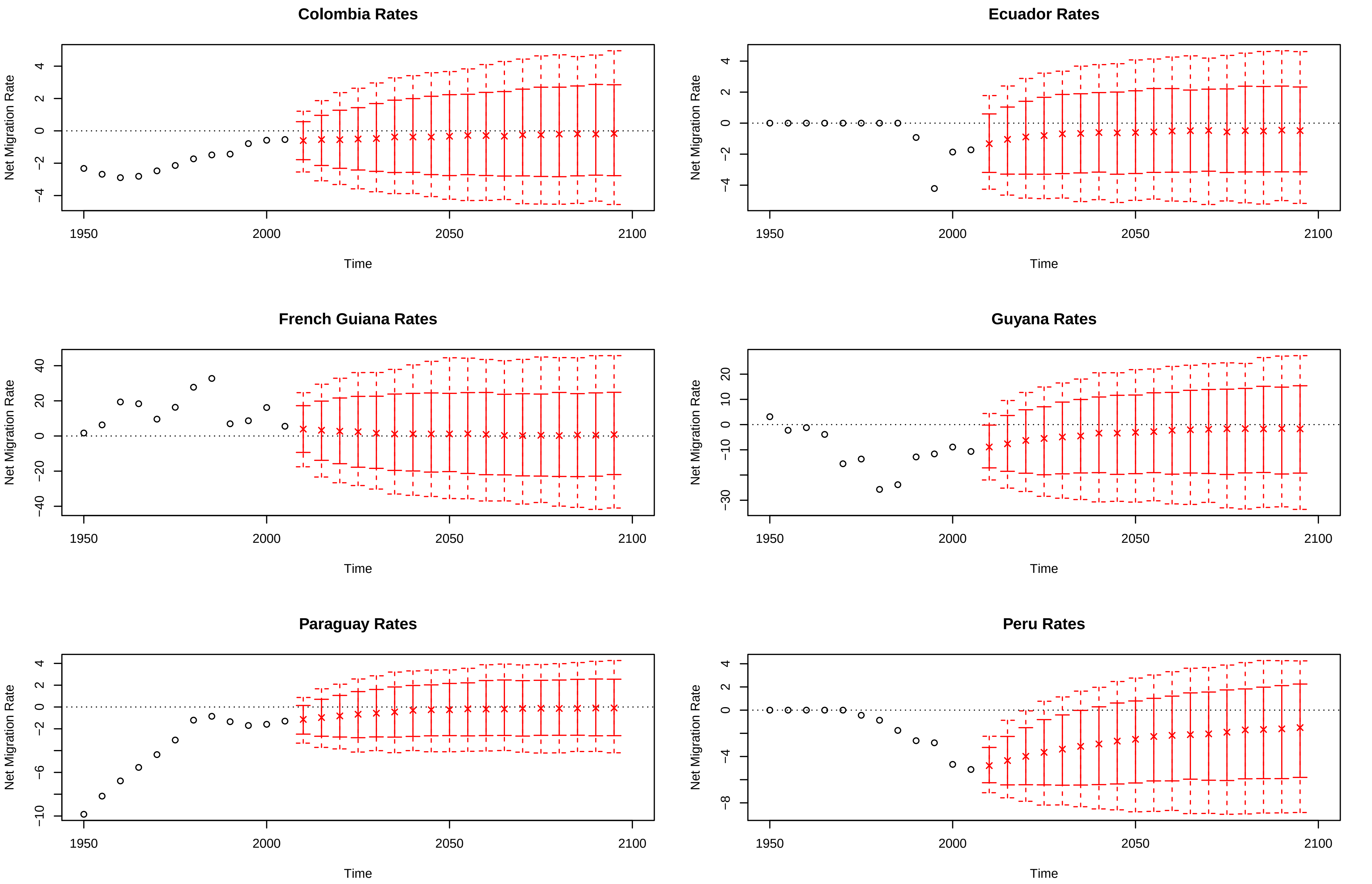

Colombia Rates
Ecuador Rates
French Guiana Rates
Guyana Rates
Paraguay Rates
Peru Rates
Net Migration Rate
Time
1950
2000
2050
2100

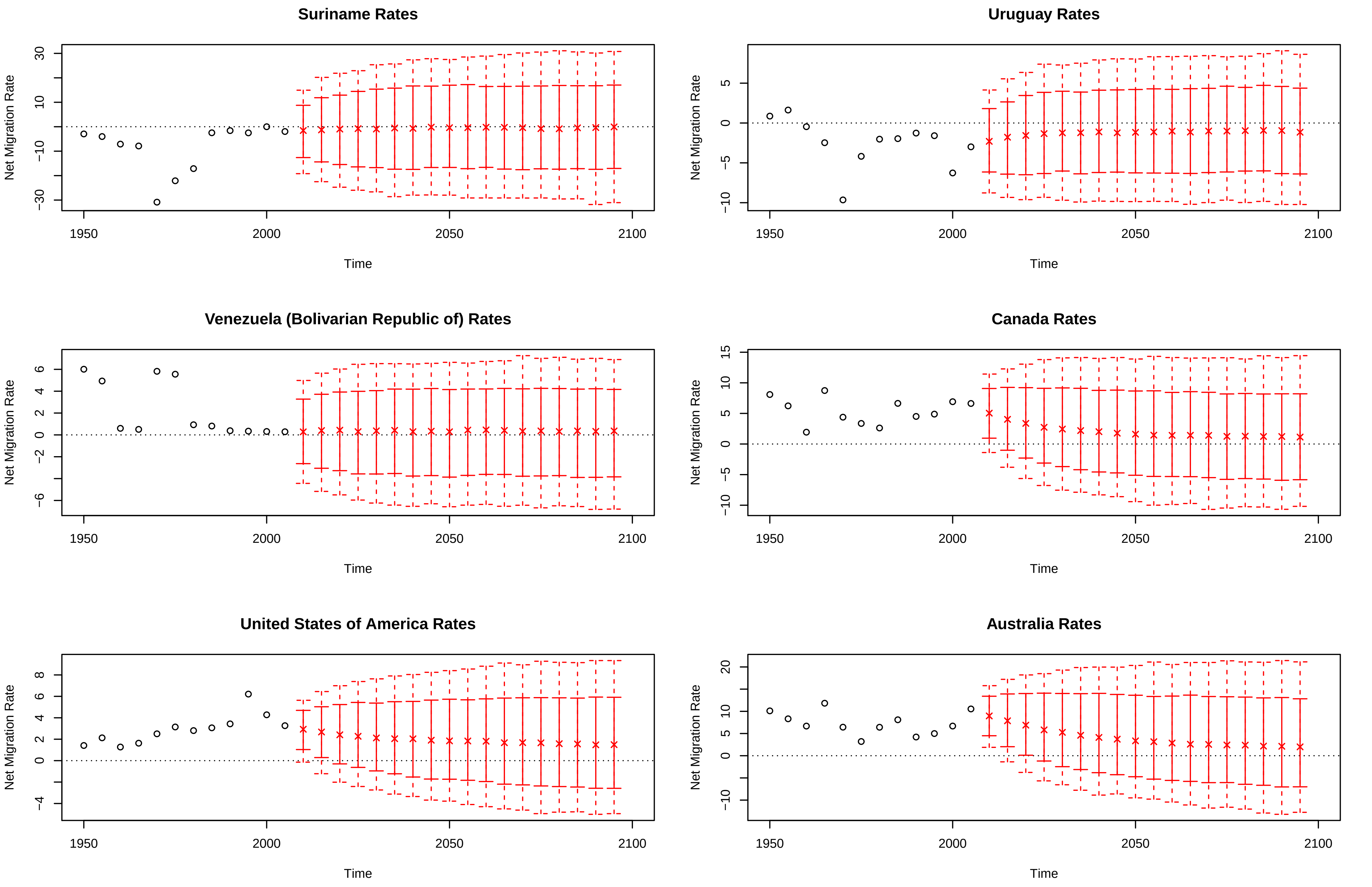
Suriname Rates
Net Migration Rate
30
10
−10
−30
1950
2000
2050
2100
Time
Uruguay Rates
Net Migration Rate
5
0
−5
−10
1950
2000
2050
2100
Time
Venezuela (Bolivarian Republic of) Rates
Net Migration Rate
6
4
2
0
−2
−6
1950
2000
2050
2100
Time
Canada Rates
Net Migration Rate
15
10
5
0
−5
−10
1950
2000
2050
2100
Time
United States of America Rates
Net Migration Rate
8
6
4
2
0
−4
1950
2000
2050
2100
Time
Australia Rates
Net Migration Rate
20
10
5
0
−10
1950
2000
2050
2100
Time

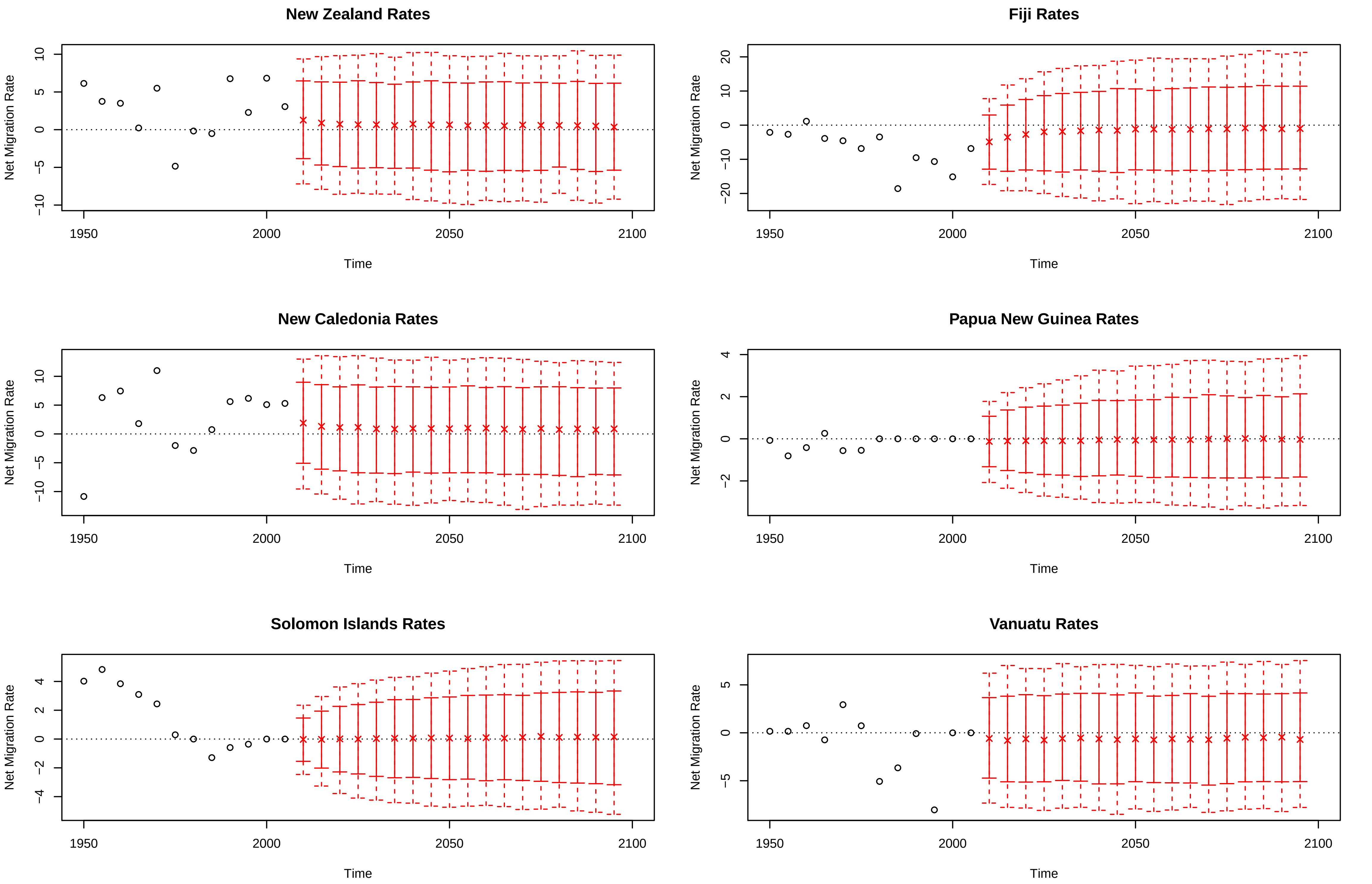
New Zealand Rates
Fiji Rates
New Caledonia Rates
Papua New Guinea Rates
Solomon Islands Rates
Vanuatu Rates
Net Migration Rate
Time
1950
2000
2050
2100

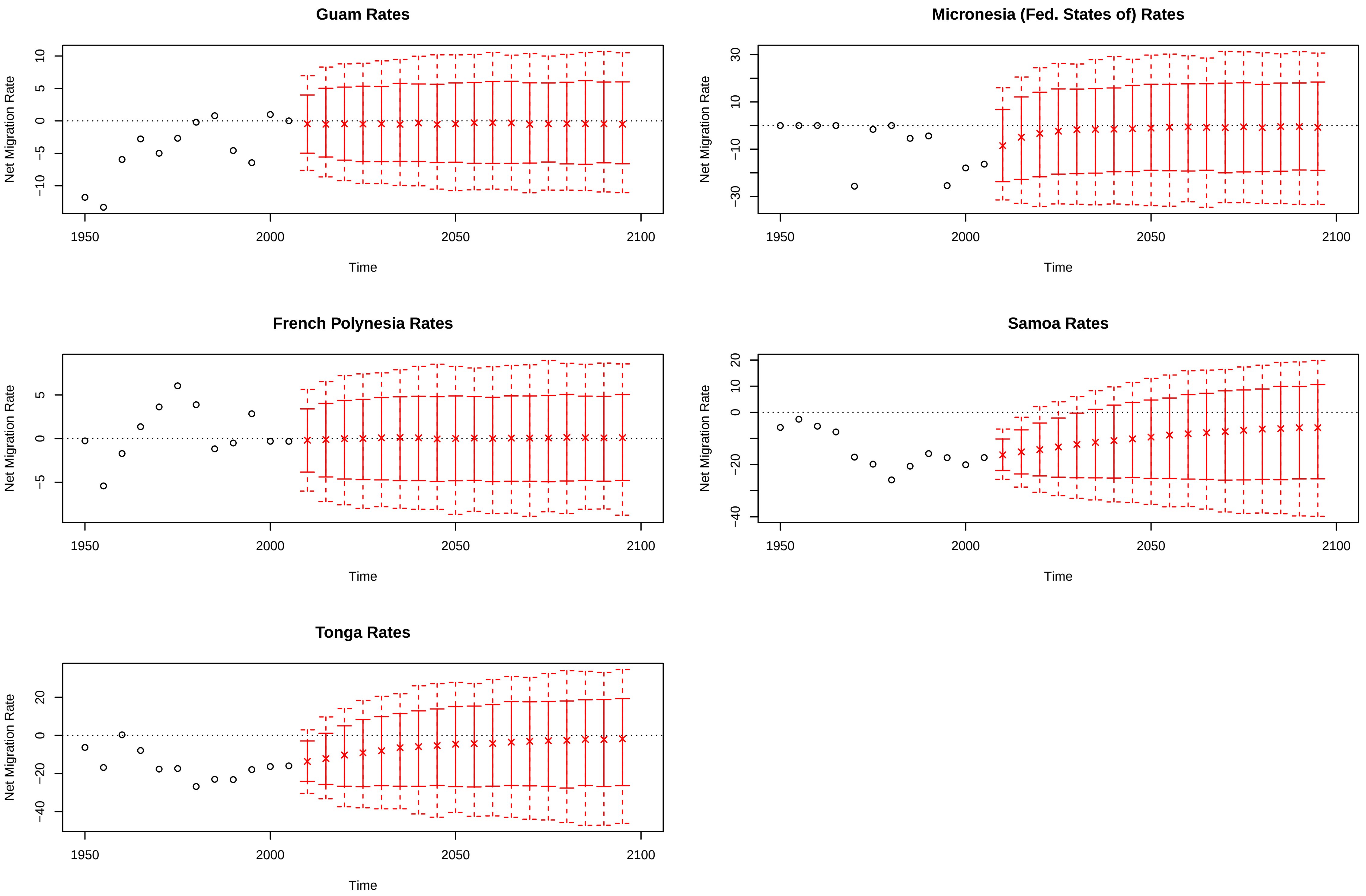

Guam Rates
Micronesia (Fed. States of) Rates
French Polynesia Rates
Samoa Rates
Tonga Rates
Net Migration Rate
Time
1950
2000
2050
2100
